# Left ventricular myocardial molecular profile of human diabetic ischaemic cardiomyopathy

**DOI:** 10.1038/s44321-025-00281-9

**Published:** 2025-08-04

**Authors:** Benjamin Hunter, Yunwei Zhang, Dylan Harney, Holly McEwen, Yen Chin Koay, Michael Pan, Cassandra Malecki, Jasmine Khor, Robert D Hume, Giovanni Guglielmi, Alicia Walker, Shashwati Dutta, Vijay Rajagopal, Anthony Don, Mark Larance, John F O’Sullivan, Jean Y H Yang, Sean Lal

**Affiliations:** 1 Precision Cardiovascular Laboratory, The University of Sydney, Sydney, NSW Australia; 2 Charles Perkins Centre, The University of Sydney, Sydney, NSW Australia; 3 School of Medical Sciences, Faculty of Medicine and Health, The University of Sydney, Sydney, NSW Australia; 4 School of Mathematics and Statistics, Faculty of Science, The University of Sydney, Sydney, NSW Australia; 5 Sydney Precision Data Science Centre, Faculty of Science, The University of Sydney, Sydney, NSW Australia; 6 School of Biomedical Sciences and Pharmacy, The University of Newcastle, Callaghan, NSW Australia; 7 School of Mathematics and Statistics, The University of Melbourne, Parkville, VIC Australia; 8 ARC Centre of Excellence for the Mathematical Analysis of Cellular Systems, The University of Melbourne, Parkville, VIC, Australia; 9 Centre for Heart Failure and Diseases of the Aorta, The Baird Institute for Applied Heart and Lung Surgical Research, Sydney, NSW Australia; 10 Department of Biomedical Engineering, School of Chemical and Biomedical Engineering, Faculty of Engineering and Information Technology, The University of Melbourne, Parkville, VIC Australia; 11 School of Mathematics, University of Birmingham, Birmingham, B15 2TT UK; 12 Baker Department of Cardiometabolic Health, Faculty of Medicine, Dentistry, and Health Sciences, The University of Melbourne, Parkville, VIC Australia; 13 Graeme Clark Institute of Biomedical Engineering, The University of Melbourne, Parkville, VIC Australia; 14 Central Clinical School, Sydney Medical School, Faculty of Medicine and Health, The University of Sydney, Sydney, NSW Australia; 15 Department of Cardiology, Royal Prince Alfred Hospital, Camperdown, NSW Australia; 16 Faculty of Medicine, TU Dresden, Dresden, Germany

**Keywords:** Human Myocardium, Ischaemic Cardiomyopathy, Diabetes, Multi-omics, Confocal Microscopy, Cardiovascular System, Chromatin, Transcription & Genomics, Proteomics

## Abstract

Ischaemic cardiomyopathy is the most common cause of heart failure and often coexists with diabetes mellitus, which worsens patient symptom burden and outcomes. Yet, their combined effects are seldom investigated and are poorly understood. To uncover the influencing molecular signature defining ischaemic cardiomyopathy with diabetes, we performed multi-omic analyses of ischaemic and non-ischaemic cardiomyopathy with and without diabetes against healthy age-matched donors. Tissue was sourced from pre-mortem human left ventricular myocardium. Fatty acid transport and oxidation proteins were most downregulated in ischaemic cardiomyopathy with diabetes relative to donors. However, the downregulation of acylcarnitines, perilipin, and ketone body, amino acid, and glucose metabolising proteins indicated lipid metabolism may not be entirely impaired. Oxidative phosphorylation, oxidative stress, myofibrosis, and cardiomyocyte cytoarchitecture also appeared exacerbated principally in ischaemic cardiomyopathy with diabetes. These findings indicate that diabetes confounds the pathological phenotype in heart failure, and the need for a paradigm shift regarding lipid metabolism.

## Synopsis

Molecular profiling of failing human hearts with and without diabetes revealed that diabetes exacerbates mitochondrial dysfunction and extracellular matrix remodelling, especially in ischaemic cardiomyopathy.

- A multi-omic analysis was performed on pre-mortem human left ventricular myocardium from ischaemic and non-ischaemic cardiomyopathy (ICM and NICM) with and without diabetes (DM and No DM), and age-matched healthy donors.
- Mitochondrial complex I protein subunits were significantly more down-regulated in ICM-DM compared to other heart failure groups.
- Lipidomic analysis revealed that 15 medium to very long-chain acylcarnitines were down-regulated only in ICM-DM.
- RNA-protein co-regulation and up-regulation of ECM-associated genes, including COL1A2 and AEBP1, were observed specifically in ICM-DM.
- ATP2A2 and ALDOA proteins were disorganised and down-regulated in ICM-DM cardiomyocytes, consistent with contractile and metabolic dysfunction.

## Abstract (web summary)

Molecular profiling of failing human hearts with and without diabetes revealed that diabetes exacerbates mitochondrial dysfunction and extracellular matrix remodelling, especially in ischaemic cardiomyopathy.

## Graphical abstract

**Figure:**
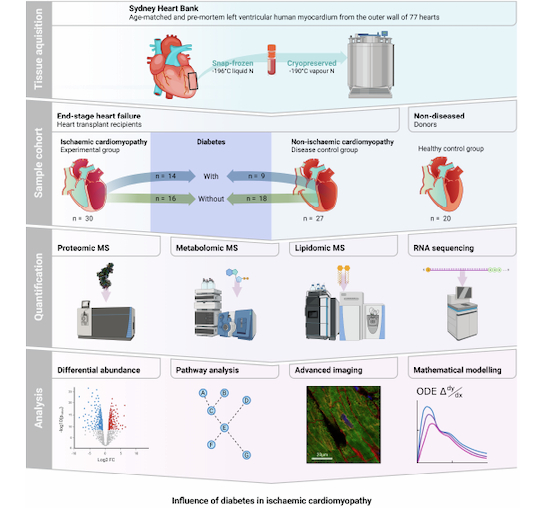

**Figure:**
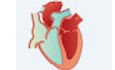

## Introduction

Heart failure (HF) is a global epidemic and leading cause of death affecting 1–2% adults in developed countries, with the most common causes being ischaemic cardiomyopathy (ICM) and non-ischaemic (dilated) cardiomyopathy (NICM) (Groenewegen et al, 2020; Khan et al, 2020; McKenna et al, 2017). ICM is an acquired cardiomyopathy typically due to coronary artery disease (CAD) which primarily affects the left ventricle (LV) (Buja and Vander Heide, 2016; Elosua et al, 2014). NICM can involve both ventricles and is considered to arise from a mix of acquired and genetic aetiologies (Herman et al, 2012; McNally and Mestroni, 2017; Weintraub et al, 2017).

ICM and NICM both express ventricular dilatation and dysfunction and are the most common causes for heart transplantation (Buja and Vander Heide, 2016; McKenna et al, 2017; Stehlik et al, 2011; Weintraub et al, 2017). Yet, ICM transplant recipients have an elevated risk of acute cardiac events and a reduced 10-year survival rate (50%) compared to NICM (84%), often due to underlying risk factors promoting CAD progression, including hypertension and metabolic syndrome (Guddeti et al, 2014). These risk factors also contribute to the development of type 2 diabetes mellitus (DM), which is often concomitant with ICM in HF patients (Bell and Goncalves, 2019; Einarson et al, 2018; Perrone-Filardi et al, 2015). DM, in turn, is associated with dysregulation of fatty acid oxidation (FAO) pathways, with fatty acids being a principal energy source for the heart (Flam et al, 2022; Leguisamo et al, 2012; Lopaschuk et al, 2021; Neglia et al, 2007; Nyman et al, 2013). These metabolic processes, in addition to the extracellular matrix (ECM) and contractile complexes of the myocardium, have not been comprehensively investigated in human hearts, with ICM and DM seldom investigated together but frequently studied in isolation (Jia et al, 2018; Paolillo et al, 2019). Given that DM exacerbates HF symptoms and increases mortality in HF (Cubbon et al, 2013; Ebong et al, 2013), there is a need to understand the impact of DM on HF.

Therefore, the aim of this study was to perform an extensive multi-omic analyses on cryopreserved pre-mortem human left ventricular myocardium to uncover the influence of DM in end-stage HF and to delineate the molecular characteristics of ICM with DM (ICM-DM), thereby creating a comprehensive molecular resource of DM-related HF.

## Results

**Figure 1:**
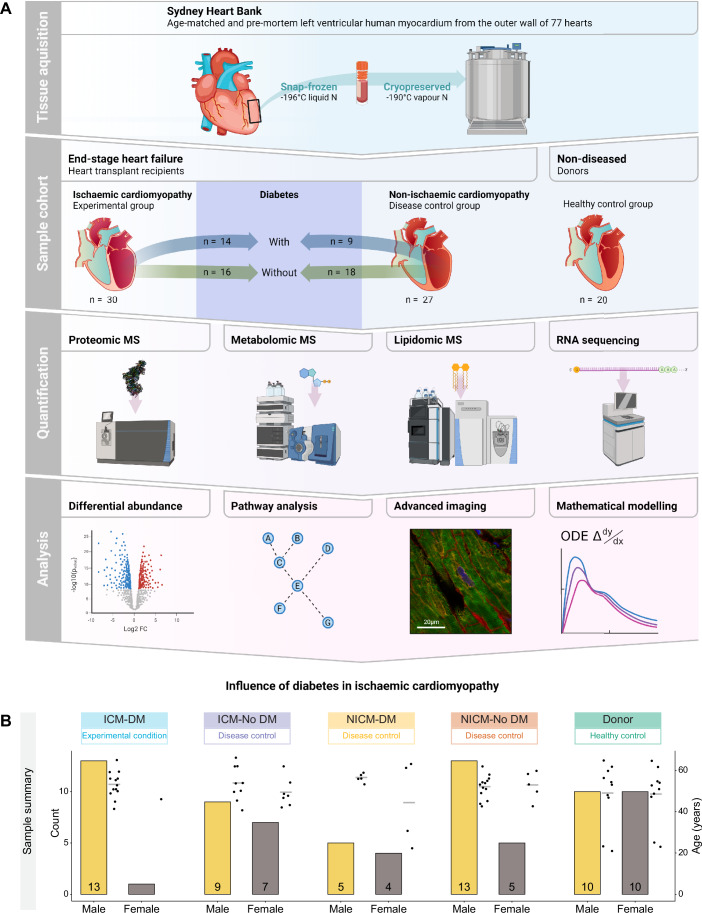
Overview of experimental design, workflow summary, and sample summary. (**A**) Workflow overview. Created with BioRender.com. (**B**) Sample summary displaying sex and age of conditions.

Myocardial multi-omic mass spectrometry (MS) differential analyses were performed on all end-stage HF conditions vs healthy age-matched donors (AMD) wherein DM and no DM groups were contrasted against each other (Fig. 1A). A sample summary is depicted in Fig. 1B. Demographic data can be seen in Dataset EV1. Multidimensional scaling (MDS) plots were used to visualise individual and group similarity via a distance matrix for each MS analysis (Fig. EV1A–L).

**Figure EV1:**

Sample visualisation of normalised and log_2_ transformed proteomic, metabolomic and lipidomic mass spectrometry data from human myocardium in all conditions. (**A**–**L**) Multidimensional scaling (MDS) plots, a tool to visualise high dimensional data in two dimensions, were used to iteratively separate samples (each point) in distance based on their dissimilarity of paired data (proteins, metabolites, and lipids; dimensions) calculated as the leading log_2_ fold change (FC, average root-mean-square of the largest log_2_ FCs). All protein/metabolite/lipid MDS plots are the same but vary in samples highlighted. (**A**–**C**) All conditions highlighted. (**D**–**F**) Heart failure (HF) conditions and healthy donors highlighted. (**G**–**I**) Ischaemic cardiomyopathy with diabetes (ICM-DM) and without diabetes (ICM-No DM) highlighted. (**J**–**L**) Non-ischaemic cardiomyopathy with diabetes (NICM-DM) and without diabetes (NICM-No DM) highlighted.

### Differential molecular analysis of ischaemic cardiomyopathy with diabetes

**Figure 2:**
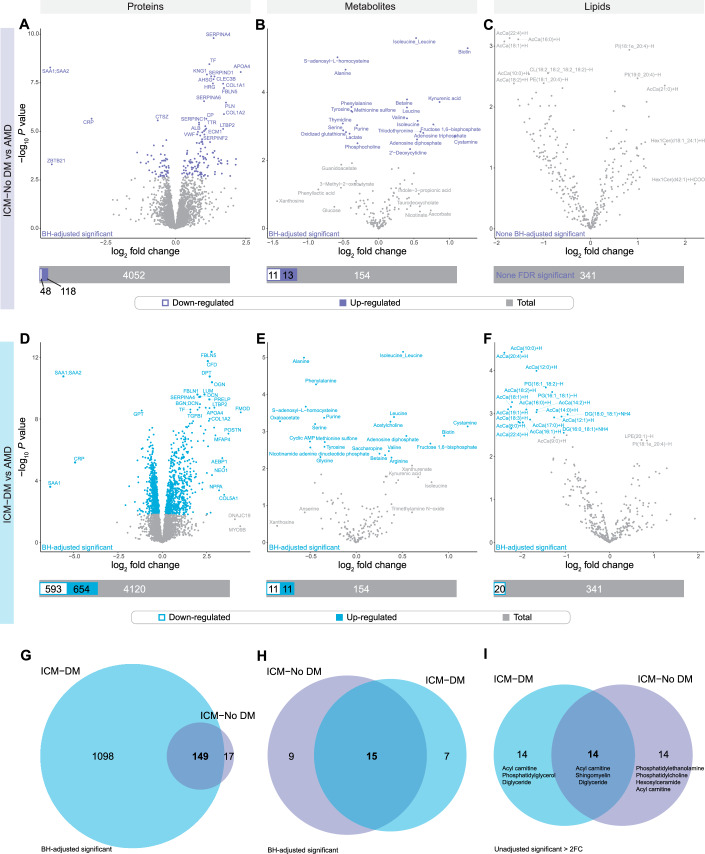
Human left ventricular myocardial differential analysis in protein, metabolite, and lipid abundance between ischaemic cardiomyopathy with (ICM-DM) and without diabetes (ICM-No DM) and age-matched donors (AMD). (**A**–**F**) Differential abundance was determined following Benjamini–Hochberg false discovery rate adjustment (FDR) of *P* values (FDR < 0.05). Analyses were performed using a moderated *t* test with the limma package (version 3.56.2) in R (version 4.3.1) following log_2_ transformation. ICM-DM *n* = 14, ICM-No DM *n* = 16, AMD *n* = 20 (proteomics and metabolomics) and 19 (lipidomics). Superimposed bar plots summarise the number of significantly downregulated (white-filled bar) and upregulated (colour-filled bar) molecules relative to the total number of molecules analysed (grey bar). (**A**–**C**) ICM-No DM vs AMD. (**D**–**F**) ICM-DM vs AMD. (**G**, **H**) ICM-DM and ICM-No DM vs AMD FDR significantly differentially abundant proteins and metabolites. (**I**) ICM-DM vs AMD and ICM-No DM vs AMD unadjusted significant (*P* < 0.05) lipids, which were also > ±2-fold change (FC, in either direction). Lipid classes annotated in order of the number of lipids which were unadjusted significantly.

Analyses of HF conditions with and without diabetes compared against AMD can be found in Appendix Fig. S1 and Datasets EV2–EV7. When focused on individual HF conditions vs AMD, proteomic analysis resulted in 593 downregulated and 654 upregulated proteins in ICM-DM of which, 1098 were only differentially abundant (DA) in the ICM-DM group of ICM, such as regulator of microtubule dynamics (RMDN3), α-actinin 1 (ACTN1), mitochondrial isocitrate dehydrogenase (IDH2), and mitochondrial acetyl-CoA acetyltransferase (ACAT1) (Fig. 2A,D,G; Datasets EV8 and EV11). Cross-comparison of HF conditions vs AMD identified serum amyloid A1 (SAA1) was uniquely and greatly downregulated in ICM-DM. Other highly DA proteins specific to ICM-DM included neogenin (NEO1), numerous NDUF complex I (CI) subunits such as NDUFS3 and muscle aldolase (ALDOA) (Fig. EV2A,D,G; Datasets EV15 and EV18). Proteins specifically DA in DM groups included multiple NDUFs, cysteine protease inhibitor FETUB, acyl-CoA oxidase (ACOX2), mitochondrial acetyl-CoA acyltransferase (ACAA2), and extracellular matrix (ECM)-related cytoskeletal protein talin 1 (TLN1). Some of the most significant ICM-DM proteins were also shared with the other HF groups, including complement factor D (CFD), glutamic-pyruvic transaminase 2 (GPT), and mitochondrial acyl-CoA synthetase (ACSS3).

**Figure EV2:**

Human left ventricular myocardial differential analysis in protein, metabolite, and lipid abundance between non-ischaemic cardiomyopathy with (NICM-DM) and without diabetes (NICM-No DM) and age-matched donors (AMD). (**A–F**) Differential abundance was determined following Benjamini–Hochberg false discovery rate adjustment (FDR) of *P* values (FDR < 0.05). Analyses were performed using a moderated *t* test with the limma package (version 3.56.2) in R (version 4.3.1) following log_2_ transformation. NICM-DM *n* = 9, NICM-No DM *n* = 18 (proteomics and metabolomics) and 17 (lipidomics), AMD *n* = 20 (proteomics and metabolomics) and 19 (lipidomics). Superimposed bar plots summarise the number of significantly downregulated (white-filled bar) and upregulated (colour-filled bar) molecules relative to the total number of molecules analysed (grey bar). (**A**–**C**) NICM-No DM vs AMD. (**D**–**F**) NICM-DM vs AMD. (**G**, **H**) NICM-DM and NICM-No DM vs AMD FDR significant differentially abundant proteins and metabolites. (**I**) NICM-DM vs AMD and NICM-No DM vs AMD unadjusted significant (*P* < 0.05) lipids which were also > ±2-FC (in either direction) for comparative purposes (significance not accepted). Lipid classes annotated in order of the number of lipids which were unadjusted significant.

In the metabolomic analysis, there were 11 downregulated and 11 upregulated metabolites in ICM-DM vs AMD of which 7 were DA in ICM-DM only in the ICM group. Oxaloacetate, acetylcholine, nicotinamide adenine dinucleotide phosphate, glycine, arginine and cyclic adenosine monophosphate (cAMP), a second messenger for intracellular signalling, were among the metabolites uniquely DA in ICM-DM relative to the other forms of HF (Figs. 2B,E,H, EV2B,E,H, and EV4D,E; Datasets EV9, EV13, EV16, and EV19). Metabolites which were uniquely upregulated in ICM included fructose 1,6-bisphosphate and adenosine diphosphate (ADP). Alanine (downregulated) and isoleucine/leucine amino acids (upregulated) were found to be commonly DA in all HF conditions in the same direction. Cystamine featured the greatest FCs and was upregulated in ICM-DM, ICM-No DM, and NICM-No DM. NAD^+^/NADH ratio was upregulated in ICM-DM, ICM-No DM, and NICM-No DM vs AMD. NADP^+^/NADPH ratio was downregulated in ICM-DM, ICM-No DM, and NICM-No DM.

**Figure EV4:**

Myocardial differential abundance co-analyses of all heart failure conditions vs age-matched donors (AMD), relative to each other, for proteomic, metabolomic, and lipidomic mass spectrometry analyses, with a focus on ischaemic cardiomyopathy with diabetes (ICM-DM). (**A**) UpSet plot summarising significant differentially expressed proteins in all the heart failure conditions; ICM-DM, ICM without diabetes (ICM-No DM), non-ischaemic cardiomyopathy with diabetes (NICM-DM), and NICM without diabetes (NICM-No DM), vs AMD from proteomic mass spectrometry (MS). Statistical significance of differential expression was determined following Benjamini–Hochberg false discovery rate adjustment (FDR) of *P* values (FDR < 0.05). (**B**) Gene Ontology (GO) analysis by PANTHER (http://geneontology.org/, PANTHER17.0) enrichment bubble plot showing selected significantly enriched Biological Process (BP), Cellular Component (CC), and Molecular Function (MF) nodes from proteins which were significantly differentially expressed in all heart failure conditions vs AMD. Nodes with an FDR < 0.05 were considered statistically significant. This plot was the combination of two separate GO analyses; one from downregulated proteins compared to AMD (blue) and one from upregulated proteins compared to AMD (red). Enrichment count, represented as the size of the bubble, is the number of significant proteins in that particular node. GO fold enrichment is calculated as observed enrichment count/expected enrichment count from a random set of gene symbols of equal an input set size. (**C**) GO analysis by PANTHER enrichment bubble plot of selected significantly enriched nodes from proteins which were significantly expressed only in ICM-DM vs AMD. (**D**) UpSet plot summarising FDR significant differentially abundant metabolites in all the heart failure conditions vs AMD from metabolomic MS. (**E**) Heat map of the two greatest fold change (FC) metabolites in, and specific to, ICM-DM vs AMD. (**F**) Circular heatmap of all heart failure conditions log_2_ FC vs AMD quantified acylcarnitines from lipidomic MS where FDR significant differentially abundant lipids are indicated. A Lipid’s chain length was contrasted to others in colour; very-long-chain (22 or more carbon length, maroon), long-chain (13–21 carbon length, orange), and medium-chain (6–12 carbon length, pink). Short-chain acylcarnitines (2–5 carbon length) were quantified in metabolomic MS.

Identified HF-specific signature proteins and metabolites can be observed in Fig. EV4A,D and Datasets EV65–71.

Lipidomic analysis identified 20 downregulated lipids in ICM-DM, 15 of which were medium-chain (4), long-chain (10), and very-long-chain (1) acylcarnitines, as well as diacylglyerols (2) and phosphatidylglycerols (2), with no other HF condition presenting FDR significant lipid differential abundance individually (Figs. 2C,F,I, EV2C,F,I, and EV4F; Datasets EV10, EV14, EV17, and EV20).

To allow exploration of these differential analyses, we produced an interactive resource (shiny app, http://shiny.maths.usyd.edu.au/Human-Heart-ALL/).

### Enriched metabolic pathways and gene sets

**Figure 3:**
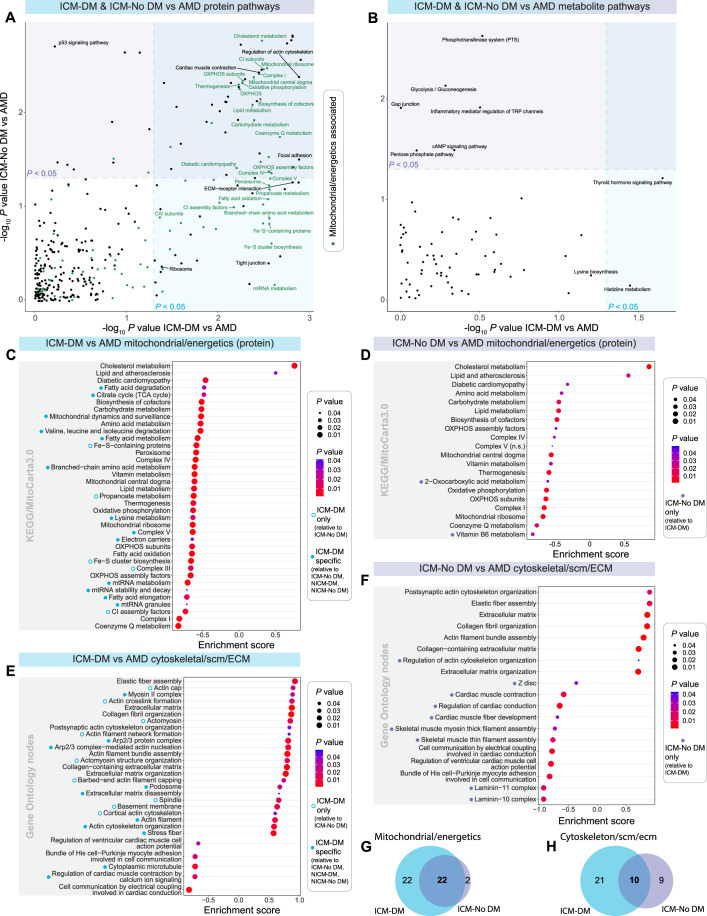
Human ischaemic cardiomyopathy with (ICM-DM) and without diabetes (ICM-No DM) vs healthy age-matched donor (AMD) protein and metabolite pathway analyses. (**A**, **B**) Scatter plots of −log_10_*P* value enriched KEGG and MitoCarta3.0 protein pathways/gene sets of ICM-DM vs AMD and ICM-No DM vs AMD where mitochondrial and energetics related pathways are coloured in green and significant (*P* < 0.05). Pathways in overlapping coloured regions were significant in both ICM-DM and ICM-No DM vs AMD. (**A**) Enriched pathways from the proteomic mass spectrometry (MS) analysis. (**B**) Enriched pathways from the metabolomic MS analysis. (**C**–**F**) Gene Set Enrichment Analysis (GSEA) enrichment bubble plots of top-ranked significantly enriched (*P* < 0.05) KEGG/MitoCarta3.0 pathways/gene sets and Gene Ontology nodes, respectively. (**C**, **D**) From (**A**). (**E**, **F**) Cytoskeletal, sarcomeric (scm), and extracellular matrix (ECM) enriched Gene Ontology Biological Process and Cellular Component nodes from selected parent nodes. (**G**) Mitochondrial/energetics-associated pathways from (**A**). (**H**) All enriched Gene Ontology nodes which produced (**E**, **F**). Gene Set Enrichment Analysis (GSEA) analyses for (**A**–**F**) were performed on normalised and transformed proteomic and metabolomic datasets using clusterProfiler (version 4.8.1).

**Figure EV3:**
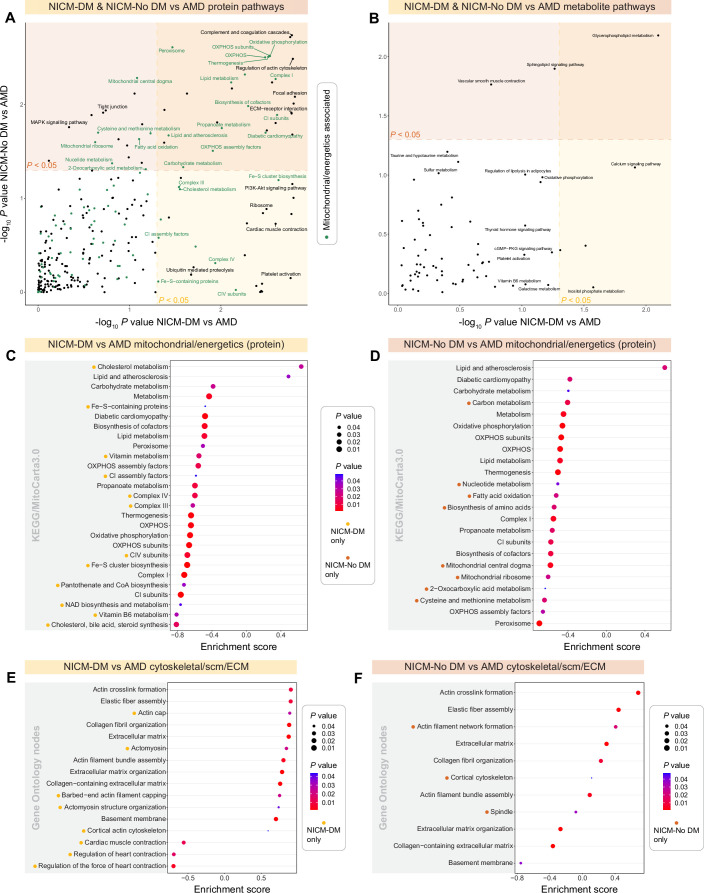
Human non-ischaemic cardiomyopathy with (NICM-DM) and without diabetes (NICM-No DM) vs age-matched donor (AMD) myocardial protein and metabolite pathway analyses. (**A**, **B**) Scatter plots of -log_10_*P* value enriched KEGG and MitoCarta3.0 protein pathways/gene sets of NICM-DM AMD and NICM-No DM where mitochondrial and energetics related pathways are coloured in green and significant (*P* < 0.05). Pathways in overlapping coloured regions were significant in both NICM-DM and NICM-No DM vs AMD. (**A**), Enriched pathways from the proteomic mass spectrometry (MS) analysis. (**B**) Enriched pathways from the metabolomic MS analysis. (**C**–**F**) Gene Set Enrichment Analysis (GSEA) enrichment bubble plots of top-ranked significantly enriched (*P* < 0.05) KEGG/MitoCarta3.0 pathways/gene sets and Gene Ontology nodes, respectively. (**C**, **D**) From (**A**). (**E**, **F**) Cytoskeletal, sarcomeric (scm), and extracellular matrix (ECM) enriched Gene Ontology Biological Process and Cellular Component nodes from selected parent nodes. Gene Set Enrichment Analysis (GSEA) analyses for (**A**–**F**) were performed on normalised and transformed proteomic and metabolomic datasets using clusterProfiler (version 4.8.1).

To investigate implicated pathways, Gene Set Enrichment Analysis (GSEA) protein enrichment analyses via clusterProfiler were performed on the normalised data using targeted Kyoto Encyclopedia of Genes and Genomes (KEGG) and MitoCarta3.0 databases (Figs. 3A and EV3A; Datasets EV21–EV24). Selected enriched mitochondrial/energetics-associated pathways/gene sets were annotated, ranked, and cross-HF group comparisons were performed (Figs. 3C,D,G and EV3C,D).

Lipid metabolism, atherosclerosis, and cholesterol metabolism pathways were positively enriched in HF. Cholesterol metabolism was specifically enriched in ICM-DM, ICM-No DM, and NICM-DM. This enrichment was largely attributed to the upregulation of intramyocardial chylomicron apolipoproteins, including APOA4, APOB, and APOC3, in all HF conditions, with all but one (APOL6) being upregulated in ICM-DM. There were many common negatively enriched pathways/gene sets such as lipid metabolism, carbohydrate metabolism, thermogenesis, and diabetic cardiomyopathy in all HF groups. Additional pathways/gene sets were negatively enriched only in ICM-DM, such as the citrate cycle, mitochondrial RNA, fatty acid, branched-chain amino acid (BCAA), and lysine metabolism. Another notable negatively enriched metabolic pathway was propanoate metabolism, along with the peroxisome gene set, which were shared with NICM.

Oxidative phosphorylation (OXPHOS) and many of its subdivided gene sets were also observed as negatively enriched in all HF conditions, however, showed greater enrichment in ICM-DM, wherein a greater number of the contained gene/proteins were FDR significant from the differential analysis (90 OXPHOS genes DA in ICM-DM out of 169; NICM-No DM, 35; NICM-DM, 27). All were downregulated except for the upregulation of HIG1 hypoxia inducible domain family member 1A (HIGD1A). CI subunits were most enriched in ICM-DM (enrichment score = −0.85) followed by NICM-DM (enrichment score = −0.76). Other proton pumping complexes and their subunit gene sets; CIII, CIV, and CV, were similarly negatively enriched in ICM-DM wherein CIII and CIV were co-enriched in NICM-DM and CV was ICM-DM specific. Gene sets mediating electron transport for OXPHOS, such as iron–sulfur cluster biosynthesis (enriched in DM) coenzyme Q metabolism (enriched in ICM), and electron carriers were also negatively enriched in ICM-DM.

The clusterProfiler KEGG enriched pathway analysis on the normalised metabolomic MS data identified a positive enrichment in histidine metabolism and a negative enrichment in thyroid hormone signalling pathways exclusive to ICM-DM vs AMD (Figs. 3B and EV3B; Datasets EV25–EV28).

### Extracellular matrix, cytoskeletal, and sarcomeric enriched gene sets

GSEA Gene Ontology (GO) enrichment analysis was also performed on selected cytoskeletal, sarcomeric, and ECM parent nodes from the Biological Process (BP) and Cellular Component (CC) libraries vs AMD (Figs. 3E,F,H and EV3E,F; Datasets EV29–EV32). Cardiomyocyte contraction and conduction-related nodes were seen to be negatively enriched specific to ICM conditions, whereas regulation of cardiac muscle contraction by calcium ion signalling was unique to ICM-DM. This enrichment was largely attributable to the downregulation of cardiac troponin I (TNNI3), sarcoplasmic reticulum Ca2^+^ ATPase (ATP2A2), Na^+^/Ca2^+^ exchanger (SLC8A1), ryanodine receptor (RYR2), and calsequestrin 2 (CASQ2) as determined from the differential analysis. ICM-DM-specific positive enrichment was observed in many actin/cytoskeletal nodes such as Arp2/3 protein complex, podosome, actin filament, and stress fibre nodes, where cytoplasmic microtubule (strongly influenced by SAA1, TPT1, and MTUS2) was negatively enriched. GO nodes only positively enriched in DM included barbed-end actin filament capping, actin cap, and actomyosin structure organisation and actomyosin nodes wherein ICM-DM showed deviation from NICM-DM and No DM in higher myosin heavy chain (MYH10, MYH11, MYH9, and MYL9) FCs. Multiple ECM nodes were positively co-enriched in all HF conditions, but more proteins were observed to be FDR upregulated in ICM-DM from the differential analysis, such as in elastic fibre assembly and collagen fibril organisation, strongly influenced by collagens (COLs), with unique upregulation in ICM-DM in proteins such as FKBP prolyl isomerase 10 (FKBP10) and elastin microfibril interfacer 1 (EMILIN1). We previously reported an increased elastic fibre assembly and elastogenesis within both rat and human ICM (Hume et al, 2023). Cytoskeletal node, actin filament assembly, was also positively co-enriched in HF.

### Ischaemic cardiomyopathy with diabetes and diabetes-specific targeted analyses

Supplementary GO enrichment analyses via PANTHER from significantly DA proteins vs AMD revealed gene sets which were commonly enriched in HF (Fig. EV4A,B; Datasets EV33 and EV34). Analyses also identified ICM-DM-specific impaired mitochondrial metabolism due to the downregulation of alpha-ketoglutarate, pyruvate and acyl-CoA dehydrogenases (ACAD8, ACAD9, ACADVL, GCDH), acetyl-CoA C-acyltransferase (HADHA, HADHB, ACAA1) and 3-methylcrotonyl-CoA carboxylase activity (Fig. EV4A,C; Dataset EV35). Positively enriched ICM-DM-specific gene sets included cadherin binding and galactose metabolic process (GALM, B4GALT1, GALE).

A diabetes-specific targeted analysis showed negatively enriched Z-disc and contractile gene sets enriched due to the downregulation of CASQ2, peripherin (PRPH), nebulette (NEBL), titin-cap (TCAP), and LIM domain-binding proteins LDB3, PDLIM4, and PDLIM5 (Appendix Fig. S2A,B; Dataset EV36). OXPHOS subunits of C1 (NDUFA5, NDUFB10, and NDUFV2), CIV, cytochrome C oxidase subunit 7C (COX7C), coenzyme Q9 (COQ9), and COQ10, were also downregulated specifically in DM.

As the ICM-DM group had a higher BMI (30.8 ± 5.1) than the other conditions, a BMI-average molecule abundance Pearson correlation test was also performed to identify a potentially significant contributory influence to the results from a high BMI/obesity, whereby, no significance was identified (Appendix Fig. S2C–E; Datasets EV37–EV39).

### Mitochondria and oxidative phosphorylation simulated modelling

**Figure 4:**
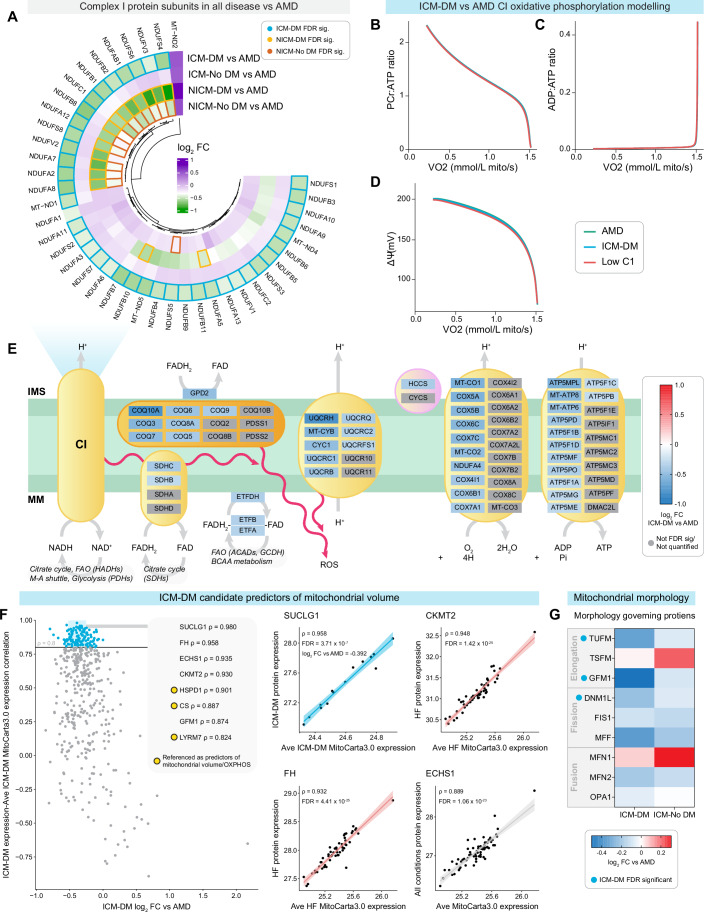
Ischaemic cardiomyopathy with diabetes (ICM-DM) influence on oxidative phosphorylation, mitochondrial composition, and mitochondrial morphology. (**A**) Quantified electron transport chain complex I (CI) subunits from proteomic mass spectrometry (MS) showing the log_2_ fold change (FC) of all heart failure (HF) conditions; ICM-DM, ICM without diabetes (ICM-No DM), non-ischaemic cardiomyopathy with diabetes (NICM-DM), and NICM without diabetes (NICM-No DM), vs age-matched donors (AMD). Significance was determined after Benjamini–Hochberg false discovery rate adjustment (FDR) of *P* values (FDR < 0.05). (**B**–**D**) Simulations of the computational model of oxidative phosphorylation. The groups correspond to AMD myocytes (green; baseline CI activity), ICM-DM myocytes (blue; complex I activity −29.4%, based on proteomic MS) and myocytes with even lower CI expression (red; CI activity −40%). (**D**) The mitochondrial membrane potential (ΔΨ) are plotted against the oxygen consumption rate VO_2_. (**E**) Schematic of oxidative phosphorylation depicting FDR significant proteins, where colour represents log_2_ FC in ICM-DM vs AMD. Proteins not FDR significant or not quantified/analysed represented in grey. CI to CV; left to right. IMS intermembrane space, MM mitochondrial matrix, FAO fatty acid oxidation, ROS reactive oxygen species. (**F**) Log_2_ FC of ICM-DM mitochondrial proteome vs AMD and correlation of ICM-DM individual mitochondrial protein expression to the average relative expression of the ICM-DM mitochondrial proteome across each patient. Only proteins quantified in all samples were analysed. (**G**) Quantified proteins influencing mitochondrial morphology.

To study the potential physiological consequences of reduced CI activity in ICM-DM (Fig. 4A), we employed a biophysical model of OXPHOS (Fig. 4B–D). The model was validated using rat cardiomyocyte OXPHOS data due to its availability over human data. Given the metabolic differences between rat and human cardiac myocytes (Milani-Nejad and Janssen, 2014), the model could not make quantitative predictions but was used to infer trends in physiological variables related to decreased CI activity. The simulations showed that decreased CI activity alone has little effect on the function of OXPHOS, which is consistent with results in prior theoretical studies (Wu et al, 2007) and experimental studies in animals (Lucas and Szweda, 1999; Valsecchi et al, 2012). Figure 4E depicts an intra-mitochondrial schematic representing all DA proteins in ICM-DM vs AMD directly involved in OXPHOS.

Leveraging the opportunity of analysing the pre-mortem human myocardium in healthy and end-stage HF conditions, we sought the protein with which expression per individual best correlated with the combined average expression of the mitochondrial proteome (MitoCarta3.0) as a candidate for predicting mitochondrial volume in ICM-DM, HF, or any condition. Succinate-CoA ligase SUCLG1 was the strongest predictor of ICM-DM mitochondrial volume (ρ = 0.958), while mitochondrial creatine kinase (CKMT2) (ρ = 0.948) and fumarate hydratase (FH) (ρ = 0.932) were best for HF. Enoyl-CoA hydratase ECHS1 (ρ = 0.889) was most suitable across all conditions (Fig. 4F; Datasets EV40–EV42). These were alongside previously referenced proteins as predictors of mitochondrial volume/OXPHOS; citrate synthase (CS) (Kumar et al, 2019; Larsen et al, 2012), heatshock protein HSPD1 (Morgenstern et al, 2021), and LYR motif-containing protein 1 (LYRM1) (McLaughlin et al, 2020). Investigation of proteins regulating mitochondrial morphology identified the downregulation of elongation factors G1 (GFM1) and Tu (TUFM), and fission protein dynamin 1-like (DNM1L) in ICM-DM (Fig. 4G).

### RNA sequencing analysis, and RNA–protein correlation, and co-regulation

**Figure EV5:**
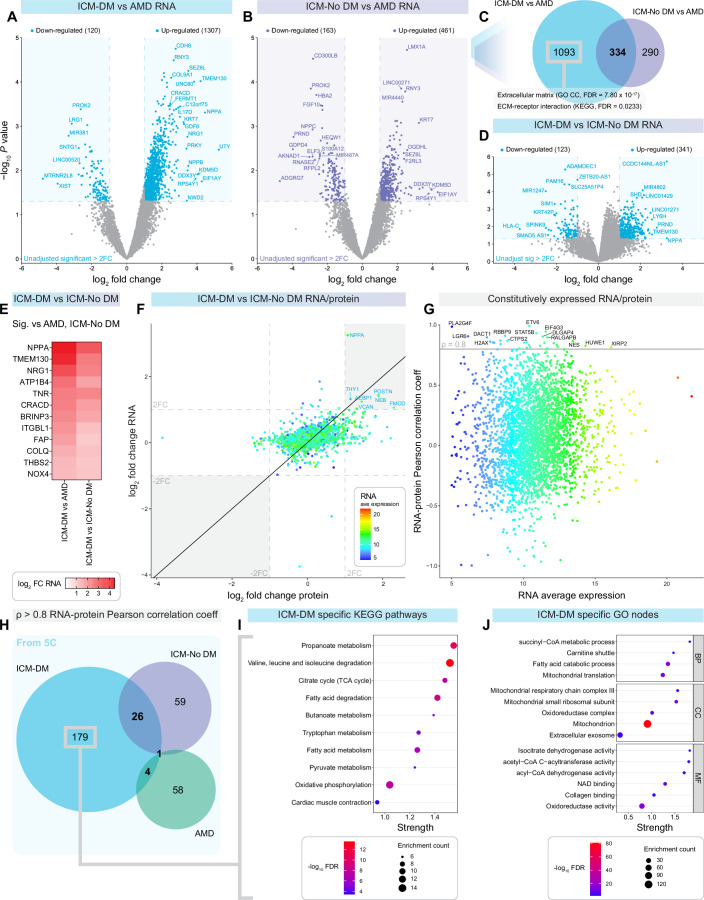
Human left ventricular myocardium RNA sequencing analyses with protein co-expression and correlation in ischaemic cardiomyopathy (ICM) and age-matched donors (AMD). (**A**, **B**) Differential statistical significance was determined as unadjusted significant (*P* = 0.05) and > ±2-fold change (FC). Higher FC significant sequences, written as gene symbols, were annotated. (**A**), ICM with diabetes (ICM-DM) vs AMD. (**B**) ICM without diabetes (ICM-No DM) vs AMD. (**C**) Summary Venn diagram of (**A**, **B**). Sequences which were only significant in ICM-DM were analysed in STRING (https://string-db.org/, version 12.0) which revealed that the Extracellular matrix Gene Ontology (GO) Cellular Component (CC) node and the ECM-receptor interaction KEGG pathway were among the highest significantly enriched nodes/pathways. Nodes/pathways with a Benjamini–Hochberg false discovery rate adjusted *P* value (FDR) < 0.05 were determined as significant. (**D**) ICM-DM vs ICM-No DM RNA. Down and upregulation was defined as significance in ICM-DM compared to ICM-No DM. (**E**) Summary heat map of RNA sequences of biological interest from (**D**) which were both significant in ICM-DM vs AMD and ICM-No DM. (**F**) RNA and protein log_2_ FC plot of ICM-DM vs ICM-No DM where gene symbols which are >2-FC in both protein and RNA are annotated. ICM-DM average RNA expression represented on a colour scale. (**G**) RNA–protein Pearson correlation plot of ICM-DM, ICM-No DM, and AMD combined to identify common constitutively expressed RNA/protein whereby a correlation coefficient of ρ > 0.8 was accepted as being correlated. Highest correlated gene symbols were annotated. Gene symbols were spread out across the x-axis according to average RNA expression of all the groups combined. (**H**) From (**C**), showing gene symbols which had an RNA–protein Pearson correlation coefficient of ρ > 0.8 in ICM-DM, ICM-No DM and AMD. (**I**, **J**) STRING (https://version-11-5.string-db.org/, version 11.5) analysis enrichment bubble plots of selected FDR significant (FDR < 0.05) KEGG pathways and Gene Ontology (GO) nodes from gene symbols which were RNA–protein correlated only in ICM-DM. Strength of the enriched pathways/nodes were calculated as log_10_(observed enrichment count/expected enrichment count from a random set of gene symbols of equal an input set size) wherein the enrichment count (represented as the size of the bubble) was the number of significant differentially expressed gene symbols in that particular node. (**I**) Enriched KEGG pathways. (**J**) Enriched GO Biological Process (BP), CC, and Molecular Function (MF) nodes. Differential analyses for (**A**, **B**, **D**) were performed using a moderated *t* test with the limma package (version 3.56.2) in R (version 4.3.1) following log_2_ transformation. ICM-DM *n* = 7, ICM-No DM *n* = 7, AMD *n* = 7.

To investigate whether significantly DA proteins may have been regulated at the transcription or post-translational level, or to identify differentially expressed noncoding sequences which could regulate protein expression, RNA sequencing (RNA-seq) was performed on selected ICM-DM (*n* = 7), ICM-No DM (*n* = 7) and AMD (*n* = 7) left ventricular myocardial tissues (Dataset EV43). It was found that 120 transcripts were downregulated and 1307 were upregulated in ICM-DM vs AMD. Of these, 334 were differentially expressed in both ICM-DM and ICM-No DM. The 1093 transcripts which were only differentially expressed in ICM-DM showed significant enrichment in the extracellular matrix GO CC node (FDR = 7.80 × 10^−17^) and the ECM-receptor interaction KEGG pathway (FDR = 0.0233) via STRING analysis (Fig. EV5A–C; Datasets EV44 and EV46).

**Figure 5:**
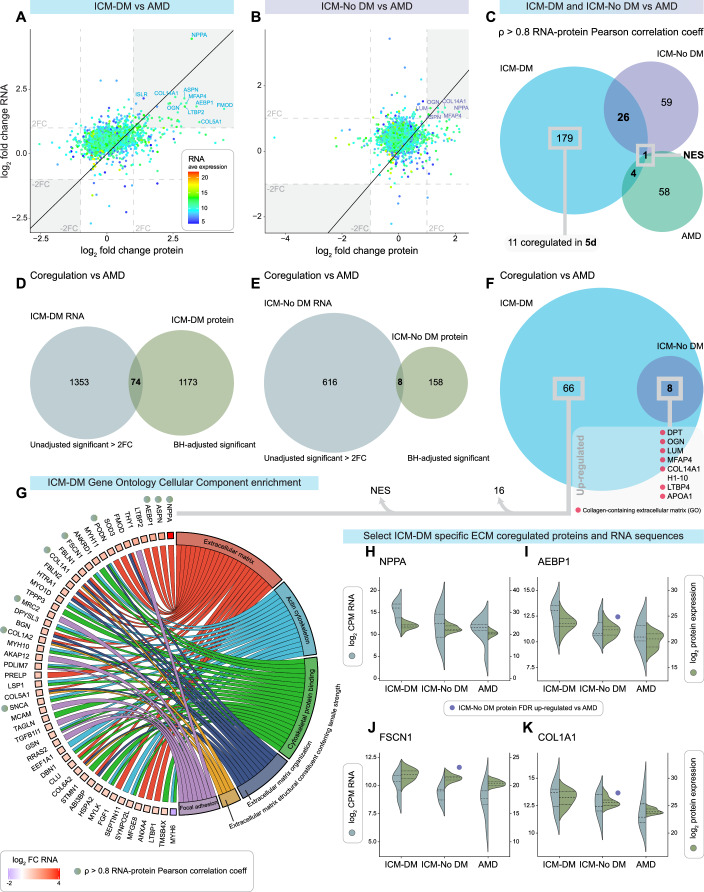
Ischaemic cardiomyopathy with (ICM-DM) and without diabetes (ICM-No DM) RNA–protein correlation and co-regulation vs age-matched donors (AMD). (**A**) RNA and protein log_2_ fold change (FC) plot of ICM-DM vs AMD. ICM-DM average RNA expression represented on a colour scale. (**B**) RNA and protein log_2_ FC plot of ICM-No DM vs AMD. (**C**) Gene symbols which had an RNA–protein Pearson correlation coefficient of ρ > 0.8 in ICM-DM, ICM-No DM and AMD. (**D**) ICM-DM vs AMD significantly differentially expressed RNA (*P* < 0.05 and > ±2-FC) and protein (FDR < 0.05) gene symbols. (**E**) ICM-No DM vs AMD significant RNA and protein gene symbols. (**F**) From (**D**, **E**), including list of gene symbols RNA–protein co-regulated in both ICM-DM and ICM-No DM vs AMD. (**G**) Gene Ontology Cellular Component enrichment chord plot of extracellular, cytoskeletal and focal adhesion related gene symbols from (**F**) which were co-regulated only in ICM-DM vs AMD with RNA log_2_ FC represented. RNA–protein Pearson correlation coefficient of ρ > 0.8 indicated. (**H**–**K**) Selected gene symbols of interest from (**G**) showing log_2_ transformed RNA and protein expression. Purple circles highlight the gene symbols which were also ICM-No DM vs AMD significantly differentially expressed (FDR < 0.05) in the proteomic analysis. ICM-DM *n* = 14 (proteomics) and 7 (RNA-seq), ICM-No DM *n* = 16 (proteomics) and 7 (RNA-seq), AMD *n* = 20 (proteomics) and 7 (RNA-seq).

Paired RNA and protein log_2_ FC of ICM groups were plotted to compare FC similarities compared to AMD (Figs. 5A,B and EV5F). An RNA–protein Pearson correlation analysis was performed in each condition wherein 179 RNA transcripts and proteins were correlated only in ICM-DM, and nestin (NES), a biomarker for mitosis, was the only gene symbol co-correlated in all conditions (Fig. 5C; Datasets EV48–EV50). NES protein was upregulated in all HF conditions, with the greatest FCs in DM, and was RNA–protein co-regulated in ICM-DM.

It was found that 74 gene symbols were RNA–protein co-regulated in ICM-DM vs AMD in the same direction, 66 of which were only observed in ICM, and all ICM-No DM co-regulated gene symbols were shared by ICM-DM (Fig. 5D–F; Dataset EV52). A GO chord plot of selected significantly enriched CC nodes was formed to summarise the RNA and protein co-regulated only in ICM-DM showing that 49 of the 66 gene symbols were contained within extracellular, cytoskeletal, and focal adhesion gene sets (Fig. 5G). Of these, 9 gene symbols were also RNA–protein correlated such as natriuretic peptide A (NPPA), adipocyte enhancer-binding protein 1 (AEBP1), fascin (FSCN1), and COL1A2 (Fig. 5H–K).

A correlation analysis of all groups combined identified proteins likely constitutively expressed at the transcription level irrespective of HF (Fig. EV5G).

Analysed extracellular matrix secretome proteins and RNA have been isolated in Datasets EV72 and EV73 (Uhlén et al, 2019).

### Ischaemic cardiomyopathy with and without diabetes comparison

**Figure EV6:**
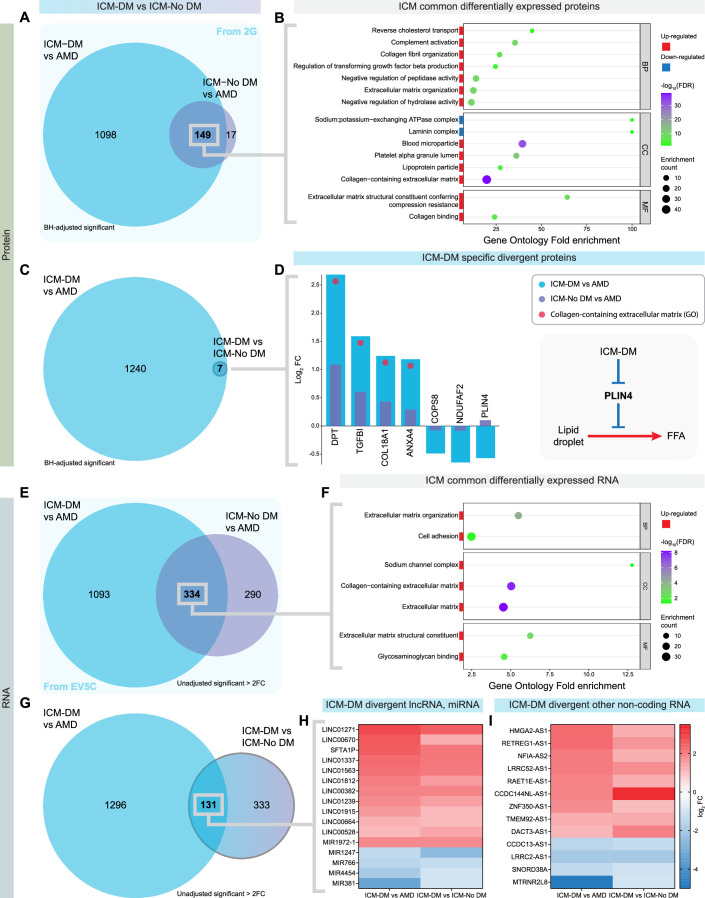
Human ischaemic cardiomyopathy (ICM) common differentially expressed myocardial proteins and RNA, and ICM with diabetes (ICM-DM) specific divergent proteins and RNA. (**A**) From Fig. 2G of ICM-DM and ICM without diabetes (ICM-No DM) vs AMD significant differentially expressed proteins. Statistical significance was determined following Benjamini–Hochberg false discovery rate adjustment (FDR) of *P* values (FDR < 0.05). (**B**) Gene Ontology (GO) analysis by PANTHER (http://geneontology.org/, PANTHER17.0) enrichment bubble plot showing selected significantly enriched Biological Process (BP), Cellular Component (CC), and Molecular Function (MF) nodes from proteins which were significantly differentially expressed in both ICM-DM and ICM-No DM vs AMD. Nodes with an FDR < 0.05 were considered statistically significant. This plot was the combination of two separate GO analyses; one from downregulated proteins compared to AMD (blue) and one from upregulated proteins compared to AMD (red). Enrichment count, represented as the size of the bubble, is the number of significant proteins in that particular node. GO fold enrichment is calculated as observed enrichment count/expected enrichment count from a random set of gene symbols of equal an input set size. (**C**) ICM-DM vs AMD and ICM-DM vs ICM-No DM. (**D**) A log_2_ fold change (FC) bar plot of ICM-DM and ICM-No DM vs AMD of proteins found to be significant in ICM-DM vs ICM-No DM (ICM-DM-specific and divergent from ICM-No DM). PLIN4, which was reduced in ICM-DM vs AMD while increased in ICM-No DM vs AMD, negatively regulates free fatty acid (FFA) availability. (**E**) Summary Venn diagram from Fig. EV5C of ICM-DM and ICM-No DM vs AMD significant differentially expressed RNA. Significance of RNA expression was determined as unadjusted significant (*P* = 0.05) and ±2-FC. (**F**) GO analysis by PANTHER of significant differentially expressed RNA in both ICM-DM and ICM-No DM vs AMD. (**G**) Venn diagram of significantly expressed RNA in ICM-DM vs AMD and ICM-DM vs ICM-No DM. (**H**) Long noncoding and microRNA sequences, and (**I**) other noncoding RNA significant in ICM-DM vs AMD and ICM-DM vs ICM-No DM in the same direction. Scale bar is shared between (**H**) and (**I**).

Further targeted analyses via PANTHER using the differential analysis results investigated the comparison between ICM-DM and ICM-No DM. Commonly downregulated proteins vs AMD revealed negative enrichment of the sodium:potassium-exchanging ATPase complex gene set unique to ICM (Fig. EV6A,B; Dataset EV34). ICM common DA transcripts produced positive enrichment of ECM and cell adhesion gene sets (Fig. EV6E,F; Dataset EV47).

Proteins which were ICM-DM characteristic and divergent from ICM-No DM included upregulation of collagen-containing extracellular matrix-related proteins, dermatopontin (DPT), transforming growth factor β induced (TGFBI), COL18A1, and annexin 4 (ANXA4), and downregulation of COP9 signalosome subunit 8 (COPS8), NDUFAF2, and perilipin 4 (PLIN4) (Fig. EV6C,D). An RNA–protein correlation analysis also identified ICM-DM-specific enriched pathways/gene sets (Fig. EV5H–J; Datasets EV53 and EV54).

Numerous cardiac pathology-associated coding (Fig. EV5D,E), and noncoding sequences such as long noncoding RNAs (lncRNA), microRNAs (miRNA), and antisense RNAs, which are known to regulate gene expression, were also identified to be ICM-DM-specific and divergent (Fig. EV6G–I).

### Sex influence in multi-omic MS analyses

**Figure EV7:**
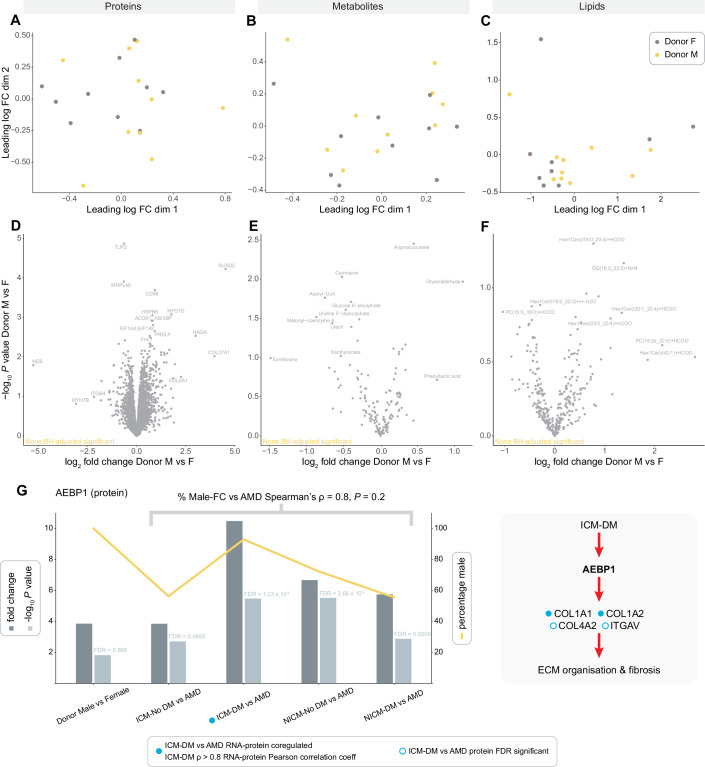
Healthy donor male myocardium vs female and potential male-dominant influence in heart failure. (**A**–**C**) Multidimensional scaling (MDS) plots, a tool to visualise high dimensional data in two dimensions, were used to iteratively separate samples (each point) in distance based on their dissimilarity of paired data (proteins, metabolites, and lipids; dimensions) calculated as the leading log_2_ fold change (FC, average root-mean-square of the largest log_2_ FCs). Healthy donor males represented as yellow, females as grey. (**D**–**F**) Volcano plots of male vs female molecule (proteins, metabolites, and lipids, respectively) abundance whereby significance was determined after Benjamini–Hochberg false discovery rate adjustment (FDR) of *P* values (FDR < 0.05). The was no significance in abundance between donor males and females. Analyses were performed using a moderated *t* test with the limma package (version 3.56.2) in R (version 4.3.1) following log_2_ transformation. AMD males *n* = 10, AMD females *n* = 10 (proteomics and metabolomics) and 9 (lipidomics). (**G**) Bar plot of protein AEBP1 fold change (dark grey) and log_10_*P* value (light grey, with FDR-adjusted value annotated) in donor males vs females, and heart failure conditions vs age-matched donors (AMD). Superimposed line plot shows the associated percentage of males in the subject group; the donor male group are 100% male. AEBP1 was FDR significant differentially expressed in all HF conditions vs AMD but the most in ICM-DM, which also had the highest FC. The ICM-DM group had the highest percentage of males. A male-protein FC vs AMD nonparametric Spearman’s correlation test was performed on the HF conditions to determine a possible significant male relationship to AEBP1 FC. Significance was determined as ρ > 0.8 and *P* < 0.05. AEBP1 has been identified to positively regulate extracellular matrix (ECM) organisation and fibrosis, and was co-regulated in ICM-DM vs AMD in both RNA and protein, and RNA–protein correlated in ICM-DM.

As all ICM-DM patients in the RNA-seq cohort and all but one in the multi-omic analyses were male, donor male vs female comparisons were performed to assess potential sex-based confounding. No significant differential abundance or separation by sex was observed (Fig. EV7A–F; Datasets EV55–EV58). ECM and fibrosis regulator AEBP1 was upregulated in all HF conditions, with the greatest FC and RNA–protein co-regulation in ICM-DM. Although AEBP1’s expression appeared to correlate with male predominance and FC increases in HF vs AMD, it was not significant (Fig. EV7G).

### Histochemistry

**Figure 6:**
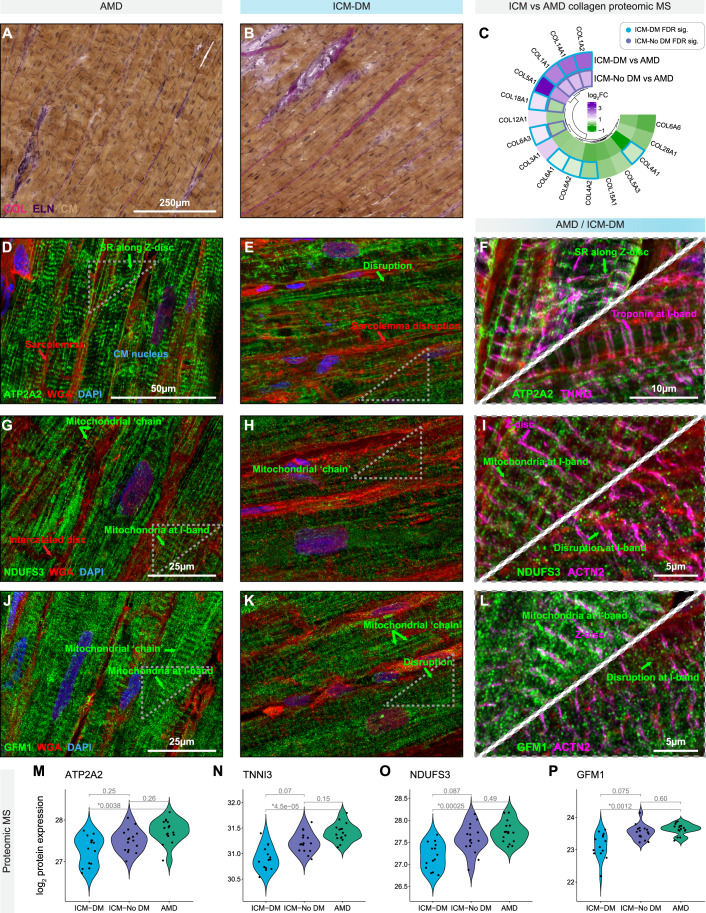
Histochemical visualisation of select key proteins in cryopreserved human left ventricular myocardium of ischaemic cardiomyopathy with diabetes (ICM-DM) and healthy age-matched donor (AMD) tissues. (**A**, **B**) Verhoeff-Van Gieson stain of an AMD and ICM-DM with collagens (COL, magenta), elastin (ELN, purple), cardiomyocytes (CM, brown), and nuclei (blue) (**C**), ICM-DM and ICM-No DM log_2_ fold change (FC) vs AMD, quantified collagen proteins from proteomic mass spectrometry (MS) where Benjamini–Hochberg false discovery rate (FDR) adjusted significantly differentially expressed proteins are indicated. (**D**–**L**) Immunofluorescent confocal microscopy images depicting qualitative differences between AMD and ICM-DM in the labelled protein of interest (green) with a labelled reference protein (magenta). Membranes were stained with fluorophore-conjugated wheat germ agglutinin (WGA, red), and nuclei were stained using DAPI (blue). All images are 4.5-µm-thick Z-stacks, deconvolved using Huygens Professional, and compressed into a two-dimensional image using Fiji/ImageJ Maximum Intensity Projections. (**M**–**P**) Proteomic MS log_2_ transformed quantification of immunohistological proteins of interest, ATP2A2, TNNI3, NDUFS3, and GFM1, in ICM-DM, ICM-No DM, and AMD groups. FDR-adjusted *P* values were (*FDR < 0.05) between groups. Differential analyses for (**M**–**P**) were performed using a moderated *t* test with the limma package (version 3.56.2) in R (version 4.3.1) following log_2_ transformation. ICM-DM *n* = 14, ICM-No DM *n* = 16, AMD *n* = 20.

**Figure EV8:**
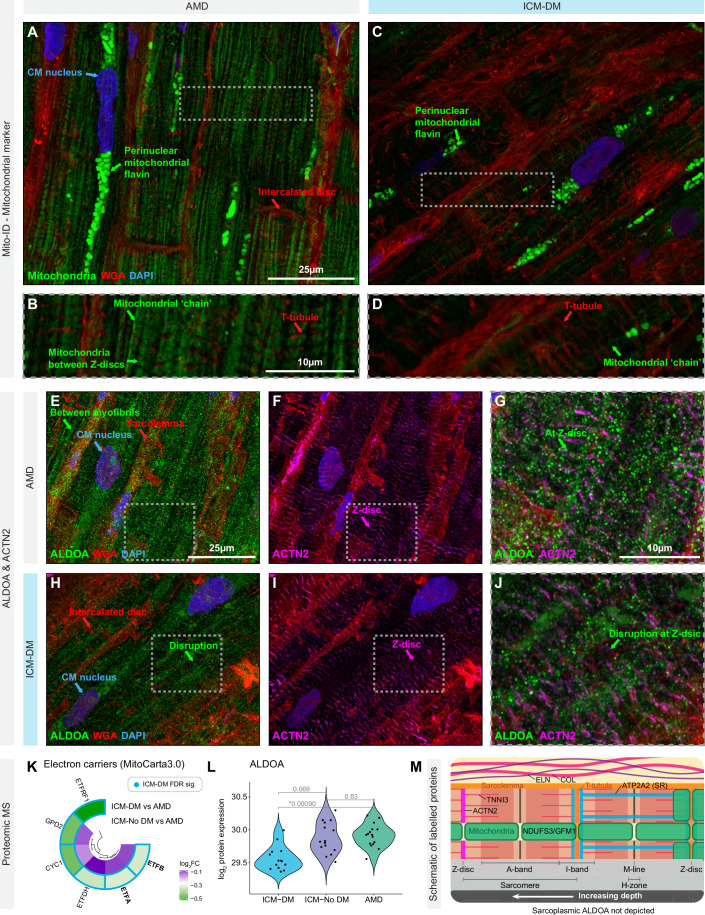
Immunofluorescent labelling of mitochondria and sarcoplasmic ALDOA in cryopreserved ischaemic cardiomyopathy with diabetes (ICM-DM) and healthy age-matched donor (AMD) myocardium. (**A**–**D**) Immunofluorescent confocal microscopy images of cryopreserved human myocardium representing qualitative differences between AMD and ICM-DM in mitochondria labelled with Mito-ID. Bright (autofluorescent) perinuclear mitochondrial flavin identified. (**E**–**J**) Labelled muscle aldolase (ALDOA, green), localised in the sarcoplasm, with a labelled Z-disc protein, ACTN2 (magenta). Membranes, particularly the sarcolemma, were stained with fluorophore-conjugated wheat germ agglutinin (red) and nuclei were stained using DAPI (blue). Cardiomyocytes (CM) are depicted in a longitudinal orientation. All images are 4.5 µm-thick Z-stacks, deconvolved using Huygens Professional, and compressed into a two-dimensional image using Fiji/ImageJ Maximum Intensity Projections. (**K**) Proteomic mass spectrometry (MS) log_2_FC circular heatmap of quantified proteins from the MitoCarta3.0 electron carriers gene set. (**L**) Violin plot of proteomic MS log_2_ transformed quantification of ALDOA in ICM-DM, ICM without diabetes (ICM-No DM), and AMD groups. FDR-adjusted *P* values were annotated to reveal significant differences (*, FDR < 0.05) between groups. Analyses were performed using a moderated *t* test with the limma package (version 3.56.2) in R (version 4.3.1) following log_2_ transformation. ICM-DM *n* = 14, ICM-No DM *n* = 16, AMD *n* = 20. (**M**) Cellular schematic showing localisation of all histologically labelled proteins in this study. Sarcoplasmic ALDOA not depicted. SR sarcoplasmic reticulum. All tissue was pre-mortem. Macroscopic scar tissue, particularly in heart failure conditions, was avoided in all quantitative analyses (mass spectrometry and RNA sequencing) and imaging. Only the most normal/healthy appearing and longitudinally oriented AMD and ICM-DM cardiomyocytes were imaged in confocal microscopy.

Histochemistry was performed to qualitatively observe the myocardial changes in ICM-DM described by proteomic MS. Diffuse interstitial and perivascular collagen fibril staining was observed in ICM vs comparable AMD myocardium, representing increased fibrosis (Fig. 6A–C). Macroscopic infarct/replacement fibrosis was avoided in all analyses (Appendix Fig. S3A,B). Key proteins of interest were labelled and imaged via immunofluorescent confocal microscopy, with cardiomyocytes longitudinally oriented, based on downregulated proteomic MS results and their biological/cited significance. ATP2A2/SERCA2 showed disruption/disorganisation within ICM-DM cardiomyocytes, particularly along the Z-discs, vs AMD, with TNNI3 co-localising along the I-band (Figs. 6D–F,M,N and EV8M; Appendix Fig. S3C,D). Increased interstitial space was noted between the sarcolemma of ICM-DM cardiomyocytes, with broader and disorganised staining of intramembrane glycoproteins indicating ECM linkage dysfunction. Disruption was also indicated in OXPHOS CI subunit NDUFS3 and mitochondrial elongation factor GFM1 proteins at the I-band (Figs. 6G–L,O,P and EV8M; Appendix Fig. S3E,F). Mitochondria were also labelled with Mito-ID where bright aggregates were attributed by the compounding mitochondrial OXPHOS-associated flavin autofluorescence with the highest concentrations in the perinuclear region signifying a notable reduction of both in ICM-DM. Majority of the flavin at this emission wavelength (500–550 nm) was assumed to be in the form of free flavin adenine dinucleotide (FAD), followed by flavin-bound flavoproteins (ETFs) with the least attributed to acyl-CoA dehydrogenases (Fig. EV8A–D,K) (Chorvat et al, 2004; Chorvat et al, 2005; Kunz and Gellerich, 1993; Lu et al, 2014; Romashko et al, 1998; Wust et al, 2015). ICM-DM T-tubules between mitochondria presented disorganisation at Z-disc locations. Glycolytic muscle aldolase (ALDOA), which binds to F-actin of the sarcomere and regulates cardiac hypertrophy (Clarke and Morton, 1976; Li et al, 2018b), was regularly distributed across the Z-disc in AMD but appeared dislocated in ICM-DM (Fig. EV8E–J; Appendix Fig. S3G,H).

## Discussion

We have created a resource on the differential abundance of proteins, metabolites, lipids, and RNA in human left ventricular samples from end-stage ICM-DM compared to AMD and ICM-No DM, and in reference to NICM with and without DM.

### The interactions between fatty acid oxidation and lipolysis

Our results identified the upregulation of circulating chylomicron apolipoproteins (APOs) in ICM-DM, suggesting that diabetes may drive reduced FAO. APOs are positively associated with high blood pressure and atherosclerosis and have been used as an indicator of metabolic syndrome and cardiac stress (Agarwala et al, 2017; Clarke et al, 2023; Gofman et al, 1950; Hunter et al, 2019). The downregulation of components associated with the peroxisome proliferator-activated receptor (PPAR)-α pathway, of long-chain and very-long-chain fatty acid transporters (SLC27A1, CD36), cytosolic fatty acid binding proteins, acyl-CoA synthetases, and proteins involved in FAO, indicates a reduced utilisation of FA in ICM-DM. However, there was an upregulation of albumin (ALB) and transmembrane medium-chain fatty acid transport (LRP1) in addition to a downregulation of lipid droplet-surrounding perilipin (PLIN4) in ICM-DM, which may suggest an increased bioavailability of free fatty acids (FFAs) in the myocardium. Short-chain FAO may be unhindered or even upregulated, as identified in a TAC-induced HF rat model, (Carley et al, 2021) as they do not require transmembrane or intracellular facilitatory transport.

It has been previously identified that ATP synthesis, of which FAO normally accounts for ~50–70% within the healthy myocardium, undergoes a metabolic shift to carbohydrate and ketone reliance in HF (Karwi et al, 2018; Lopaschuk et al, 2021; Stanley et al, 2005). There is evidence that the heart prioritises glucose metabolism over FAO as a source of acetyl-CoA in the presence of oxidative stress or metabolic dysfunction (Bogh et al, 2020; Dyck et al, 2004; Schroeder et al, 2012). Yet, paired with our proteomic results, medium-chain, long-chain, and very-long-chain acylcarnitines were downregulated in HF vs AMD, particularly in ICM-DM, as well as diacylglycerol, an intermediate to triacylglycerol synthesis and lipid droplet formation, suggesting an increased fatty acid metabolism dependence or efflux/loss of fatty acids into the circulation in ICM-DM relative to other HF; used or lost. This concept of lipid efflux, supported by positive myocardial APOA1 RNA–protein co-regulation in ICM, to our knowledge, has not been described in the myocardium but been observed in chick skeletal muscle in response to lipid overload (Shackelford and Lebherz, 1983; Tarugi et al, 1989).

Increased plasma acylcarnitines have been attributed as a characteristic of HF, including ICM-DM, and to identify high-risk patients (Ruiz et al, 2017; Wilshaw et al, 2022; Yoshihisa et al, 2017; Zhao et al, 2020). Our results suggest downregulation of FAO in HF as well as an association with reduced myocardial tissue acylcarntines, but there is a lack of consensus regarding whether FAO is down or upregulated in HF and ICM-DM (Bedi et al, 2016; Fillmore et al, 2014; Karwi et al, 2018; Martín et al, 2000; Stride et al, 2013). There was a downregulation of PLIN4 in ICM-DM only, indicating the initiation of intracellular lipolysis and the release of FFAs for FAO. It has previously been shown that inactivation of PLIN4 results in the downregulation of PLIN5 RNA and protein expression in the heart compared to other organs (Chen et al, 2013; Kien et al, 2022). Reduced PLIN5 has been attributed to exaggeration of the HF phenotype, increased FAO and oxidative stress (Zhou et al, 2019), and has been considered a therapeutic target to prevent myocardial lipid accumulation (Cui et al, 2022).

Myocardial lipogenesis and the resulting formation of lipid droplets have been a well-documented yet poorly understood feature of HF, ischaemia, insulin resistance and diabetes, obesity and metabolic syndrome (Goldberg et al, 2018; Goldenberg et al, 2019; Krahmer et al, 2013; Lee et al, 2013; McGavock et al, 2007; Nyman et al, 2013; Sletten et al, 2018; Wende and Abel, 2010). This condition has been referred to as cardiac steatosis. We identified an overall reduced myocardial lipid content, and to our knowledge, this process has not been investigated in human end-stage ICM-DM.

### Indications of impaired glucose metabolism

The end-stage ICM-DM heart showed signs of promoting glycolysis via intracellular glucose availability, as anticipated in HF and ischaemia due to pathological hypertrophy stimuli and oxidative stress (Dyck et al, 2004; Fillmore et al, 2014; Ritterhoff and Tian, 2023; Shao and Tian, 2016), by the upregulation of glucose trapping hexokinase (HK1, the first rate-limiting step of glycolysis), which was also observed in NICM in agreement with a recent study (Flam et al, 2022).

Diabetes and insulin resistance have been shown to present with hyperglycaemia and impaired insulin-sensitive glucose transporter (SLC2A4/GLUT4) translocation to the plasma membrane and a reduced expression in the rat heart (Leguisamo et al, 2012). Our results also show the downregulation of SLC2A4 in addition to an upregulation of basal glucose transporters (SLC2A1/GLUT1) in ICM-DM with neither DA in other forms of HF. This is in concordance with an early study in dogs (Lei et al, 2004), however, an upregulation of SLC2A1 was identified in rats with coronary ligation-induced HF (Rosenblatt-Velin et al, 2001). As failing and ischaemic hearts have a greater dependence on glycolysis, impaired expression and translocation of SLC2A4 to the sarcolemma due to diabetes could result in an exacerbated reduction in systolic function (Tian and Abel, 2001).

### Reduced expression of ketone and BCAA metabolism proteins

Ketone body, particularly 3-hydroxybutyrate, uptake and metabolism are increased in HF, likely due to FAO being reduced, and has also been proposed as treatment for HF (Bedi et al, 2016; Flam et al, 2022; Voros et al, 2018; Yurista et al, 2021). We found no DA ketone bodies (3-hydroxybutyrate and acetoacetic acid) in the myocardium of end-stage HF patients. Instead, we found protein downregulation of the intracellular monocarboxylate (ketone body) transporter 1 (SLC16A1), 3-hydroxybutyrate dehydrogenase (BDH1), and mitochondrial acetyl-CoA acetyltransferase (ACAT1) in ICM-DM, which are required for ketone metabolism to acetyl-CoA.

Proteins that catalyse BCAA metabolism to acetyl-CoA and succinyl-CoA, an alternative pathway to the citrate cycle, were also downregulated in HF or exclusively in ICM-DM. This suggests downregulation of BCAA metabolism in HF, particularly in ICM-DM, and may explain the upregulation of myocardial BCAAs in HF, as previously indicated in end-stage NICM (Flam et al, 2022) whereby increased tissue BCAA may impair insulin sensitivity (Uddin et al, 2019).

Myocardial Isoleucine leucine (all HF), leucine (ICM and NICM-DM), and valine (ICM) were upregulated in ICM-DM. Increased plasma BCAA concentrations have been found in obese and type 2 DM patients, and DM-induced rodents with cardiomyopathy, and have been suggested to impair insulin-stimulated glucose uptake via the mTORC1 pathway (Lee et al, 2016a; Lynch and Adams, 2014; Newgard et al, 2009; Tai et al, 2010; Tobias et al, 2018; Tremblay et al, 2005). Increased plasma and myocardial BCAAs have also been found in a diabetic cardiomyopathy mouse model with reduced expression of BCAT2, where pyridostigmine induced BCAA metabolism and clearance and attenuated the formation of myocardial scar tissue (Yang et al, 2021).

### Mitochondria, the citrate cycle, and oxidative phosphorylation

Our finding of the downregulation of OXPHOS subunits, particularly CI, in HF and has been reported previously in rodent and human HF and diabetes suggesting a more common HF pathway (Chouchani et al, 2014; Forte et al, 2019; Karamanlidis et al, 2013; Liu et al, 2014; Montaigne et al, 2014; Sebastiao et al, 2022). We found subunits of CI, CIII, CIV, and CV (Fig. 4A,E) with substantially greater significance and FCs in ICM-DM vs AMD relative to the other HF conditions. However, our modelling simulation incorporating CI changes alone demonstrated that reduced FCs are not a rate-limiting factor for sufficient NADH to NAD^+^ conversion/proton gradient, as suggested before (Lucas and Szweda, 1999; Valsecchi et al, 2012; Wu et al, 2007), but a limited capacity may be detrimental under stress (Karamanlidis et al, 2013; Stride et al, 2013). Furthermore, downregulated CI may result in other pathological consequences such as increased oxidative stress, hypertrophy, fibrosis, and glycolysis, and cell death via the mTOR signalling pathway (mTOR pathway proteins exclusively upregulated in ICM-DM) (Balsa et al, 2020; Chouchani et al, 2014; Karamanlidis et al, 2013; Perez-Gomez et al, 2020). Evidence of this effect in our observations was seen in significant upregulation of the NAD^+^/NADH ratio in HF paired with the downregulation of NAD^+^ consumer sirtuin 3 (SIRT3) (Xie et al, 2020). SIRT3 is a regulator of mitochondrial acetyl-CoA and OXPHOS via its deacetylation activity (Ahn et al, 2008; Cimen et al, 2010; Cui et al, 2017; Kane and Sinclair, 2018; Karamanlidis et al, 2013; Koentges et al, 2015; Murugasamy et al, 2022; Palomer et al, 2020; Sundaresan et al, 2016). Our findings are in apparent contrast to previous studies reporting reduced NAD^+^/NADH in HF and in diabetes, mostly in hypertrophy HF rodent models, which may not translate to patients expressing end-stage HF prior to heart transplantation who do not have concentric cardiac hypertrophy but rather a dilated phenotype (Berthiaume et al, 2019; Chiao et al, 2021; Karamanlidis et al, 2013; Lee et al, 2016b). As high NAD^+^/NADH ratios have been coupled with tissue diacylglycerol accumulation via the NADH-dependent actions of glycerol-3-phosphate dehydrogenase, both of which are downregulated in ICM-DM, our findings remain consistent (Fan et al, 2020; Mracek et al, 2013).

OXPHOS was further impaired in ICM-DM by the impairment of FADH_2_ oxidation in three pathways: (1) Oxidation via CII and the citrate cycle; (2) FAO FADH_2_ synthesis by the downregulation of acyl-CoA dehydrogenases; and (3) Oxidation by GPD2 via glycerol metabolism (Alcazar-Fabra et al, 2016; Mracek et al, 2013). This was supported in immunofluorescence microscopy by the apparent reduction of unbound flavins (Chorvat et al, 2004; Chorvat et al, 2005; Kunz and Gellerich, 1993; Romashko et al, 1998; Wust et al, 2015). In addition, CoQ mediates the essential electron transfer from multiple sources (NADH, FADH_2_) between the biochemical actions of CI–CIII and is also a source of reactive oxygen species, while cytochrome C facilitates electron transfer to CIV (Hernansanz-Agustin and Enriquez, 2021). We show CoQ subunits to be downregulated specifically in ICM-DM, while COQ9 was downregulated in DM.

Increased ROS formation from OXPHOS is principally due to pathological reverse electron transfer and it is associated with the metabolic dysfunction of DM (Hernansanz-Agustin and Enriquez, 2021; Kaludercic and Di Lisa, 2020). The gene sets of iron–sulfur clusters, which mediate electron transfer in CI–CIII, were downregulated specifically in DM (Read et al, 2021). Mitochondrial isocitrate dehydrogenase and malic enzyme 3 (ME3), which synthesise mitochondrial NADPH, were found to be downregulated in ICM-DM. Though G6PD was upregulated, it has been shown that there is no direct transport of NADPH from the cytosol to the mitochondria (Niu et al, 2023). In concordance with our results, it has been found that reduction of CI results in reduced mitochondrial NADPH production and increased oxidative stress (Balsa et al, 2020).

Regarding intracellular ATP transport, we show downregulation of the ATP/ADP antiporter, which transports ATP into the cytosol. We also found that kinases which increase ATP availability on demand, catalyse the formation of phosphocreatine, bind ATP, and act as an ATP energy buffer, were downregulated in HF, particularly in ICM-DM, which is consistent with previous studies which also associated downregulation of creatine kinase with a reduced OXPHOS capacity in HF, as well as overall reduced ATP availability (Stride et al, 2013; Weiss et al, 2005).

Mitochondrial dysfunction is not exclusive to diabetes and HF but also represented in other chronic diseases (Diaz-Vegas et al, 2020). Yet, common protein markers used to predict mitochondrial volume/activity have been shown to be inadequate across different organs and in disease (Kumar et al, 2019; Larsen et al, 2012; McLaughlin et al, 2020; Morgenstern et al, 2021). We propose SUCLG1, CKMT2, FH and ECHS1 as potential myocardial mitochondrial protein biomarkers, as indicated by a reduced volume in ICM-DM which was immunobiologically verified by qualitatively reduced mitochondria.

Mitochondria are normally arranged in a regular, repeating pattern between myofibrils and between Z-discs and the cardiomyocyte cytoskeleton (Adams et al, 2023; Kuznetsov et al, 2020; Lyra-Leite et al, 2021; Vendelin et al, 2005). We also observed disorganisation of mitochondria along the I-band, like the studies showing more numerous yet smaller mitochondria with cristae loss in ICM (Chen et al, 2009; Liu et al, 2014) and diabetic (Dabkowski et al, 2009) rodent studies. However, we did not observe upregulation in fission-promoting proteins in HF in concordance with a human diabetes study which also reported no significant correlation between mitochondrial function and BMI (Li et al, 2020a; Montaigne et al, 2014). To our knowledge, there has been no descriptions differentiating ICM with and without DM.

### Compromised critical contractile complexes

Considering the downregulation of critical contractile complexes, we found that cardiac sarcoplasmic reticulum Ca^2+^ handling proteins ATPase and RYR2 were downregulated in ICM-DM only. These have been identified to be reduced in HF previously, particularly the cardiac isoform of ATP2A2/SERCA2 wherein its affinity to Ca^2+^ and its expression were reduced (Abe et al, 2002; Arai et al, 1993; Flesch et al, 1996; Lipskaia et al, 2014; Muslimova et al, 2020; Ohkusa et al, 1997; Studer et al, 1994; Vangheluwe et al, 2007; Vetter et al, 2002). In addition, Na^+^/Ca^2+^ exchanger SLC8A1 (Ca^2+^ efflux) was downregulated specifically in ICM-DM, which contrasts with a study identifying increased left ventricular Na^+^/Ca^2+^ exchanger expression and activity in patients with end-stage HF (Reinecke et al, 1996). SLC8A1 inhibitors have been proposed as a therapeutic target for HF as SAR340835-treated HF dogs showed an improved stroke volume and sympathovagal balance (Pelat et al, 2021). However, the positive effects in acute HF induced in animal models may not translate to chronic human end-stage HF (Pott et al, 2011). We also identified the downregulation of CASQ2 (Ca^2+^ binding) in DM, and the upregulation of PLN, an inhibitory protein of ATP2A2. These changes of poor Ca^2+^ handling impede left ventricular relaxation and influence the rate of contraction in all end-stage HF conditions, but mostly in ICM-DM (Abe et al, 2002; He et al, 1997; Kranias and Hajjar, 2012).

Confocal microscopy revealed disorganisation of ATP2A2 along the I-band of the sarcomere in ICM-DM, co-localising with TNNI3. TNNI3 was downregulated in ICM-DM and NICM, suggesting further contractile impairment which is consistent with its release and elevation in the circulation of patients with advanced HF (Bollen et al, 2017; James et al, 2000; Missov et al, 1997). Furthermore, it has recently been identified that nuclear translocation TNNI3 positively regulates ATP2A2 expression and Ca^2+^ uptake (Lu et al, 2022).

ALDOA has been identified as a critical enzyme promoting glycolysis and as an F-actin binding protein at the I-band and a positive regulator of Ca^2+^ ryanodine receptors (Clarke and Morton, 1976; Kraft et al, 2000; Kramerova et al, 2008; Seo et al, 2006). ALDOA is in low concentrations in post-MI patients’ plasma wherein an increased expression may have a protective role to alleviate oxidative stress and impair cardiomyocyte apoptosis in ischaemic conditions via the VEGF-NOTC1-JAG1 pathway (Luo et al, 2021). However, overexpression of ALDOA has also been attributed to cardiac hypertrophy development (Li et al, 2018b). Our study identifies that ALDOA co-localises at the I-band/Z-disc of human cardiomyocytes and is downregulated and dislocated in ICM-DM, suggesting DM as a specific driver.

### Regulators of the extracellular matrix

The expression of AEBP1, a protein known for its positive regulation of ECM organisation and fibrosis-related proteins, including COL1A1 and COL1A2, which exhibited RNA–protein correlation/co-regulation in ICM-DM (Blackburn et al, 2018), has been demonstrated to increase in response to hyperglycaemia, high fructose, and lipid concentrations in human hepatocytes (Gerhard et al, 2019). The expression of AEBP1 has also been linked to diabetes (Tao et al, 2021). A AEBP1 has also been shown to act as a transcription factor that negatively regulates the gene expression of fatty acid binding protein, FABP4 (Lyons et al, 2006). However, overexpression of AEBP1 in mice on a high-fat diet resulted in increased obesity (Ro et al, 2007; Zhang et al, 2005).

Myocardial upregulation of the ECM and COL deposition via myofibroblasts are common maladaptive feature in ICM and NICM (Fan et al, 2012; Frangogiannis, 2019; Li et al, 2018a; Liu et al, 2017; Travers et al, 2016). We identified ICM-DM upregulation of fibroblast activation protein alpha (FAP) transcript. Fibroblast integrin proteins, which promote ECM adhesion, can interact with POSTN, an ECM scaffolding protein, and stimulate angiogenesis, fibroblast proliferation and differentiation, and COL1 deposition by positively regulating TGFB1 and α-smooth muscle actin (Ashley et al, 2017; Deng et al, 2021; Nan et al, 2024; Qiao et al, 2024; Xu et al, 2021). In addition, dermatopontin (DPT) positively regulates ECM formation, including fibronectin and COL deposition and fibrillogenesis, which may promote maladaptive reverse remodelling in ICM and NICM (Lu et al, 2021; Okamoto and Fujiwara, 2006; Zhao et al, 2022). Our study found DPT was upregulated in all HF conditions with a greater than two-fold increase in ICM-DM relative to other HF conditions. This aligns with the previous finding of DPT and fibrosis upregulation in adipocytes of individuals with obesity and obesity-associated type 2 DM (Unamuno et al, 2020).

ECM secretory proteins fibulin 5 (FBLN5) and microfibril-associated protein 4 (MFAP4, RNA–protein-co-regulated) were upregulated in all HF conditions, most in ICM-DM, and were recently identified as HF-associated serum proteins independent of traditional risk factors suggesting plasma biomarkers of left ventricular origin (Dataset EV74) (Shah et al, 2024; Uhlén et al, 2019).

Taken together, we hypothesise a pathological feedback loop in the ICM-DM myocardium. ICM induced mitochondrial stress and an increase in ROS, promoting lipogenesis may inhibit carnitine transport into the mitochondria and FAO, while also promoting the breakdown of lipid droplets in response to energy deprivation and the inhibition of adaptive glucose influx because of diabetes. The resulting intracellular lipid overload may contribute to impaired cardiac contraction, induced fibrosis, and an efflux of fatty acids from the cardiomyocytes. Further energy deprivation may be possible due to the apparent downregulation of glycolysis, ketone and amino acid metabolism, and dysfunction of mitochondria.

### Limitations

This study is not without limitations. Efforts were made to control variables between conditions (age, macroscopic scar tissue, etc) to limit false results, including separate BMI and sex analyses where control was not possible. Acquisition and comparison of non-cardiomyopathy myocardium with diabetes was not possible. Not all proteins were quantified in this study, particularly low-abundance proteins, which may also have resulted in a lower RNA–protein correlation.

### Conclusion

To our knowledge, this is the most comprehensive multi-omics resource on the molecular influence of DM in human end-stage ICM and NICM to date. Our results indicate that the cardiometabolic dysfunction of HF is further confounded and exacerbated in the presence of diabetes.

## Methods

**Table:** Reagents and tools table

Reagent/resource	Reference or source	Identifier or catalogue number
**Experimental models**		
Human left ventricular myocardium	This study	N/A
**Antibodies**		
Alexa Flour 647-conjugated wheat germ agglutinin	Molecular Probes	W32466
Alexa Fluor 488 donkey anti-rabbit IgG	Thermo Fisher Scientific	ab15065
Anti-ACTN2 mouse IgG	Sigma-Aldrich	A7732
Anti-ALDOA rabbit IgG	Sigma-Aldrich	HPA004177
Anti-ATP2A2/SERCA2 mouse IgG	Thermo Fisher Scientific	MA3919
Anti-GFM1 rabbit IgG	Sigma-Aldrich	HPA061405
Anti-NDUFS3 rabbit IgG	Abcam	ab177471
Anti-TNNI3 (cardiac troponin I) rabbit IgG	Abcam	ab47003
DyLight 550 IgG donkey anti-mouse IgG	Abcam	ab9879
**Chemicals, enzymes, and other reagents**		
Acetonitrile (HPLC grade)	Thermo Fisher Scientific	A955-4
Ammonium formate	Sigma-Aldrich	70221
Chloroacetamide	Sigma-Aldrich	C0267
Chloroform (HPLC grade)	Thermo Fisher Scientific	C606SK-1
DAPI	Thermo Fisher Scientific	62248
DPX	Sigma-Aldrich	06522
Formic acid (LC-MS grade)	Thermo Fisher Scientific	A117-50
Methanol (HPLC grade)	Thermo Fisher Scientific	A456-4
Mito-ID	Enzo Life Sciences	ENZ-51022-0100
Pierce™ BCA Protein Assay	Thermo Fisher Scientific	23225
ProLong Diamond antifade mountant	Thermo Fisher Scientific	P36970
Qubit™ dsDNA High Sensitivity Quantification Assay Kit	Thermo Fisher Scientific	Q32851
Sodium deoxycholate	Sigma-Aldrich	30970
Superfrost Plus slides	Menzel, Thermo Fisher Scientific	J1810AMNZ
TCEP	Thermo Fisher Scientific	20490
Tris-HCl pH 7.5	Sigma-Aldrich	T5941
Triton^™^ X-100	Sigma-Aldrich	T8787
TRIzol	Invitrogen	15596026
Trypsin (sequencing grade)	Promega	V5111
Van Gieson’s stain	This study	N/A
Verhoeff’s Iron Haematoxylin solution	This study	N/A
**Software**		
Adobe Illustrator	Adobe	Version 27.8
BioRender	https://biorender.com	N/A
clusterProfiler	https://bioconductor.org/packages/clusterProfiler	Version 4.8.1
DeepVenn	https://www.deepvenn.com/	N/A
DIA-NN	https://github.com/vdemichev/DiaNN	Version 1.7
Gene Ontology (GO)	https://geneontology.org/	Version 3.17.0
ggplot2	https://github.com/tidyverse/ggplot2	Version 3.4.2
GraphPad Prism	GraphPad	Version 9.5.1
Huygens Professional	Scientific Volume Imaging	Version 22.10.0p1
ImageJ	ImageJ	Version 1.54f
KEGG	https://www.genome.jp/kegg/	Version 102.0
KEGGREST	https://github.com/Bioconductor/KEGGREST	Version 1.40.0
limma	https://github.com/gangwug/limma	Version 3.56.2
LipidSearch	Thermo Fisher Scientific	Version 4.2
Microsoft Excel	Microsoft	Version 2309
MitoCarta3.0	Rath et al, (2021)	N/A
NIS-elements	Nikon	Version 4.0
PANTHER	http://www.pantherdb.org	Version 17.0
R	R	Version 4.3.1
Rsubread	https://bioconductor.org/packages/release/bioc/html/Rsubread.html	Version 2.2.1
SCIEX OS	AB Sciex	Version 1.7.0.36606
scMerge	https://github.com/SydneyBioX/scMerge	Version 1.16.0
SRplot	https://www.bioinformatics.com.cn/en	N/A
STRING	https://string-db.org	Version 12.0
ZEN Blue	Zen	Version 3.3
**Other**		
Agilent 1260 Infinity LC system	Agilent Technologies	N/A
Agilent High Sensitivity D1000 Tapestation system	Agilent	N/A
Atlantis® HILIC column	Waters	N/A
Axio Scan.Z1	Zeiss	N/A
Bioanalyzer	Agilent	N/A
C18 analytical column (1.9 μm)	Dr. Maisch GmbH	N/A
C18 HPLC column	Thermo Fisher Scientific	N/A
Cryotome FSE	Thermo Fisher Scientific	N/A
LabChip GX Touch	Revvity	N/A
NanoDrop	Thermo Fisher Scientific	N/A
Nikon C2plus confocal microscope	Nikon	N/A
NovaSeq 6000	Illumina	N/A
Q-Exactive Fusion Lumos MS	Thermo Fisher Scientific	N/A
Q-Exactive HF-X MS	Thermo Fisher Scientific	N/A
QTRAP5500 Mass Spectrometer	AB Sciex	N/A
Savant SC210 SpeedVac	Thermo Fisher Scientific	N/A
Shiny App (Human-Heart-ALL)	The University of Sydney	http://shiny.maths.usyd.edu.au/Human-Heart-ALL/
SpeedVac SPD120	Thermo Fisher Scientific	N/A
ThermoMixerC	Eppendorf	N/A
TissueLyser LT	Qiagen	N/A
XBridge™ Amide column	Waters	N/A

### Methods and protocols

#### Acquisition of human myocardium

Pre-mortem human myocardium was acquired from the outer anterior and lateral walls of the left ventricle from explanted hearts of end-stage HF (ICM-DM, ICM-No DM, NICM-DM, and NICM-No DM) patients undergoing heart transplantation surgery, and from healthy/non-pathological donors whose hearts could not be viably used for heart transplantation due to logistical and compatibility limitations at the time. Procurement was performed at St Vincent’s Hospital Sydney as previously described (Cao et al, 2019; Crossman et al, 2017; Lal et al, 2015; Lal et al, 2016; Lange et al, 2016; Li et al, 2020b; Mamidi et al, 2017; Mollova et al, 2013; Polizzotti et al, 2015; Sequeira et al, 2015; van Heesch et al, 2019). Hearts were perfused in cardioplegia, placed on wet ice, and myocardial samples were immediately snap-frozen on-site in liquid nitrogen (−196 °C) within 40 min after aortic cross-clamp such that the tissue was not post-mortem and that high-quality RNA was preserved (Lal et al, 2015; Lal et al, 2016; van Heesch et al, 2019). Samples were then stored long-term at −192 °C at the Sydney Heart Bank. Visible myofibrotic scar tissue, such as infarct-affected regions, were avoided. Pathology (HF) of myocardial tissue was histologically determined by hospital anatomical pathology. Diabetic patients were using Metformin and insulin and there were no significant differences in their diabetic care. Informed consent was obtained before the collection of all tissue. All human myocardial donors were Caucasian. This was not by selection, but rather due to tissue availability. The methods of procurement, storage, and use of donated human myocardium were approved by the Human Research Ethics Committee at The University of Sydney (USYD 2021/122). Experiments conformed to the principles set out in the WMA Declaration of Helsinki and the Department of Health and Human Services Belmont Report.

Dissemination of individual-level patient data is not permissible beyond condition/phenotype, age, sex, diabetes/no diabetes, and BMI. However, clinical and demographic data for each quantitative method has been provided in aggregate form (Datasets EV1, EV43, and EV55). Clinical information was not available for every individual. Patient de-identified study IDs and the experiments each individual contributed to is available in Dataset EV59.

#### Proteomics

Cryopreserved human myocardium fragments, lacking macroscopic myofibrotic scar tissue, were crushed and powderised with a mortar and pestle at −196 °C, and weighed to ~10 mg. The powder was suspended in 4% sodium deoxycholate and 100 mM Tris-HCl pH 7.5 and lysed in the ThermoMixerC (Eppendorf) at 95 °C for 10 min. The homogenate was spun at 14,000 × *g* for 10 min and the supernatant collected. Stock protein concentration was quantified by performing a bicinchoninic acid (BCA, Pierce) assay and concentrations were corrected to 0.4 ng/µL. Protein was reduced in 10 nM TCEP and 40 nM chloroacetamide and denatured at 95 °C for 10 min. A trypsin digest was then performed overnight at 37 °C. Peptides were prepared for mass spectrometry as described previously (Harney et al, 2019). A pooled sample was formed and was separated offline with high-pH RP fractionation. An acquity UPLC M-Class CSH C18 130 Å pore size, 300 µm × 150 mm column, and 1.7 µm internal diameter (Waters) was used to fractionate peptides. Separation was performed over 30 min, concatenating 96 fractions into 16 wells using buffer A; 2% acetonitrile and 10 mM ammonium formate (pH 9), and buffer B; 80% acetonitrile and 10 mM ammonium formate (pH 9). Fractionates were dried and resuspended in 5% formic acid. Peptides were directly injected into a C18 (Dr. Maisch, Ammerbuch, Germany, 1.9 µm) 30 cm × 70 cm column with a 10 µm pulled tip integrated online to a nanospray ESI source. Separation involved buffer A; 0.1% formic acid in LC-MS grade water, and buffer B; 80% acetonitrile and 0.1% formic acid in MS grade water. Peptides were differentiated from a gradient of 5–40% of buffer B over 2 h with a flow rate of 300 nL per minute and were ionised by electrospray ionisation at 2.3 kV. A Q-Exactive Fusion Lumos mass spectrometer (Thermo Fisher Scientific) was used to conduct MS/MS analysis with 27% normalised HCD collision energy for fragmentation at the Sydney Mass Spectrometry facility, the University of Sydney. Spectra were procured in a data-independent acquisition using 20 variable isolation windows. RAW data files including the high-pH fractions were analysed using the integrated quantitative proteomics software DIA-NN (Demichev et al, 2020) (version 1.7). The database provided to the search engine for identification contained the Uniprot human database downloaded on the 5th of May, 2020. FDR was set to 1% of precursor ions. Both remove likely interferences and match between runs were enabled. Trypsin was set as the digestion enzyme with a maximum of 2 missed cleavages. Carbamidomethylation of Cys was set as a fixed modification and oxidation of Met was set as variable modifications. Retention time-dependent profiling was used, and the quantification setting was set to any LC (high accuracy). Protein inference was based on genes. The neutral network classifier was set to double-pass mode. The MaxLFQ algorithm was used for label-free quantitation, integrated into the DIA-NN environment (Cox and Mann, 2008; Cox et al, 2011).

Normalised and log_2_ transformed proteomic data per individual is available in Dataset EV60.

#### Metabolomics

Cryopreserved human myocardium was powdered as described in the proteomic MS methods and weighed to 50 mg. Cells were lysed and homogenised with steel balls in methanol/chloroform (2:1; v/v, HPLC grade) using the TissueLyser LT (Qiagen) and kept on dry ice between cycles (3–5 ×1 min). Equal volumes of chloroform then water (HPLC grade) were added to promote protein precipitation. Protein and debris were pelleted at 14,000 rpm at 4 °C for 20 min and the metabolite-containing supernatant was collected. The SpeedVac SPD120 (Thermo Fisher Scientific) was used to dry the solution under nitrogen steam. Compounds were resuspended in the acetonitrile/methanol/formic acid (75:25:0.2; v/v/v, HPLC grade; Thermo Fisher Scientific) for the HILIC analysis, and acetonitrile/methanol (25:25; v/v/v, HPLC grade) for the AMIDE analysis. Targeted metabolite profiling was performed using deuterated internal standards to determine mass spectrometry (MS) multiple reaction-monitoring transitions, declustering potentials, collision energies and chromatographic retention time, as described previously (Koay et al, 2021a; Koay et al, 2021b). HILIC and AMIDE mass spectrometry analyses were performed via liquid chromatography-tandem mass spectrometry (LC-MS/MS) using an Agilent 1260 Infinity liquid chromatography (Santa Clara, CA, USA) system coupled to a QTRAP5500 mass spectrometer (AB Sciex, Foster City, CA, USA) at the Sydney Mass Spectrometry facility, the University of Sydney. Positive and negative ion modes were used to separate polar compounds in hydrophilic interaction liquid chromatography (HILIC) mode using an Atlantis® HILIC column (Waters), and an XBridgeTM Amide column (Waters), respectively, which allow the separation of metabolites of different properties, as previously described (Koay et al, 2021a; Koay et al, 2021b). Samples were randomised across three sequential batches. Pooled metabolite extracts formed from each sample were included after every 10 study samples in the sample queue to detect temporal dips in instrument performance following the analysis. SCIEX OS (AB SCIEX, version 1.7.0.36606) was later used for multiple reaction-monitoring Q1/Q3 peak integration of the raw data files whereby the abundance was quantified as the peak area of a metabolite. QC was performed removing metabolites with poor quantification or to select between metabolites measured in both negative and positive modes. Metabolite abundances were normalised in R (version 4.3.1.) using scMerge (version 1.16.0) (Lin et al, 2019).

Normalised and log_2_ transformed metabolomic data per individual is available in Dataset EV61.

#### Lipidomics

Cryopreserved human myocardium was powdered as described in the proteomic MS methods and weighed to 20 mg. Internal standards were added to each sample in 250 µL methanol (MS grade) containing 0.01% (w/v) butylhydroxytoluene (BHT); 2 nmoles PC(19:0/19:0); 1 nmole each of SM(d18:1/12:0), GluCer(d18:1/12:0), Cer(d18:1/17:0), PS(17:0/17:0), PE(17:0/17:0), PA(17:0/17:0), PI(d7-18:1/15:0), PG(17:0/17:0), CL(14:0/14:0/14:0/14:0), and TG(17:0/17:0/17:0); 0.5 nmoles each of DG(d7-18:1/15:0), CholE(17:0), LPC (17:0), LPE(17:1), and AcCa(d3-16:0); and 0.2 nmole each of Sph(d17:1), S1P(d17:1), LacCer(d18:1/12:0), and MG(d7-18:1). Samples were then homogenised using steel balls in the TissueLyser LT (Qiagen) and kept on wet ice between cycles (3–5 ×1 min). Methyl-tert-butyl-ether (MTBE, 1 mL) was added to the homogenate and samples sonicated at 4 °C for 30 min. Phase separation was induced by adding 250 µL of water (MS grade), vortexing and spinning at 2000 × *g* for 5 min. The upper organic phase was extracted in 5-mL glass tubes. Extraction was performed twice more; sonicated at 4 °C for 30 min in 500 µL MTBE and 150 µL methanol, then vortexed and spun at 2000 × *g* for 5 min after adding 150 µL water. Lipids were dried under vacuum in a Savant SC210 SpeedVac (Thermo Fisher Scientific) and reconstituted in 400 µL 80% methanol/20% water/0.1% formic acid containing 0.01% (w/v) BHT. Lipids were then analysed via liquid chromatography-tandem mass spectrometry (LC-MS/MS) at the Sydney Mass Spectrometry facility, the University of Sydney. LC-MS/MS was performed using a Q-Exactive HF-X mass spectrometer (Thermo Fisher Scientific) coupled to a Vanquish HPLC with a 2.1 × 100 mm C18 HPLC column (Waters, 1.7-µm pore size) as previously described (Couttas et al, 2020) with amendments in the following. HPLC solvent A was 10 mM ammonium formate, 0.1% formic acid in acetonitrile:water (60:40), and solvent B was 10 mM ammonium formate, 0.1% formic acid in isopropanol:acetonitrile (90:10). A 27 min binary gradient at 0.28 mL/min was used: 0 min, 80:20 A/B; 3 min, 80:20 A/B; 5.5 min, 55:45 A/B; 8 min, 36:65 A/B; 13 min, 15:85 A/B; 14 min, 0:100 A/B; 20 min, 0:100 A/B; 20.2 min, 70:30 A/B; 27 min, 70:30 A/B. Data were acquired in full scan/data-dependent MS2 mode (full scan resolution 60,000 FWHM, scan range 220–1600 *m/z*). The sample order was randomised, and data was collected in both positive and negative mode for each sample. The ten most abundant ions in each cycle were subjected to MS2, with an isolation window of 1.4 *m/z*, collision energy 30 eV, resolution 17,500 FWHM, maximum integration time 110 ms and dynamic exclusion window 10 s. A solvent blank was used to created an exclusion list of background ions while an inclusion list was used for all internal standards. LipidSearch software (version 4.2, Thermo Fisher Scientific) was used for lipid annotation, chromatogram alignment, and peak integration. Lipid annotation required both accurate precursor ion mass (tolerance 5 ppm) and diagnostic product ions (tolerance 8 ppm). Individual lipids were expressed as ratios to the class-specific internal standard, then multiplied by the amount of internal standard to calculate molar amounts for each lipid. Lipid levels were expressed as nmoles/mg tissue.

Normalised and log_2_ transformed metabolomic data per individual is available in Dataset EV62. Lipid class names are available in Dataset EV63.

#### RNA sequencing

Cryopreserved human myocardium was powdered as described in the proteomic MS methods and weighed to ~30 mg. Powderised myocardium was lysed and homogenised in 500 µL TRIzol (Invitrogen) using steel balls in the TissueLyser LT (Qiagen) and kept on dry ice between cycles (3–5 ×1 min). In all, 1-bromo-3-chloropopane (50 µL) was added to each sample and left to incubate for 5 min at RT, then spun at 14,000 × *g* at 4 °C for 15 min to induce phase separation. RNA-containing aqueous phase was transferred to a sterile tube and an equal volume of isopropanol was added, mixed by inverting, and left for 1 h at RT. RNA was pelleted at 14,000 × *g* at 18 °C for 15 min, supernatant discarded. RNA was then washed in 4 °C 70% ethanol twice discarding supernatant after spinning at 14,000 × *g* at 4 °C for 10 min and left to dry at RT. RNA was DNase-treated for 30 min at 37 °C and washed again. Pelleted RNA was reconstituted in 15 µL 4 °C nuclease-free diethyl pyrocarbonate (DEPC). Concentration and quality were tested on NanoDrop (Thermo Fisher Scientific) to a standard of 260 nm/280 nm =  1.8–2.0 and 260 nm/230 nm 1.7–2.2. RNA integrity was assessed using an RNA Nano Chip on an Bioanalyzer (Agilent). RNA-seq libraries were prepared with Illumina Stranded Total RNA prep Ligation with Ribo Zero Plus (100 ng input and 11 PCR cycles) according to the manufacturer’s instructions. Quality checks were performed using the Qubit dsDNA High Sensitivity Assay Kit (Thermo Fisher Scientific) and the LabChip GX Touch (Revvity). The final sequencing pool was quantified using the Qubit dsDNA High Sensitivity Assay Kit after pooling all libraries equimolar into a single library pool. Sizing was checked using the Agilent High Sensitivity D1000 Tapestation system. The RNA-seq libraries were sequenced using a paired-end 250 bp kit on a S4 flow cell of the NovaSeq 6000 (Illumina) with a final run concentration of 58 pM and 1% PhiX. The raw data was demultiplexed using bcl2fastq. Library preparation and sequencing were performed by the Ramaciotti Centre for Genomics, at the University of New South Wales, Australia. The quality of each RNA sequence (reversely stranded) was assessed using ShortRead, and the alignment was performed on paired-end sequences without trimming (Liao et al, 2019; Morgan et al, 2009). The alignment of raw sequences was performed on hg38 genome assembly (UCSC) with Rsubread (version 2.2.1) (Liao et al, 2019; Pan et al, 2019). Rsubread was also used for generating the gene count matrix. The gene counts were converted in Count Per Million (CPM), log_2_ transformed, and then normalised using voom (Law et al, 2014).

Normalised and log_2_ transformed RNA sequence data per individual is available in Dataset EV64.

#### Statistics

Statistical analyses were performed on anonymised datasets. Samples were randomised during mass spectrometry acquisition and RNA sequencing library preparation to minimise batch effects prior to statistical analysis.

Differential abundance (DA) or differential expression analyses were performed on proteomic, metabolomic, lipidomic, and RNA-seq datasets using a moderated *t* test with the limma package (version 3.56.2) in R (version 4.3.1) following log_2_ transformation. HF conditions (ICM and NICM) were compared with AMDs; Donors aged 47–65 years for ICM, and Donors aged 21–65 years for NICM. DA analyses were also performed for ICM-DM was also compared against ICM-No DM, and males against females. Molecules with missing values in more than 25% of samples were excluded. No imputation method was applied. Significant molecules were determined by controlling for False discovery rate at the 5% level (FDR < 0.05) using Benjamini–Hochberg FDR adjustment (FDR < 0.05) and ranked using the topTable function in limma. Differential analyses are located in Datasets EV2–EV20, EV44–EV46, EV52, EV56–EV58.

Correlation between RNA and protein (RNA–protein), BMI and molecule, and individual mitochondrial protein expression and the average MitoCarta3.0 protein expression were calculated using Pearson’s correlation coefficient, whereas the correlation between male and AEBP1 fold change (FC, HF compared to AMD) was calculated using Spearman’s correlation coefficient. Correlation between individual mitochondrial protein expression and the total average expression of the mitochondrial proteome (MitoCarta3.0) were also calculated via Pearson’s correlation coefficient where the mitochondrial proteome was limited to proteins quantified and proteins expressed by all samples. Correlation was determined as ρ > 0.8 (Pearson, exceeded FDR), or as ρ > 0.8 and *P* < 0.05 (Spearman). Correlation analyses are located in Datasets EV37–EV42, EV48–EV51.

Gene Set Enrichment Analysis (GSEA) analyses were performed on normalised and transformed proteomic and metabolomic datasets using clusterProfiler (version 4.8.1) (Wu et al, 2021) in R where significance was determined as *P* < 0.05 (unadjusted). Protein enrichment was performed referencing the databases; Kyoto Encyclopedia of Genes and Genomes (KEGG, accessed 21 June 2022) (Kanehisa, 2019; Kanehisa et al, 2023; Kanehisa and Goto, 2000) via KEGGREST (version 1.40.0), MitoCarta3.0 (Rath et al, 2021), and Gene Ontology (GO, version 3.17.0) Biological process (BP) and Cellular Component (CC) (Aleksander et al, 2023; Ashburner et al, 2000). Selected mitochondrial and energetics-associated KEGG pathways and MitoCarta3.0 gene sets were manually annotated. The Kolmogorov-Smirnov statistic was used to compare the ranks of *P* values of genes in the pathways/gene sets vs the uniform distribution using the “GSEA” function. GSEA GO enrichment results were the child nodes of manually selected cytoskeletal, sarcomeric, and extracellular matrix-associated parent nodes; extracellular matrix organisation (GO:0030198), cardiac muscle cell differentiation (GO:0055007), myofibril assembly (GO:0030239), heart contraction (GO:0060047), cytoskeleton organisation (GO:0007010), extracellular matrix (GO:0031012), contractile fibre (GO:0043292), and cytoskeleton (GO:0005856). Significantly enriched protein pathways/gene sets/nodes were fully represented (NICM) or manually selected (ICM) in bubble plot figures. Metabolite enrichment was performed referencing on the KEGG database. clusterProfiler enrichment analyses are located in Datasets EV21–EV32.

Enrichment analyses of targeted genes were performed following and based on the FDR accepted proteomic and RNA-seq differential abundance analyses results using GO via PANTHER (version 17.0) (Thomas et al, 2022) and STRING (version 12.0) (Szklarczyk et al, 2019; Szklarczyk et al, 2023) where significance was determined as FDR < 0.05. STRING analysis (version 11.5) was also performed on ICM-DM RNA–protein correlated genes. GO via PANTHER analyses referenced BP, CC, and Molecular Function (MF) libraries where inputted data was direction specific (down or upregulated) for each analysis before combining the results. STRING analyses referenced GO (BP, CC, and MF) and KEGG databases. Significantly enriched nodes/pathways were manually selected for the figures. Targeted enrichment analyses are in Datasets EV33-EV36, EV47, EV53, and EV54.

#### Graphics

Figure elements were generated in R using ggplot2 (version 3.4.2), clusterProfiler, GraphPad Prism (version 9.5.1), DeepVenn (Hulsen, 2022), Microsoft Excel (version 2309), Adobe Illustrator (version 27.8), at Biorender (https://app.biorender.com), and SRplot (https://www.bioinformatics.com.cn/en) (Tang et al, 2023). Figure 1A and the Synopsis graphics were created with BioRender.com. Molecules of the circular heat maps were clustered via complete-linkage hierarchical clustering, and Log_2_ FCs were separated via Euclidean distance. RNA–protein scatter plots only plotted genes with a relative RNA expression ≥5. CC nodes of the GO chord plot were manually selected based on enrichment. UpSet plots were produced using FDR significant proteins/metabolites in HF vs AMD from the differential analyses. Elements were modified and formatted using Adobe Illustrator.

#### Verhoeff-Van Gieson histochemistry and bright-field microscopy

Donor and ICM-DM cryopreserved pre-mortem human left ventricular myocardium was sectioned on the Cryotome FSE (Thermo Fisher Scientific) at −16 °C while in tissue freezing medium to 16 µm thickness and collected onto Superfrost Plus slides (Menzel, Thermo Fisher Scientific). Sections were fixed in methanol for 3 min at −20 °C, immersed in tap water. Elastin fibres and nuclei were stained in Verhoeff’s Iron Haematoxylin solution; 2.8% filtered ethanol haematoxylin (w/v), 22% ferric chloride (v/v), 22% strong iodine, for 25 min then differentiated in 2% aqueous ferric chloride for 10 dips/seconds. Iodine was washed out in 95% ethanol for 30 s and collagen was then counterstained in Van Gieson’s stain; 0.1% aqueous acid fuchsin, in 90% saturated picric acid (v/v), for 3 min. Sections were then dehydrated in ascending changes of ethanol, cleared in xylene, and mounted in DPX (Sigma-Aldrich).

Bright-field images (24 bid depth) were captured on ZEN blue (version 3.3) using the Zeiss Axio Scan.Z1 and a ×40 plan-apochromat objective with a numerical aperture (NA) of 0.95 on the Hitachi HV-F202SCL camera with an exposure time of 200 µs at the Sydney Microscopy and Microanalysis facility, the University of Sydney. Images were adjusted (brightness and contrast) to identical ranges and exported in Tag Image File Format (TIFF) using Fiji/ImageJ (Schindelin et al, 2012).

#### Immunohistochemistry and confocal microscopy

Donor and ICM-DM sections were sectioned as described for Verhoeff-Van Gieson staining. Sections were encircled with a PAP pen and fixed in 10% neutral buffered formalin for 15 min then washed from free aldehyde groups in 0.2% glycine (w/v) in phosphate buffered saline (PBS). Slides were washed for 5 min three times in PBS between steps at 40 RPM. Tissue was permeabilised in 0.5% (v/v) Triton X-100 (Sigma-Aldrich) in PBS before non-specific binding sites were blocked in 5% (v/v) normal donkey serum and 5% acetylated bovine serum in PBS for 45 min at room temperature (RT) at 40RPM. Monoclonal primary antibodies (IgG) produced in rabbit labelling TNNI3 (ab47003, ~5 µg/mL, Abcam), NDUFS3 (ab177471, ~20 µg/mL, Abcam), GFM1 (HPA061405, ~16 µg/mL, Sigma-Aldrich), or ALDOA (HPA004177, ~10 µg/mL, Sigma-Aldrich) were applied to the sections in 10% blocking solution overnight at 4 °C at 40 RPM. Rabbit IgG were in-turn labelled with secondary fluorophore-conjugated Alexa Flour 488 IgG (ab15065, 5 µg/mL, abcam) overnight at 4 °C at 40 RPM. Mitochondria were also alternatively stained with Mito-ID (ENZ-51022-0100, 2 µL/mL, Enzo Life Sciences) in PBS for 15 min at RT at 40RPM. Co-labelling was similarly performed in sequence with mouse-produced monoclonal IgG labelling ATP2A2 (MA3919, ~10 µg/mL, Invitrogen) or ACTN2 (A7732, ~20 µg/mL, Sigma-Aldrich) and secondary IgG DyLight 550 (ab9879, 5 µg/mL, Abcam). Plasma/intramembrane glycoproteins were bound with Alexa Flour 647-conjugated wheat germ agglutinin (W32466, 5 µg/mL, Molecular Probes) for 1 h at RT at 40 RPM. Nuclei were stained with DAPI (62248, 1 µg/mL, Thermo Fisher Scientific) for 10 min at RT at 40 RPM. Sections were then mounted in ProLong Diamond antifade mountant (Thermo Fisher Scientific).

Z-stack images (12 bit depth) with a thickness of 4.5 µm and step size of 0.3 µm (16 images) were captured on NIS-elements (version 4.0) using a Nikon C2plus confocal microscope, a ×100 oil-immersed plan-apochromat objective with a NA of 1.45 and image parameters; 1.0 airy unit (AU), 1.3× digital zoom, 1/16 frames/sec, 2× frame-average, and a resolution of 2048 ×2048. Channels were imaged in sequence from long to short excitatory laser wavelengths: 640 nm (emission filter 650–700 nm), 561 nm (emission filter 570–620 nm), 488 nm (emission filter 500–550 nm), and 405 nm (emission filter 417–477 nm). Acquisition parameters, including laser power, gain, and offset were identical between donor and ICM-DM and no-primary IgG controls (ICM-DM). Images were acquired at the Sydney Microscopy and Microanalysis facility, the University of Sydney. All channels were deconvolved with Huygens Professional version 22.10.0p1 64b (Scientific Volume Imaging, The Netherlands, http://svi.nl), using the CMLE algorithm. Stain-specific templates were used between conditions to ensure identical deconvolution parameters (acuity, signal-to-noise ratio, iterations, and background value). Z-stacks were compressed into a two-dimensional image using Maximum Intensity Projections, adjusted (brightness and contrast) to identical ranges and exported in OME- TIFF using Fiji/ImageJ (version 1.54 f) (Schindelin et al, 2012).

#### In-silico modelling

To study the physiological consequences of reduced complex I (CI) activity, we employed a biophysical model of oxidative phosphorylation validated on rat cardiac myocytes (Beard, 2005; Vendelin et al, 2000; Vinnakota and Bassingthwaighte, 2004). We reduced the activity parameter corresponding complex I $({\mu }_{{{{\rm{C}}}}1}^{a})$ according to the equation

$${\mu }_{{{{\rm{C}}}}1}^{a}={\mu }_{{{{\rm{C}}}}1}^{a,0}+{RT}\, {{\mathrm{ln}}}\, {{{\rm{FC}}}},$$

where ${\mu }_{{{{\rm{C}}}}1}^{a,0}$ = −21.01 kJ/mol is the baseline value of the activity parameter, *R* = 8.314 J/K/mol is the ideal gas constant, *T* = 297.5 K is the absolute temperature, and FC is the fold change in CI abundance relative to the baseline abundance. The parameters corresponding to the groups were:

- AMD: Baseline value (FC = 1.0).
- ICM-DM: -29.4% from baseline (FC = 0.706), consistent with fold changes in the proteomic data. The fold change was calculated from the mean of the fold changes (ICM-DM relative to AMD) in the CI subunits present in the proteomic data. CI subunits were defined using the HUGO Gene Nomenclature Committee (HGNC) groups “NADH:ubiquinone oxidoreductase core subunits” (https://www.genenames.org/data/genegroup/#!/group/1149) and “NADH:ubiquinone oxidoreductase supernumerary subunits” (https://www.genenames.org/data/genegroup/#!/group/1150).
- Low C1: -40% from baseline (FC = 0.6). This group was included to study the effects of reducing complex I beyond the fold changes seen in the proteomic data.

The model was simulated for each group by iteratively varying the ATP consumption parameter ${\mu }_{{{{\rm{ATPase}}}}}^{a}$, leading to changes in the oxygen consumption rate VO_2_ = $0.5{J}_{{{{\rm{C}}}}4}$. For each value of ${\mu }_{{{{\rm{ATPase}}}}}^{a}$, the model was simulated for 1000 s to reach steady state.

## Supplementary information

Appendix

Peer Review File

EV Datasets

Expanded View Figures

## Data Availability

Proteomic mass spectrometry raw data is available at ProteomeXchange via PRIDE (Perez-Riverol et al, 2022) with the project accession PXD052878 at http://proteomecentral.proteomexchange.org. Metabolomic mass spectrometry and lipidomic mass spectrometry raw data is available at Metabolomics Workbench (Sud et al, 2016) with the Study IDs ST003274 (metabolomics) and ST003275 (lipidomics) under Project PR002031 at 10.21228/M83529. 10.21228/M83529. Lipidomic mass spectrometry raw data is temporarily available at Metabolomics Workbench with the Study ID ST003275 and Project DOI PR002031 at 10.21228/M83529. RNA sequencing raw data is available at NCBI Gene Expression Omnibus (GEO) (Edgar, 2002) at https://www.ncbi.nlm.nih.gov/geo/query/acc.cgi?acc=GSE263297. Raw, processed, and adjusted confocal and bright-field microscopy images are available at https://zenodo.org/records/15788161. 10.5281/zenodo.15788161 (Hunter, 2025). Code for quantitative analyses is available at https://zenodo.org/records/14048213. 10.5281/zenodo.14048213 (Zhang, 2025). Code for in-silico oxidative phosphorylation modelling is available at https://url.au.m.mimecastprotect.com/s/tHvhCL7EwMfQ2gxYOUBfviyxx2V?domain=github.com. The source data of this paper are collected in the following database record: biostudies:S-SCDT-10_1038-S44321-025-00281-9.

## References

[CR1] Abe T, Ohga Y, Tabayashi N, Kobayashi S, Sakata S, Misawa H, Tsuji T, Kohzuki H, Suga H, Taniguchi S et al (2002) Left ventricular diastolic dysfunction in type 2 diabetes mellitus model rats. Am J Physiol-Heart Circ Physiol 282:H138–H148 11748057 10.1152/ajpheart.2002.282.1.H138

[CR2] Adams RA, Liu Z, Hsieh C, Marko M, Lederer WJ, Jafri MS, Mannella C (2023) Structural analysis of mitochondria in cardiomyocytes: insights into bioenergetics and membrane remodeling. Curr Issues Mol Biol 45:6097–6115 37504301 10.3390/cimb45070385 PMC10378267

[CR3] Agarwala A, Pokharel Y, Saeed A, Sun WS, Virani SS, Nambi V, Ndumele C, Shahar E, Heiss G, Boerwinkle E et al (2017) The association of lipoprotein(a) with incident heart failure hospitalization: atherosclerosis risk in communities study. Atherosclerosis 262:131–137 28554015 10.1016/j.atherosclerosis.2017.05.014 PMC5523851

[CR4] Ahn BH, Kim HS, Song SW, Lee IH, Liu J, Vassilopoulos A, Deng CX, Finkel T (2008) A role for the mitochondrial deacetylase Sirt3 in regulating energy homeostasis. Proc Natl Acad Sci USA 105:14447–14452 18794531 10.1073/pnas.0803790105 PMC2567183

[CR5] Alcazar-Fabra M, Navas P, Brea-Calvo G (2016) Coenzyme Q biosynthesis and its role in the respiratory chain structure. Biochim Biophys Acta-Bioenerg 1857:1073–1078 10.1016/j.bbabio.2016.03.010 26970214

[CR6] Aleksander SA, Balhoff J, Carbon S, Cherry JM, Drabkin HJ, Ebert D, Feuermann M, Gaudet P, Harris NL, Hill DP et al (2023) The Gene Ontology knowledgebase in 2023. Genetics 224:14 10.1093/genetics/iyad031 PMC10158837 36866529

[CR7] Arai M, Alpert NR, Maclennan DH, Barton P, Periasamy M (1993) Alterations in sarcoplasmic-reticulum gene-expression in human heart-failure—a possible mechanism for alterations in systolic and diastolic properties of the failing myocardium. CircRes 72:463–469 10.1161/01.res.72.2.463 8418995

[CR8] Ashburner M, Ball CA, Blake JA, Botstein D, Butler H, Cherry JM, Davis AP, Dolinski K, Dwight SS, Eppig JT et al (2000) Gene Ontology: tool for the unification of biology. Nat Genet 25:25–29 10802651 10.1038/75556 PMC3037419

[CR9] Ashley SL, Wilke CA, Kim KK, Moore BB (2017) Periostin regulates fibrocyte function to promote myofibroblast differentiation and lung fibrosis. Mucosal Immunol 10:341–351 27435108 10.1038/mi.2016.61 PMC5250615

[CR10] Balsa E, Perry EA, Bennett CF, Jedrychowski M, Gygi SP, Doench JG, Puigserver P (2020) Defective NADPH production in mitochondrial disease complex I causes inflammation and cell death. Nat Commun 11:12 32483148 10.1038/s41467-020-16423-1 PMC7264245

[CR11] Beard DA (2005) A biophysical model of the mitochondrial respiratory system and oxidative phosphorylation. PLoS Comput Biol 1:252–264 10.1371/journal.pcbi.0010036 PMC1201326 16163394

[CR12] Bedi KC, Snyder NW, Brandimarto J, Aziz M, Mesaros C, Worth AJ, Wang LL, Javaheri A, Blair IA, Margulies KB et al (2016) Evidence for intramyocardial disruption of lipid metabolism and increased myocardial ketone utilization in advanced human heart failure. Circulation 133:706–716 26819374 10.1161/CIRCULATIONAHA.115.017545 PMC4779339

[CR13] Bell DSH, Goncalves E (2019) Heart failure in the patient with diabetes: epidemiology, aetiology, prognosis, therapy and the effect of glucose‐lowering medications. Diab, Obes Metab 21:1277–1290 10.1111/dom.13652 30724013

[CR14] Berthiaume JM, Kurdys JG, Muntean DM, Rosca MG (2019) Mitochondrial NAD(+)/NADH redox state and diabetic cardiomyopathy. Antioxid Redox Signal 30:375–398 29073779 10.1089/ars.2017.7415 PMC6306679

[CR15] Blackburn PR, Xu Z, Tumelty KE, Zhao RW, Monis WJ, Harris KG, Gass JM, Cousin MA, Boczek NJ, Mitkov MV et al (2018) Bi-allelic alterations in AEBP1 lead to defective collagen assembly and connective tissue structure resulting in a variant of Ehlers-Danlos syndrome. Am J Hum Genet 102:696–705 29606302 10.1016/j.ajhg.2018.02.018 PMC5985336

[CR16] Bogh N, Hansen ESS, Omann C, Lindhardt J, Nielsen PM, Stephenson RS, Laustsen C, Hjortdal VE, Agger P (2020) Increasing carbohydrate oxidation improves contractile reserves and prevents hypertrophy in porcine right heart failure. Sci Rep 10:9 32424129 10.1038/s41598-020-65098-7 PMC7235019

[CR17] Bollen IAE, Schuldt M, Harakalova M, Vink A, Asselbergs FW, Pinto JR, Krüger M, Kuster DWD, van der Velden J (2017) Genotype-specific pathogenic effects in human dilated cardiomyopathy. J Physiol-Lond 595:4677–4693 28436080 10.1113/JP274145 PMC5509872

[CR18] Buja LM, Vander Heide RS (2016) Pathobiology of ischemic heart disease: past, present and future. Cardiovasc Pathol 25:214–220 26897485 10.1016/j.carpath.2016.01.007

[CR19] Cao J, Koay YC, Quek LE, Parker B, Lal S, O’Sullivan JF (2019) Myocardial substrate changes in advanced ischaemic and advanced dilated human heart failure. Eur J Heart Fail 21:1042–1045 31184404 10.1002/ejhf.1479

[CR20] Carley AN, Maurya SK, Fasano M, Wang Y, Selzman CH, Drakos SG, Lewandowski ED (2021) Short-chain fatty acids outpace ketone oxidation in the failing heart. Circulation 143:1797–1808 33601938 10.1161/CIRCULATIONAHA.120.052671 PMC8096711

[CR21] Chen L, Gong QZ, Stice JP, Knowlton AA (2009) Mitochondrial OPA1, apoptosis, and heart failure. Cardiovasc Res 84:91–99 19493956 10.1093/cvr/cvp181 PMC2741347

[CR22] Chen WQ, Chang B, Wu XY, Li L, Sleeman M, Chan L (2013) Inactivation of Plin4 downregulates Plin5 and reduces cardiac lipid accumulation in mice. Am J Physiol-Endocrinol Metab 304:E770–E779 23423172 10.1152/ajpendo.00523.2012 PMC3625749

[CR23] Chiao YA, Chakraborty AD, Light CM, Tian R, Sadoshima J, Shi XJ, Gu HW, Lee CF (2021) NAD(+) redox imbalance in the heart exacerbates diabetic cardiomyopathy. Circ-Heart Fail 14:12 10.1161/CIRCHEARTFAILURE.120.008170 PMC8373812 34374300

[CR24] Chorvat D, Bassien-Capsa V, Cagalinec M, Kirchnerova J, Mateasik A, Comte B, Chorvatova A (2004) Mitochondrial autofluorescence induced by visible light in single rat cardiac myocytes studied by spectrally resolved confocal microscopy. Laser Phys 14:220–230

[CR25] Chorvat D, Kirchnerova J, Cagalinec M, Smolka J, Mateasik A, Chorvatova A (2005) Spectral unmixing of flavin autofluorescence components in cardiac myocytes. Biophys J 89:L55–L57 16227502 10.1529/biophysj.105.073866 PMC1367005

[CR26] Chouchani ET, Methner C, Buonincontri G, Hu CH, Logan A, Sawiak SJ, Murphy MP, Krieg T (2014) Complex I deficiency due to selective loss of Ndufs4 in the mouse heart results in severe hypertrophic cardiomyopathy. PLoS ONE 9:6 10.1371/journal.pone.0094157 PMC3976382 24705922

[CR27] Cimen H, Han MJ, Yang YJ, Tong Q, Koc H, Koc EC (2010) Regulation of succinate dehydrogenase activity by SIRT3 in mammalian mitochondria. Biochemistry 49:304–311 20000467 10.1021/bi901627u PMC2826167

[CR28] Clarke FM, Morton DJ (1976) Aldolase binding to actin-containing-filaments—formation of paracrystals. Biochem J 159:797 1008835 10.1042/bj1590797 PMC1164183

[CR29] Clarke R, Von Ende A, Schmidt LE, Yin XK, Hill M, Hughes AD, Pechlaner R, Willeit J, Kiechl S, Watkins H et al (2023) Apolipoprotein proteomics for residual lipid-related risk in coronary heart disease. CircRes 132:452–464 10.1161/CIRCRESAHA.122.321690 PMC9930889 36691918

[CR30] Couttas TA, Rustam YH, Song HT, Qi YF, Teo JD, Chen JB, Reid GE, Don AS (2020) A novel function of sphingosine kinase 2 in the metabolism of sphinga-4,14-diene lipids. Metabolites 10:19 10.3390/metabo10060236 PMC7344861 32521763

[CR31] Cox J, Mann M (2008) MaxQuant enables high peptide identification rates, individualized p.p.b.-range mass accuracies and proteome-wide protein quantification. Nat Biotechnol 26:1367–1372 19029910 10.1038/nbt.1511

[CR32] Cox J, Neuhauser N, Michalski A, Scheltema RA, Olsen JV, Mann M (2011) Andromeda: a peptide search engine integrated into the MaxQuant environment. J Proteome Res 10:1794–1805 21254760 10.1021/pr101065j

[CR33] Crossman DJ, Shen X, Jullig M, Munro M, Hou YF, Middleditch M, Shrestha D, Li A, Lal S, dos Remedios CG et al (2017) Increased collagen within the transverse tubules in human heart failure. Cardiovasc Res 113:879–891 28444133 10.1093/cvr/cvx055

[CR34] Cubbon RM, Adams B, Rajwani A, Mercer BN, Patel PA, Gherardi G, Gale CP, Batin PD, Ajjan R, Kearney L et al (2013) Diabetes mellitus is associated with adverse prognosis in chronic heart failure of ischaemic and non-ischaemic aetiology. Diab Vasc Dis Res 10:330–336 23349368 10.1177/1479164112471064

[CR35] Cui XN, Wang JW, Zhang Y, Wei JL, Wang Y (2022) Plin5, a new target in diabetic cardiomyopathy. Oxid Med Cell Longev 2022:20 10.1155/2022/2122856 PMC9060988 35509833

[CR36] Cui XX, Li X, Dong SY, Guo YJ, Liu T, Wu YC (2017) SIRT3 deacetylated and increased citrate synthase activity in PD model. Biochem Biophys Res Commun 484:767–773 28161643 10.1016/j.bbrc.2017.01.163

[CR37] Dabkowski ER, Williamson CL, Bukowski VC, Chapman RS, Leonard SS, Peer CJ, Callery PS, Hollander JM (2009) Diabetic cardiomyopathy-associated dysfunction in spatially distinct mitochondrial subpopulations. Am J Physiol-Heart Circ Physiol 296:H359–H369 19060128 10.1152/ajpheart.00467.2008 PMC2643887

[CR38] Demichev V, Messner CB, Vernardis SI, Lilley KS, Ralser M (2020) DIA-NN: neural networks and interference correction enable deep proteome coverage in high throughput. Nat Methods 17:41 31768060 10.1038/s41592-019-0638-x PMC6949130

[CR39] Deng CC, Hu YF, Zhu DH, Cheng Q, Gu JJ, Feng QL, Zhang LX, Xu YP, Wang D, Rong ZL et al (2021) Single-cell RNA-seq reveals fibroblast heterogeneity and increased mesenchymal fibroblasts in human fibrotic skin diseases. Nat Commun 12:16 34140509 10.1038/s41467-021-24110-y PMC8211847

[CR40] Diaz-Vegas A, Sanchez-Aguilera P, Krycer JR, Morales PE, Monsalves-Alvarez M, Cifuentes M, Rothermel BA, Lavandero S (2020) Is mitochondrial dysfunction a common root of noncommunicable chronic diseases? Endocr Rev 41:491–517 10.1210/endrev/bnaa005 PMC7255501 32179913

[CR41] Dyck JRB, Cheng JF, Stanley WC, Barr R, Chandler MP, Brown S, Wallace D, Arrhenius T, Harmon C, Yang G et al (2004) Malonyl coenzyme a decarboxylase inhibition protects the ischemic heart by inhibiting fatty acid oxidation and stimulating glucose oxidation. CircRes 94:E78–E84 10.1161/01.RES.0000129255.19569.8f 15105298

[CR42] Ebong IA, Goff DC, Rodriguez CJ, Chen HY, Sibley CT, Bertoni AG (2013) Association of lipids with incident heart failure among adults with and without diabetes mellitus multiethnic study of atherosclerosis. Circ-Heart Fail 6:371 23529112 10.1161/CIRCHEARTFAILURE.112.000093 PMC3991930

[CR43] Edgar R (2002) Gene Expression Omnibus: NCBI gene expression and hybridization array data repository. Nucleic Acids Res 30:207–210 11752295 10.1093/nar/30.1.207 PMC99122

[CR44] Einarson TR, Acs A, Ludwig C, Panton UH (2018) Prevalence of cardiovascular disease in type 2 diabetes: a systematic literature review of scientific evidence from across the world in 2007–2017. Cardiovasc Diabetol 17:83 29884191 10.1186/s12933-018-0728-6 PMC5994068

[CR45] Elosua R, Sayols-Baixeras S, Lluís-Ganella C, Lucas G (2014) Pathogenesis of coronary artery disease: focus on genetic risk factors and identification of genetic variants. Appl Clin Genet 7:15–32 10.2147/TACG.S35301 PMC3920464 24520200

[CR46] Fan D, Takawale A, Lee J, Kassiri Z (2012) Cardiac fibroblasts, fibrosis and extracellular matrix remodeling in heart disease. Fibrogenes Tissue Repair 5:13 10.1186/1755-1536-5-15 PMC3464725 22943504

[CR47] Fan L, Cacicedo JM, Ido YS (2020) Impaired nicotinamide adenine dinucleotide (NAD(+)) metabolism in diabetes and diabetic tissues: implications for nicotinamide-related compound treatment. J Diab Investig 11:1403–1419 10.1111/jdi.13303 PMC7610120 32428995

[CR48] Fillmore N, Mori J, Lopaschuk GD (2014) Mitochondrial fatty acid oxidation alterations in heart failure, ischaemic heart disease and diabetic cardiomyopathy. Br J Pharmacol 171:2080–2090 24147975 10.1111/bph.12475 PMC3976623

[CR49] Flam E, Jang C, Murashige D, Yang Y, Morley MP, Jung S, Kantner DS, Pepper H, Bedi JKC, Brandimarto J et al (2022) Integrated landscape of cardiac metabolism in end-stage human nonischemic dilated cardiomyopathy. Nat Cardiovasc Res 1:817–829 36776621 10.1038/s44161-022-00117-6 PMC9910091

[CR50] Flesch M, Schwinger RHG, Schnabel P, Schiffer F, vanGelder I, Bavendiek U, Sudkamp M, KuhnRegnier F, Bohm M (1996) Sarcoplasmic reticulum Ca(2+)ATPase and phospholamban mRNA and protein levels in end-stage heart failure due to ischemic or dilated cardiomyopathy. J Mol Med 74:321–332 8862513 10.1007/BF00207509

[CR51] Forte M, Palmerio S, Bianchi F, Volpe M, Rubattu S (2019) Mitochondrial complex I deficiency and cardiovascular diseases: current evidence and future directions. J Mol Med 97:579–591 30863992 10.1007/s00109-019-01771-3

[CR52] Frangogiannis NG (2019) The extracellular matrix in ischemic and nonischemic heart failure. CircRes 125:117–146 10.1161/CIRCRESAHA.119.311148 PMC6588179 31219741

[CR53] Gerhard GS, Hanson A, Wilhelmsen D, Piras IS, Still CD, Chu X, Petrick AT, DiStefano JK (2019) AEBP1 expression increases with severity of fibrosis in NASH and is regulated by glucose, palmitate, and miR-372-3p. PLoS ONE 14:20 10.1371/journal.pone.0219764 PMC6625715 31299062

[CR54] Gofman JW, Lindgren F, Elliott H, Mantz W, Hewitt J, Strisower B, Herring V (1950) The role of lipids and lipoproteins in atherosclerosis. Science 111:166 15403115 10.1126/science.111.2877.166

[CR55] Goldberg IJ, Reue K, Abumrad NA, Bickel PE, Cohen S, Fisher EA, Galis ZS, Granneman JG, Lewandowski ED, Murphy R et al (2018) Deciphering the role of lipid droplets in cardiovascular disease: a report from the 2017 National Heart, Lung, and Blood Institute Workshop. Circulation 138:305–315 30012703 10.1161/CIRCULATIONAHA.118.033704 PMC6056021

[CR56] Goldenberg JR, Carley AN, Ji RP, Zhang XK, Fasano M, Schulze PC, Lewandowski ED (2019) Preservation of acyl coenzyme A attenuates pathological and metabolic cardiac remodeling through selective lipid trafficking. Circulation 139:2765–2777 30909726 10.1161/CIRCULATIONAHA.119.039610 PMC6557671

[CR57] Groenewegen A, Rutten FH, Mosterd A, Hoes AW (2020) Epidemiology of heart failure. Eur J Heart Fail 22:1342–1356 32483830 10.1002/ejhf.1858 PMC7540043

[CR58] Guddeti RR, Matsuo Y, Matsuzawa Y, Aoki T, Lennon RJ, Lerman LO, Kushwaha SS, Lerman A (2014) Ischemic cardiomyopathy is associated with coronary plaque progression and higher event rate in patients after cardiac transplantation. J Am Heart Assoc 3:11 10.1161/JAHA.114.001091 PMC4310404 25095871

[CR59] Harney DJ, Hutchison AT, Su ZD, Hatchwell L, Heilbronn LK, Hocking S, James DE, Larance M (2019) Small-protein enrichment assay enables the rapid, unbiased analysis of over 100 low abundance factors from human plasma. Mol Cell Proteom 18:1899–1915 10.1074/mcp.TIR119.001562 PMC6731089 31308252

[CR60] He HP, Giordano FJ, HilalDandan R, Choi DJ, Rockman HA, McDonough PM, Bluhm WF, Meyer M, Sayen MR, Swanson E et al (1997) Overexpression of the rat sarcoplasmic reticulum Ca2+ ATPase gene in the heart of transgenic mice accelerates calcium transients and cardiac relaxation. J Clin Invest 100:380–389 9218515 10.1172/JCI119544 PMC508201

[CR61] Herman DS, Lam L, Taylor MRG, Wang LB, Teekakirikul P, Christodoulou D, Conner L, DePalma SR, McDonough B, Sparks E et al (2012) Truncations of titin causing dilated cardiomyopathy. N Engl J Med 366:619–628 22335739 10.1056/NEJMoa1110186 PMC3660031

[CR62] Hernansanz-Agustin P, Enriquez JA (2021) Generation of reactive oxygen species by mitochondria. Antioxidants 10:18 10.3390/antiox10030415 PMC8001687 33803273

[CR63] Hulsen T (2022) DeepVenn—a web application for the creation of area-proportional Venn diagrams using the deep learning framework Tensorflow.js. Preprint at https://arxiv.org/abs/2210.04597

[CR64] Hume RD, Kanagalingam S, Deshmukh T, Chen S, Mithieux SM, Rashid FN, Roohani I, Lu J, Doan T, Graham D et al (2023) Tropoelastin improves post-infarct cardiac function. CircRes 132:72–86 10.1161/CIRCRESAHA.122.321123 PMC9829044 36453283

[CR65] Hunter B (2025) Left ventricular myocardial molecular profile of human diabetic ischaemic cardiomyopathy. Zenodo 10.1038/s44321-025-00281-9 PMC12423312 40759793

[CR66] Hunter WG, McGarrah RW, Kelly JP, Khouri MG, Craig DM, Haynes C, Felker GM, Hernandez AF, Velazquez EJ, Kraus WE et al (2019) High-density lipoprotein particle subfractions in heart failure with preserved or reduced ejection fraction. J Am Coll Cardiol 73:177–186 30654890 10.1016/j.jacc.2018.10.059

[CR67] James J, Zhang Y, Osinska H, Sanbe A, Klevitsky R, Hewett TE, Robbins J (2000) Transgenic modeling of a cardiac troponin I mutation linked to familial hypertrophic cardiomyopathy. CircRes 87:805–811 10.1161/01.res.87.9.805 11055985

[CR68] Jia G, Hill MA, Sowers JR (2018) Diabetic cardiomyopathy. CircRes 122:624–638 10.1161/CIRCRESAHA.117.311586 PMC5819359 29449364

[CR69] Kaludercic N, Di Lisa F (2020) Mitochondrial ROS formation in the pathogenesis of diabetic cardiomyopathy. Front Cardiovasc Med 7:15 32133373 10.3389/fcvm.2020.00012 PMC7040199

[CR70] Kane AE, Sinclair DA (2018) Sirtuins and NAD(+) in the development and treatment of metabolic and cardiovascular diseases. CircRes 123:868–885 10.1161/CIRCRESAHA.118.312498 PMC6206880 30355082

[CR71] Kanehisa M (2019) Toward understanding the origin and evolution of cellular organisms. Protein Sci 28:1947–1951 31441146 10.1002/pro.3715 PMC6798127

[CR72] Kanehisa M, Furumichi M, Sato Y, Kawashima M, Ishiguro-Watanabe M (2023) KEGG for taxonomy-based analysis of pathways and genomes. Nucleic Acids Res 51:D587–D592 10.1093/nar/gkac963 PMC9825424 36300620

[CR73] Kanehisa M, Goto S (2000) KEGG: Kyoto Encyclopedia of Genes and Genomes. Nucleic Acids Res 28:27–30 10592173 10.1093/nar/28.1.27 PMC102409

[CR74] Karamanlidis G, Lee CF, Garcia-Menendez L, Kolwicz SC, Suthammarak W, Gong GH, Sedensky MM, Morgan PG, Wang W, Tian R (2013) Mitochondrial complex i deficiency increases protein acetylation and accelerates heart failure. Cell Metab 18:239–250 23931755 10.1016/j.cmet.2013.07.002 PMC3779647

[CR75] Karwi QG, Uddin GM, Ho KL, Lopaschuk GD (2018) Loss of metabolic flexibility in the failing heart. Front Cardiovasc Med 5:19 29928647 10.3389/fcvm.2018.00068 PMC5997788

[CR76] Khan MAB, Hashim MJ, Mustafa H, Baniyas MY, Al Suwaidi S, AlKatheeri R, Alblooshi FMK, Almatrooshi M, Alzaabi MEH, Al Darmaki RS et al (2020) Global epidemiology of ischemic heart disease: results from the Global Burden of Disease Study. Cureus J Med Sci 12:12 10.7759/cureus.9349 PMC7384703 32742886

[CR77] Kien B, Kolleritsch S, Kunowska N, Heier C, Chalhoub G, Tilp A, Wolinski H, Stelzl U, Haemmerle G (2022) Lipid droplet-mitochondria coupling via perilipin 5 augments respiratory capacity but is dispensable for FA oxidation. J Lipid Res 63:17 10.1016/j.jlr.2022.100172 PMC8953689 35065923

[CR78] Koay YC, Chen Y-C, Wali JA, Luk AWS, Li M, Doma H, Reimark R, Zaldivia MTK, Habtom HT, Franks AE et al (2021a) Plasma levels of trimethylamine-N-oxide can be increased with ‘healthy’ and ‘unhealthy’ diets and do not correlate with the extent of atherosclerosis but with plaque instability. Cardiovasc Res 117:435–449 32267921 10.1093/cvr/cvaa094 PMC8599768

[CR79] Koay YC, Stanton K, Kienzle V, Li MB, Yang J, Celermajer DS, O’Sullivan JF (2021b) Effect of chronic exercise in healthy young male adults: a metabolomic analysis. Cardiovasc Res 117:613–622 32239128 10.1093/cvr/cvaa051

[CR80] Koentges C, Pfeil K, Schnick T, Wiese S, Dahlbock R, Cimolai MC, Meyer-Steenbuck M, Cenkerova K, Hoffmann MM, Jaeger C et al (2015) SIRT3 deficiency impairs mitochondrial and contractile function in the heart. Basic Res Cardiol 110:20 25962702 10.1007/s00395-015-0493-6

[CR81] Kraft T, Hornemann T, Stolz M, Nier V, Wallimann T (2000) Coupling of creatine kinase to glycolytic enzymes at the sarcomeric I-band of skeletal muscle: a biochemical study in situ. J Muscle Res Cell Motil 21:691–703 11227796 10.1023/a:1005623002979

[CR82] Krahmer N, Farese RV, Walther TC (2013) Balancing the fat: lipid droplets and human disease. EMBO Mol Med 5:973–983 23740690 10.1002/emmm.201100671 PMC3721468

[CR83] Kramerova I, Kudryashova E, Wu B, Ottenheijm C, Granzier H, Spencer MJ (2008) Novel role of calpain-3 in the triad-associated protein complex regulating calcium release in skeletal muscle. Hum Mol Genet 17:3271–3280 18676612 10.1093/hmg/ddn223 PMC2566524

[CR84] Kranias EG, Hajjar RJ (2012) Modulation of cardiac contractility by the phopholamban/SERCA2a regulatome. CircRes 110:1646–1660 10.1161/CIRCRESAHA.111.259754 PMC3392125 22679139

[CR85] Kumar AA, Kelly DP, Chirinos JA (2019) Mitochondrial dysfunction in heart failure with preserved ejection fraction. Circulation 139:1435–1450 30856000 10.1161/CIRCULATIONAHA.118.036259 PMC6414077

[CR86] Kunz WS, Gellerich FN (1993) Quantification of the content of fluorescent flavoproteins in mitochondria from liver, kidney cortex, skeletal-muscle, and brain. Biochem Med Metab Biol 50:103–110 8373630 10.1006/bmmb.1993.1051

[CR87] Kuznetsov AV, Javadov S, Grimm M, Margreiter R, Ausserlechner MJ, Hagenbuchner J (2020) Crosstalk between mitochondria and cytoskeleton in cardiac cells. Cells 9:24 10.3390/cells9010222 PMC7017221 31963121

[CR88] Lal S, Li A, Allen D, Allen P, Bannon P, Cartmill T, Cooke R, Farnsworth A, Keogh A, Remedios C (2015) Best practice BioBanking of human heart tissue. Biophys Rev 7:399–406 26998172 10.1007/s12551-015-0182-6 PMC4792521

[CR89] Lal S, Nguyen L, Tezone R, Ponten F, Odeberg J, Li A, dos Remedios C (2016) Tissue microarray profiling in human heart failure. Proteomics 16:2319–2326 27364902 10.1002/pmic.201600135

[CR90] Lange S, Gehmlich K, Lun AS, Blondelle J, Hooper C, Dalton ND, Alvarez EA, Zhang XY, Bang ML, Abassi YA et al (2016) MLP and CARP are linked to chronic PKC alpha signalling in dilated cardiomyopathy. Nat Commun 7:11 10.1038/ncomms12120 PMC4931343 27353086

[CR91] Larsen S, Nielsen J, Hansen CN, Nielsen LB, Wibrand F, Stride N, Schroder HD, Boushel R, Helge JW, Dela F et al (2012) Biomarkers of mitochondrial content in skeletal muscle of healthy young human subjects. J Physiol-Lond 590:3349–3360 22586215 10.1113/jphysiol.2012.230185 PMC3459047

[CR92] Law CW, Chen Y, Shi W, Smyth GK (2014) voom: precision weights unlock linear model analysis tools for RNA-seq read counts. Genome Biol 15:R29–R29 24485249 10.1186/gb-2014-15-2-r29 PMC4053721

[CR93] Lee CC, Watkins SM, Lorenzo C, Wagenknecht LE, Il’yasova D, Chen YDI, Haffner SM, Hanley AJ (2016a) Branched-chain amino acids and insulin metabolism: the insulin resistance atherosclerosis study (IRAS). Diab Care 39:582–588 10.2337/dc15-2284 PMC4806771 26895884

[CR94] Lee CF, Chavez JD, Garcia-Menendez L, Choi Y, Roe ND, Chiao YA, Edgar JS, Goo YA, Goodlett DR, Bruce JE et al (2016b) Normalization of NAD(+) redox balance as a therapy for heart failure. Circulation 134:883 27489254 10.1161/CIRCULATIONAHA.116.022495 PMC5193133

[CR95] Lee SJ, Zhang JL, Choi AMK, Kim HP (2013) Mitochondrial dysfunction induces formation of lipid droplets as a generalized response to stress. Oxid Med Cell Longev 2013:10 10.1155/2013/327167 PMC3794647 24175011

[CR96] Leguisamo NM, Lehnen AM, Machado UF, Okamoto MM, Markoski MM, Pinto GH, Schaan BD (2012) GLUT4 content decreases along with insulin resistance and high levels of inflammatory markers in rats with metabolic syndrome. Cardiovasc Diabetol 11:100–100 22897936 10.1186/1475-2840-11-100 PMC3439702

[CR97] Lei B, Lionetti V, Young ME, Chandler MP, d’Agostino C, Kang E, Altarejos M, Matsuo K, Hintze TH, Stanley WC et al (2004) Paradoxical downregulation of the glucose oxidation pathway despite enhanced flux in severe heart failure. J Mol Cell Cardiol 36:567–576 15081316 10.1016/j.yjmcc.2004.02.004

[CR98] Li AQ, Gao M, Jiang WT, Qin Y, Gong GH (2020a) Mitochondrial dynamics in adult cardiomyocytes and heart diseases. Front Cell Dev Biol 8:14 33392184 10.3389/fcell.2020.584800 PMC7773778

[CR99] Li L, Zhao Q, Kong W (2018a) Extracellular matrix remodeling and cardiac fibrosis. Matrix Biol 68-69:490–506 29371055 10.1016/j.matbio.2018.01.013

[CR100] Li MB, Parker BL, Pearson E, Hunter B, Cao J, Koay YC, Guneratne O, James DE, Yang J, Lal S et al (2020b) Core functional nodes and sex-specific pathways in human ischaemic and dilated cardiomyopathy. Nat Commun 11:2843 32487995 10.1038/s41467-020-16584-z PMC7266817

[CR101] Li YP, Zhang DH, Kong LY, Shi HT, Tian XY, Gao L, Liu YZ, Wu LM, Du BB, Huang Z et al (2018b) Aldolase promotes the development of cardiac hypertrophy by targeting AMPK signaling. Exp Cell Res 370:78–86 29902536 10.1016/j.yexcr.2018.06.009

[CR102] Liao Y, Smyth GK, Shi W (2019) The R package Rsubread is easier, faster, cheaper and better for alignment and quantification of RNA sequencing reads. Nucleic Acids Res 47:e47–e47 30783653 10.1093/nar/gkz114 PMC6486549

[CR103] Lin YX, Ghazanfar S, Wang KYX, Gagnon-Bartsch JA, Lo KK, Su XB, Han ZG, Ormerod JT, Speed TP, Yang PY et al (2019) scMerge leverages factor analysis, stable expression, and pseudoreplication to merge multiple single-cell RNA-seq datasets. Proc Natl Acad Sci USA 116:9775–9784 31028141 10.1073/pnas.1820006116 PMC6525515

[CR104] Lipskaia L, Keuylian Z, Blirando K, Mougenot N, Jacquet A, Rouxel C, Sghairi H, Elaib Z, Blaise R, Adnot S et al (2014) Expression of sarco (endo) plasmic reticulum calcium ATPase (SERCA) system in normal mouse cardiovascular tissues, heart failure and atherosclerosis. Biochim Biophys Acta-Mol Cell Res 1843:2705–2718 10.1016/j.bbamcr.2014.08.002 PMC4159674 25110346

[CR105] Liu T, Song D, Dong JZ, Zhu PH, Liu J, Liu W, Ma XH, Zhao L, Ling SK (2017) Current understanding of the pathophysiology of myocardial fibrosis and its quantitative assessment in heart failure. Front Physiol 8:13 28484397 10.3389/fphys.2017.00238 PMC5402617

[CR106] Liu TT, Chen L, Kim E, Tran D, Phinney BS, Knowlton AA (2014) Mitochondrial proteome remodeling in ischemic heart failure. Life Sci 101:27–36 24548633 10.1016/j.lfs.2014.02.004 PMC4075482

[CR107] Lopaschuk GD, Karwi QG, Tian R, Wende AR, Abel ED (2021) Cardiac energy metabolism in heart failure. CircRes 128:1487–1513 10.1161/CIRCRESAHA.121.318241 PMC8136750 33983836

[CR108] Lu HH, Wu YM, Chang WT, Luo T, Yang YC, Cho HD, Liau I (2014) Molecular imaging of ischemia and reperfusion in vivo with mitochondria! autofluorescence. Anal Chem 86:5024–5031 24720791 10.1021/ac5006469

[CR109] Lu Q, Pan B, Bai HB, Zhao WA, Liu LJ, Li G, Liu RM, Lv TW, Huang XP, Li X et al (2022) Intranuclear cardiac troponin I plays a functional role in regulating Atp2a2 expression in cardiomyocytes. Genes Dis 9:1689–1700 36157491 10.1016/j.gendis.2021.04.007 PMC9485201

[CR110] Lu Y, Wu QF, Liao J, Zhang SS, Lu K, Yang ST, Wu YW, Dong Q, Yuan J, Zhao N et al (2021) Identification of the distinctive role of DPT in dilated cardiomyopathy: a study based on bulk and single-cell transcriptomic analysis. Ann Transl Med 9:19 34733953 10.21037/atm-21-2913 PMC8506774

[CR111] Lucas DT, Szweda LI (1999) Declines in mitochondrial respiration during cardiac reperfusion: age-dependent inactivation of alpha-ketoglutarate dehydrogenase. Proc Natl Acad Sci USA 96:6689–6693 10359773 10.1073/pnas.96.12.6689 PMC21976

[CR112] Luo GY, Wang R, Zhou H, Liu XL (2021) ALDOA protects cardiomyocytes against H/R-induced apoptosis and oxidative stress by regulating the VEGF/Notch 1/Jagged 1 pathway. Mol Cell Biochem 476:775–783 33089381 10.1007/s11010-020-03943-z

[CR113] Lynch CJ, Adams SH (2014) Branched-chain amino acids in metabolic signalling and insulin resistance. Nat Rev Endocrinol 10:723–736 25287287 10.1038/nrendo.2014.171 PMC4424797

[CR114] Lyons PJ, Mattatall NR, Ro HS (2006) Modeling and functional analysis of AEBP1, a transcriptional repressor. Proteins 63:1069–1083 16538615 10.1002/prot.20946

[CR115] Lyra-Leite DM, Petersen AP, Ariyasinghe NR, Cho N, McCain ML (2021) Mitochondrial architecture in cardiac myocytes depends on cell shape and matrix rigidity. J Mol Cell Cardiol 150:32–43 33038389 10.1016/j.yjmcc.2020.10.004 PMC11956898

[CR116] Mamidi R, Li JY, Gresham KS, Verma S, Doh CY, Li A, Lal S, dos Remedios CG, Stelzer JE (2017) Dose-dependent effects of the myosin activator omecamtiv mecarbil on cross-bridge behavior and force generation in failing human myocardium. Circ-Heart Fail 10:19 10.1161/CIRCHEARTFAILURE.117.004257 PMC5685665 29030372

[CR117] Martín MA, Gómez MA, Guillén F, Börnstein B, Campos Y, Rubio JC, de la Calzada CS, Arenas J (2000) Myocardial carnitine and carnitine palmitoyltransferase deficiencies in patients with severe heart failure. Biochim et Biophys Acta Mol Basis Dis 1502:330–336 10.1016/s0925-4439(00)00061-2 11068176

[CR118] McGavock JM, Lingvay I, Zib I, Tillery T, Salas N, Unger R, Levine BD, Raskin P, Victor RG, Szczepaniak LS (2007) Cardiac steatosis in diabetes mellitus—a H-1-magnetic resonance spectroscopy study. Circulation 116:1170–1175 17698735 10.1161/CIRCULATIONAHA.106.645614

[CR119] McKenna WJ, Maron BJ, Thiene G (2017) Classification, epidemiology, and global burden of cardiomyopathies. CircRes 121:722–730 10.1161/CIRCRESAHA.117.309711 28912179

[CR120] McLaughlin KL, Hagen JT, Coalson HS, Nelson MAM, Kew KA, Wooten AR, Fisher-Wellman KH (2020) Novel approach to quantify mitochondrial content and intrinsic bioenergetic efficiency across organs. Sci Rep 10:15 33077793 10.1038/s41598-020-74718-1 PMC7572412

[CR121] McNally EM, Mestroni L (2017) Dilated cardiomyopathy genetic determinants and mechanisms. CircRes 121:731–748 10.1161/CIRCRESAHA.116.309396 PMC5626020 28912180

[CR122] Milani-Nejad N, Janssen PML (2014) Small and large animal models in cardiac contraction research: advantages and disadvantages. Pharm Ther 141:235–249 10.1016/j.pharmthera.2013.10.007 PMC3947198 24140081

[CR123] Missov E, Calzolari C, Pau B (1997) Circulating cardiac troponin I in severe congestive heart failure. Circulation 96:2953–2958 9386162 10.1161/01.cir.96.9.2953

[CR124] Mollova M, Bersell K, Walsh S, Savla J, Das LT, Park SY, Silberstein LE, dos Remedios CG, Graham D, Colan S et al (2013) Cardiomyocyte proliferation contributes to heart growth in young humans. Proc Natl Acad Sci USA 110:1446–1451 23302686 10.1073/pnas.1214608110 PMC3557060

[CR125] Montaigne D, Marechal X, Coisne A, Debry N, Modine T, Fayad G, Potelle C, El Arid JM, Mouton S, Sebti Y et al (2014) Myocardial contractile dysfunction is associated with impaired mitochondrial function and dynamics in type 2 diabetic but not in obese patients. Circulation 130:554 24928681 10.1161/CIRCULATIONAHA.113.008476

[CR126] Morgan M, Anders S, Lawrence M, Aboyoun P, Pagès H, Gentleman R (2009) ShortRead: a bioconductor package for input, quality assessment and exploration of high-throughput sequence data. Bioinformatics 25:2607–2608 19654119 10.1093/bioinformatics/btp450 PMC2752612

[CR127] Morgenstern M, Peikert CD, Lubbert P, Suppanz I, Klemm C, Alka O, Steiert C, Naumenko N, Schendzielorz A, Melchionda L et al (2021) Quantitative high-confidence human mitochondrial proteome and its dynamics in cellular context. Cell Metab 33:2464 34800366 10.1016/j.cmet.2021.11.001 PMC8664129

[CR128] Mracek T, Drahota Z, Houstek J (2013) The function and the role of the mitochondrial glycerol-3-phosphate dehydrogenase in mammalian tissues. Biochim Biophys Acta-Bioenerg 1827:401–410 10.1016/j.bbabio.2012.11.014 23220394

[CR129] Murugasamy K, Munjal A, Sundaresan NR (2022) Emerging roles of SIRT3 in cardiac metabolism. Front Cardiovasc Med 9:15 10.3389/fcvm.2022.850340 PMC8971545 35369299

[CR130] Muslimova EF, Rebrova TY, Kondratieva DS, Akhmedov SD, Afanasiev SA (2020) Expression of the Ca2+-ATPase SERCA2a (ATP2A2) gene and the ryanodine receptor (RYR2) gene in patients with chronic heart failure. Russ J Genet 56:843–848

[CR131] Nan ND, Klincumhom N, Trachoo V, Everts V, Ferreira JN, Osathanon T, Pavasant P (2024) Periostin‐integrin interaction regulates force‐induced TGF‐β1 and α‐SMA expression by hPDLSCs. Oral Dis 30:2570–2579 10.1111/odi.14691 37466141

[CR132] Neglia D, De Caterina A, Marraccini P, Natali A, Ciardetti M, Vecoli C, Gastaldelli A, Ciociaro D, Pellegrini P, Testa R et al (2007) Impaired myocardial metabolic reserve and substrate selection flexibility during stress in patients with idiopathic dilated cardiomyopathy. Am J Physiol-Heart Circ Physiol 293:H3270–H3278 17921325 10.1152/ajpheart.00887.2007

[CR133] Newgard CB, An J, Bain JR, Muehlbauer MJ, Stevens RD, Lien LF, Haqq AM, Shah SH, Arlotto M, Slentz CA et al (2009) A branched-chain amino acid-related metabolic signature that differentiates obese and lean humans and contributes to insulin resistance. Cell Metab 9:311–326 19356713 10.1016/j.cmet.2009.02.002 PMC3640280

[CR134] Niu XF, Stancliffe E, Gelman SJ, Wang LJ, Schwaiger-Haber M, Rowles JL, Shriver LP, Patti GJ (2023) Cytosolic and mitochondrial NADPH fluxes are independently regulated. Nat Chem Biol 19:837 36973440 10.1038/s41589-023-01283-9 PMC12175224

[CR135] Nyman K, Graner M, Pentikainen MO, Lundbom J, Hakkarainen A, Siren R, Nieminen MS, Taskinen MR, Lundbom N, Lauerma K (2013) Cardiac steatosis and left ventricular function in men with metabolic syndrome. J Cardiovasc Magn Reson 15:11 24228979 10.1186/1532-429X-15-103 PMC3842676

[CR136] Ohkusa T, Noma T, Ueyama T, Hisamatsu Y, Yano M, Esato K, Nakazawa A, Matsuzaki M (1997) Differences in sarcoplasmic reticulum gene expression in myocardium from patients undergoing cardiac surgery. Quantification of steady-state levels of messenger RNA using the reverse transcription polymerase chain reaction. Heart Vessels 12:1–9 9288554 10.1007/BF01747496

[CR137] Okamoto O, Fujiwara S (2006) Dermatopontin, a novel player in the biology of the extracellular matrix. Connect Tissue Res 47:177–189 16987749 10.1080/03008200600846564

[CR138] Palomer X, Roman-Azcona MS, Pizarro-Delgado J, Planavila A, Villarroya F, Valenzuela-Alcaraz B, Crispi F, Sepulveda-Martinez A, Miguel-Escalada I, Ferrer J et al (2020) SIRT3-mediated inhibition of FOS through histone H3 deacetylation prevents cardiac fibrosis and inflammation. Signal Transduct Target Ther 5:10 32296036 10.1038/s41392-020-0114-1 PMC7046732

[CR139] Pan B, Kusko R, Xiao W, Zheng Y, Liu Z, Xiao C, Sakkiah S, Guo W, Gong P, Zhang C et al (2019) Similarities and differences between variants called with human reference genome HG19 or HG38. BMC Bioinforma 20:101–101 10.1186/s12859-019-2620-0 PMC6419332 30871461

[CR140] Paolillo S, Marsico F, Prastaro M, Renga F, Esposito L, De Martino F, Di Napoli P, Esposito I, Ambrosio A, Ianniruberto M et al (2019) Diabetic cardiomyopathy definition, diagnosis, and therapeutic implications. Heart Fail Clin 15:341 31079692 10.1016/j.hfc.2019.02.003

[CR141] Pelat M, Barbe F, Daveu C, Ly-Nguyen L, Lartigue T, Marque S, Tavares G, Ballet V, Guillon JM, Steinmeyer K et al (2021) SAR340835, a novel selective Na+/Ca2+ exchanger inhibitor, improves cardiac function and restores sympathovagal balance in heart failure. J Pharm Exp Ther 377:293–304 10.1124/jpet.120.000238 33602875

[CR142] Perez-Gomez R, Magnin V, Mihajlovic Z, Slaninova V, Krejci A (2020) Downregulation of respiratory complex I mediates major signalling changes triggered by TOR activation. Sci Rep 10:13 32157127 10.1038/s41598-020-61244-3 PMC7064613

[CR143] Perez-Riverol Y, Bai J, Bandla C, García-Seisdedos D, Hewapathirana S, Kamatchinathan S, Kundu Deepti J, Prakash A, Frericks-Zipper A, Eisenacher M et al (2022) The PRIDE database resources in 2022: a hub for mass spectrometry-based proteomics evidences. Nucleic Acids Res 50:D543–D552 34723319 10.1093/nar/gkab1038 PMC8728295

[CR144] Perrone-Filardi P, Paolillo S, Costanzo P, Savarese G, Trimarco B, Bonow RO (2015) The role of metabolic syndrome in heart failure. Eur Heart J 36:2630 26242711 10.1093/eurheartj/ehv350

[CR145] Polizzotti BD, Ganapathy B, Walsh S, Choudhury S, Ammanamanchi N, Bennett DG, dos Remedios CG, Haubner BJ, Penninger JM, Kuhn B (2015) Neuregulin stimulation of cardiomyocyte regeneration in mice and human myocardium reveals a therapeutic window. Sci Transl Med 7:13 10.1126/scitranslmed.aaa5171 PMC5360874 25834111

[CR146] Pott C, Eckardt L, Goldhaber JI (2011) Triple threat: the Na+/Ca2+ exchanger in the pathophysiology of cardiac arrhythmia, ischemia and heart failure. Curr Drug Targets 12:737–747 21291388 10.2174/138945011795378559 PMC4406235

[CR147] Qiao B, Liu X, Wang B, Wei S (2024) The role of periostin in cardiac fibrosis. Heart Fail Rev 29:191–206 10.1007/s10741-023-10361-y 37870704

[CR148] Rath S, Sharma R, Gupta R, Ast T, Chan C, Durham TJ, Goodman RP, Grabarek Z, Haas ME, Hung WHW et al (2021) MitoCarta3.0: an updated mitochondrial proteome now with sub-organelle localization and pathway annotations. Nucleic Acids Res 49:D1541–D1547 33174596 10.1093/nar/gkaa1011 PMC7778944

[CR149] Read AD, Bentley RET, Archer SL, Dunham-Snary KJ (2021) Mitochondrial iron-sulfur clusters: structure, function, and an emerging role in vascular biology. Redox Biol 47:17 10.1016/j.redox.2021.102164 PMC8577454 34656823

[CR150] Reinecke H, Studer R, Vetter R, Holtz J, Drexler H (1996) Cardiac Na+/Ca2+ exchange activity in patients with end-stage heart failure. Cardiovasc Res 31:48–54 8849588

[CR151] Ritterhoff J, Tian R (2023) Metabolic mechanisms in physiological and pathological cardiac hypertrophy: new paradigms and challenges. Nat Rev Cardiol 20:812–829 10.1038/s41569-023-00887-x 37237146

[CR152] Ro HS, Zhang L, Majdalawieh A, Kim SW, Wu X, Lyons PJ, Webber C, Ma H, Reidy SP, Boudreau A et al (2007) Adipocyte enhancer-binding protein 1 modulates adiposity and energy homeostasis. Obesity 15:288–302 17299101 10.1038/oby.2007.569

[CR153] Romashko DN, Marban E, O’Rourke B (1998) Subcellular metabolic transients and mitochondrial redox waves in heart cells. Proc Natl Acad Sci USA 95:1618–1623 9465065 10.1073/pnas.95.4.1618 PMC19119

[CR154] Rosenblatt-Velin N, Montessuit C, Papageorgiou I, Terrand M, Lerch R (2001) Postinfarction heart failure in rats is associated with upregulation of GLUT-1 and downregulation of genes of fatty acid metabolism. Cardiovasc Res 52:407–416 11738057 10.1016/s0008-6363(01)00393-5

[CR155] Ruiz M, Labarthe F, Fortier A, Bouchard B, Legault JT, Bolduc V, Rigal O, Chen J, Ducharme A, Crawford PA et al (2017) Circulating acylcarnitine profile in human heart failure: a surrogate of fatty acid metabolic dysregulation in mitochondria and beyond. Am J Physiol-Heart Circ Physiol 313:H768–H781 28710072 10.1152/ajpheart.00820.2016

[CR156] Schindelin J, Arganda-Carreras I, Frise E, Kaynig V, Longair M, Pietzsch T, Preibisch S, Rueden C, Saalfeld S, Schmid B et al (2012) Fiji: an open-source platform for biological-image analysis. Nat Methods 9:676–682 22743772 10.1038/nmeth.2019 PMC3855844

[CR157] Schroeder MA, Atherton HJ, Dodd MS, Lee P, Cochlin LE, Radda GK, Clarke K, Tyler DJ (2012) The cycling of acetyl-coenzyme A through acetylcarnitine buffers cardiac substrate supply a hyperpolarized C-13 magnetic resonance study. Circ-Cardiovasc Imaging 5:201–U282 22238215 10.1161/CIRCIMAGING.111.969451 PMC3378498

[CR158] Sebastiao MJ, Almeida HV, Serra M, Hamdani N, Saraiva F, Lourenco AP, Barros AS, Vasques-Novoa F, Leite-Moreira A, Alves PM et al (2022) Unveiling human proteome signatures of heart failure with preserved ejection fraction. Biomedicines 10:11 10.3390/biomedicines10112943 PMC9687619 36428511

[CR159] Seo IR, Moh SH, Lee EH, Meissner G, Kim DH (2006) Aldolase potentiates DIDS activation of the ryanodine receptor in rabbit skeletal sarcoplasmic reticulum. Biochem J 399:325–333 16817780 10.1042/BJ20060701 PMC1609923

[CR160] Sequeira V, Najafi A, Wijnker PJM, dos Remedios CG, Michels M, Kuster DWD, van der Velden J (2015) ADP-stimulated contraction: a predictor of thin-filament activation in cardiac disease. Proc Natl Acad Sci USA 112:E7003–E7012 26621701 10.1073/pnas.1513843112 PMC4687530

[CR161] Shackelford JE, Lebherz HG (1983) Synthesis and secretion of apolipoprotein-a1 by chick breast muscle. J Biol Chem 258:7175–7180 6406496

[CR162] Shah AM, Myhre PL, Arthur V, Dorbala P, Rasheed H, Buckley LF, Claggett B, Liu G, Ma J, Nguyen NQ et al (2024) Large scale plasma proteomics identifies novel proteins and protein networks associated with heart failure development. Nat Commun 15:528–528 38225249 10.1038/s41467-023-44680-3 PMC10789789

[CR163] Shao D, Tian R (2016) Glucose transporters in cardiac metabolism and hypertrophy. Compr Physiol 6:331–351 10.1002/cphy.c150016 PMC4760112 26756635

[CR164] Sletten AC, Peterson LR, Schaffer JE (2018) Manifestations and mechanisms of myocardial lipotoxicity in obesity. J Intern Med 284:478–491 29331057 10.1111/joim.12728 PMC6045461

[CR165] Stanley WC, Recchia FA, Lopaschuk GD (2005) Myocardial substrate metabolism in the normal and failing heart. Physiol Rev 85:1093–1129 15987803 10.1152/physrev.00006.2004

[CR166] Stehlik JMDMPH, Edwards LBP, Kucheryavaya AYMS, Benden CMD, Christie JDMDMS, Dobbels FP, Kirk RMAFF, Rahmel AOMD, Hertz MIMD (2011) The registry of the international society for heart and lung transplantation: Twenty-eighth Adult Heart Transplant Report—2011. J Heart Lung Transplant 30:1078–1094 21962016 10.1016/j.healun.2011.08.003

[CR167] Stride N, Larsen S, Hey-Mogensen M, Sander K, Lund JT, Gustafsson F, Kober L, Dela F (2013) Decreased mitochondrial oxidative phosphorylation capacity in the human heart with left ventricular systolic dysfunction. Eur J Heart Fail 15:150–157 23115323 10.1093/eurjhf/hfs172

[CR168] Studer R, Reinecke H, Bilger J, Eschenhagen T, Bohm M, Hasenfuss G, Just H, Holtz J, Drexler H (1994) Gene-expression of the cardiac Na+-Ca2+ exchanger in end-stage human heart-failure. CircRes 75:443–453 10.1161/01.res.75.3.443 8062418

[CR169] Sud M, Fahy E, Cotter D, Azam K, Vadivelu I, Burant C, Edison A, Fiehn O, Higashi R, Nair KS et al (2016) Metabolomics Workbench: an international repository for metabolomics data and metadata, metabolite standards, protocols, tutorials and training, and analysis tools. Nucleic Acids Res 44:D463–D470 26467476 10.1093/nar/gkv1042 PMC4702780

[CR170] Sundaresan NR, Bindu S, Pillai VB, Samant S, Pan Y, Huang JY, Gupta M, Nagalingam RS, Wolfgeher D, Verdin E et al (2016) SIRT3 blocks aging-associated tissue fibrosis in mice by deacetylating and activating glycogen synthase kinase 3 beta. Mol Cell Biol 36:678–692 10.1128/MCB.00586-15 PMC4760222 26667039

[CR171] Szklarczyk D, Gable AL, Lyon D, Junge A, Wyder S, Huerta-Cepas J, Simonovic M, Doncheva NT, Morris JH, Bork P et al (2019) STRING v11: protein-protein association networks with increased coverage, supporting functional discovery in genome-wide experimental datasets. Nucleic Acids Res 47:D607–D613 30476243 10.1093/nar/gky1131 PMC6323986

[CR172] Szklarczyk D, Kirsch R, Koutrouli M, Nastou K, Mehryary F, Hachilif R, Gable AL, Fang T, Doncheva NT, Pyysalo S et al (2023) The STRING database in 2023: protein-protein association networks and functional enrichment analyses for any sequenced genome of interest. Nucleic Acids Res 51:D638–D646 36370105 10.1093/nar/gkac1000 PMC9825434

[CR173] Tai ES, Tan MLS, Stevens RD, Low YL, Muehlbauer MJ, Goh DLM, Ilkayeva OR, Wenner BR, Bain JR, Lee JJM et al (2010) Insulin resistance is associated with a metabolic profile of altered protein metabolism in Chinese and Asian-Indian men. Diabetologia 53:757–767 20076942 10.1007/s00125-009-1637-8 PMC3753085

[CR174] Tang D, Chen M, Huang X, Zhang G, Zeng L, Zhang G, Wu S, Wang Y (2023) SRplot: a free online platform for data visualization and graphing. PLoS ONE 18:e0294236–e0294236 37943830 10.1371/journal.pone.0294236 PMC10635526

[CR175] Tao YY, Wei X, Yue Y, Wang JX, Li JZ, Shen L, Lu GY, He Y, Zhao SD, Zhao F et al (2021) Extracellular vesicle-derived AEBP1 mRNA as a novel candidate biomarker for diabetic kidney disease. J Transl Med 19:15 34332599 10.1186/s12967-021-03000-3 PMC8325821

[CR176] Tarugi P, Reggiani D, Ottaviani E, Ferrari S, Tiozzo R, Calandra S (1989) Plasma-lipoproteins, tissue cholesterol overload, and skeletal-muscle apolipoprotein-a-l synthesis in the developing chick. J Lipid Res 30:9–22 2493058

[CR177] Thomas PD, Ebert D, Muruganujan A, Mushayahama T, Albou LP, Mi HY (2022) PANTHER: making genome-scale phylogenetics accessible to all. Protein Sci 31:8–22 34717010 10.1002/pro.4218 PMC8740835

[CR178] Tian R, Abel ED (2001) Responses of GLUT4-deficient hearts to ischemia underscore the importance of glycolysis. Circulation 103:2961–2966 11413087 10.1161/01.cir.103.24.2961

[CR179] Tobias DK, Lawler PR, Harada PH, Demler OV, Ridker PM, Manson JE, Cheng S, Mora S (2018) Circulating branched-chain amino acids and incident cardiovascular disease in a prospective cohort of US women. Circ-Genom Precis Med 11:9 10.1161/CIRCGEN.118.002157 PMC5880282 29572205

[CR180] Travers JG, Kamal FA, Robbins J, Yutzey KE, Blaxall BC (2016) Cardiac fibrosis the fibroblast awakens. CircRes 118:1021–1040 10.1161/CIRCRESAHA.115.306565 PMC4800485 26987915

[CR181] Tremblay F, Krebs M, Dombrowski L, Brehm A, Bernroider E, Roth E, Nowotny P, Waldhausl W, Marette A, Roden M (2005) Overactivation of S6 kinase 1 as a cause of human insulin resistance during increased amino acid availability. Diabetes 54:2674–2684 16123357 10.2337/diabetes.54.9.2674

[CR182] Uddin GM, Zhang LY, Shah S, Fukushima A, Wagg CS, Gopal K, Al Batran R, Pherwani S, Ho KL, Boisvenue J et al (2019) Impaired branched chain amino acid oxidation contributes to cardiac insulin resistance in heart failure. Cardiovasc Diabetol 18:12 31277657 10.1186/s12933-019-0892-3 PMC6610921

[CR183] Uhlén M, Karlsson MJ, Hober A, Svensson A-S, Scheffel J, Kotol D, Zhong W, Tebani A, Strandberg L, Edfors F et al (2019) The human secretome. Sci Signal 12:eaaz0274 10.1126/scisignal.aaz0274 31772123

[CR184] Unamuno X, Gómez-Ambrosi J, Ramírez B, Rodríguez A, Becerril S, Valentí V, Moncada R, Silva C, Salvador J, Frühbeck G et al (2020) Dermatopontin, a novel adipokine promoting adipose tissue extracellular matrix remodelling and inflammation in obesity. J Clin Med 9:14 10.3390/jcm9041069 PMC7230369 32283761

[CR185] Valsecchi F, Monge C, Forkink M, de Groof AJC, Benard G, Rossignol R, Swarts HG, van Emst-de Vries SE, Rodenburg RJ, Calvaruso MA et al (2012) Metabolic consequences of NDUFS4 gene deletion in immortalized mouse embryonic fibroblasts. Biochim Biophys Acta-Bioenerg 1817:1925–1936 10.1016/j.bbabio.2012.03.006 22430089

[CR186] van Heesch S, Witte F, Schneider-Lunitz V, Schulz JF, Adami E, Faber AB, Kirchner M, Maatz H, Blachut S, Sandmann CL et al (2019) The translational landscape of the human heart. Cell 178:242 31155234 10.1016/j.cell.2019.05.010

[CR187] Vangheluwe P, Schuermans M, Raeymaekers L, Wuytack F (2007) Tight interplay between the Ca2+ affinity of the cardiac SERCA2 Ca2+ pump and the SERCA2 expression level. Cell Calcium 42:281–289 17306367 10.1016/j.ceca.2007.01.001

[CR188] Vendelin M, Beraud N, Guerrero K, Andrienko T, Kuznetsov AV, Olivares J, Kay L, Saks VA (2005) Mitochondrial regular arrangement in muscle cells: a “crystal-like” pattern. Am J Physiol-Cell Physiol 288:C757–C767 15496480 10.1152/ajpcell.00281.2004

[CR189] Vendelin M, Kongas O, Saks V (2000) Regulation of mitochondrial respiration in heart cells analyzed by reaction-diffusion model of energy transfer. Am J Physiol-Cell Physiol 278:C747 10751324 10.1152/ajpcell.2000.278.4.C747

[CR190] Vetter R, Rehfeld U, Reissfelder C, Weiss W, Wagner KD, Gunther J, Hammes A, Tschope C, Dillmann W, Paul M (2002) Transgenic overexpression of the sarcoplasmic reticulum Ca2+ ATPase improves reticular Ca2+ handling in normal and diabetic rat hearts. FASEB J 16:1657 12206992 10.1096/fj.01-1019fje

[CR191] Vinnakota KC, Bassingthwaighte JB (2004) Myocardial density and composition: a basis for calculating intracellular metabolite concentrations. Am J Physiol-Heart Circ Physiol 286:H1742–H1749 14693681 10.1152/ajpheart.00478.2003

[CR192] Voros G, Ector J, Garweg C, Droogne W, Van Cleemput J, Peersman N, Vermeersch P, Janssens S (2018) Increased cardiac uptake of ketone bodies and free fatty acids in human heart failure and hypertrophic left ventricular remodeling. Circ-Heart Fail 11:9 10.1161/CIRCHEARTFAILURE.118.004953 30562098

[CR193] Weintraub RG, Semsarian C, Macdonald P (2017) Dilated cardiomyopathy. Lancet 390:400–414 28190577 10.1016/S0140-6736(16)31713-5

[CR194] Weiss RG, Gerstenblith G, Bottomley PA (2005) ATP flux through creatine kinase in the normal, stressed, and failing human heart. Proc Natl Acad Sci USA 102:808–813 15647364 10.1073/pnas.0408962102 PMC545546

[CR195] Wende AR, Abel ED (2010) Lipotoxicity in the heart. Biochim Biophys Acta Mol Cell Biol Lipids 1801:311–319 10.1016/j.bbalip.2009.09.023 PMC2823976 19818871

[CR196] Wilshaw J, Boswood A, Chang YM, Sands CJ, Camuzeaux S, Lewis MR, Xia D, Connolly DJ (2022) Evidence of altered fatty acid metabolism in dogs with naturally occurring valvular heart disease and congestive heart failure. Metabolomics 18:12 35635592 10.1007/s11306-022-01887-7 PMC9151558

[CR197] Wu F, Yang F, Vinnakota KC, Beard DA (2007) Computer modeling of mitochondrial tricarboxylic acid cycle, oxidative phosphorylation, metabolite transport, and electrophysiology. J Biol Chem 282:24525–24537 17591785 10.1074/jbc.M701024200

[CR198] Wu TZ, Hu EQ, Xu SB, Chen MJ, Guo PF, Dai ZH, Feng TZ, Zhou L, Tang WL, Zhan L et al (2021) clusterProfiler 4.0: a universal enrichment tool for interpreting omics data. Innovation 2:11 10.1016/j.xinn.2021.100141 PMC8454663 34557778

[CR199] Wust RCI, Helmes M, Stienen GJM (2015) Rapid changes in NADH and flavin autofluorescence in rat cardiac trabeculae reveal large mitochondrial complex II reserve capacity. J Physiol 593:1829–1840 25640645 10.1113/jphysiol.2014.286153 PMC4405745

[CR200] Xie N, Zhang L, Gao W, Huang CH, Huber PE, Zhou XB, Li CL, Shen GB, Zou BW (2020) NAD(+)metabolism: pathophysiologic mechanisms and therapeutic potential. Signal Transduct Target Ther 5:37 33028824 10.1038/s41392-020-00311-7 PMC7539288

[CR201] Xu D, Li T, Wang RK, Mu R (2021) Expression and pathogenic analysis of integrin family genes in systemic sclerosis. Front Med 8:10 10.3389/fmed.2021.674523 PMC8329247 34355002

[CR202] Yang Y, Zhao M, He X, Wu Q, Li DL, Zang WJ (2021) Pyridostigmine protects against diabetic cardiomyopathy by regulating vagal activity, gut microbiota, and branched-chain amino acid catabolism in diabetic mice. Front Pharm 12:17 10.3389/fphar.2021.647481 PMC8167056 34084135

[CR203] Yoshihisa A, Watanabe S, Yokokawa T, Misaka T, Sato T, Suzuki S, Oikawa M, Kobayashi A, Takeishi Y (2017) Associations between acylcarnitine to free carnitine ratio and adverse prognosis in heart failure patients with reduced or preserved ejection fraction. ESC Heart Fail 4:360–364 28772033 10.1002/ehf2.12176 PMC5542723

[CR204] Yurista SR, Matsuura TR, Sillje HHW, Nijholt KT, McDaid KS, Shewale SV, Leone TC, Newman JC, Verdin E, van Veldhuisen DJ et al (2021) Ketone ester treatment improves cardiac function and reduces pathologic remodeling in preclinical models of heart failure. Circ-Heart Fail 14:112–124 10.1161/CIRCHEARTFAILURE.120.007684 PMC7819534 33356362

[CR205] Zhang L, Reidy SP, Nicholson TE, Lee H, Majdalawieh A, Webber C, Stewart BR, Dolphin P, Ro HS (2005) The role of AEBP1 in sex-specific diet-induced obesity. Mol Med 11:39–47 16307171 10.2119/2005-00021.Ro PMC1449517

[CR206] Zhang Y (2025) Left ventricular myocardial molecular profile of human diabetic ischaemic cardiomyopathy. Zenodo 10.1038/s44321-025-00281-9 PMC12423312 40759793

[CR207] Zhao S, Feng XF, Huang T, Luo HH, Chen JX, Zeng J, Gu MY, Li J, Sun XY, Sun D et al (2020) The association between acylcarnitine metabolites and cardiovascular disease in Chinese patients with type 2 diabetes Mellitus. Front Endocrinol 11:9 10.3389/fendo.2020.00212 PMC7214635 32431666

[CR208] Zhao YM, Godier-Furnemont A, Brown LM, Fine B, Vunjak-Novakovic G, Bax NAM, Bouten CVC (2022) Changes in extracellular matrix in failing human non-ischemic and ischemic hearts with mechanical unloading. J Mol Cell Cardiol 166:137–151 35219725 10.1016/j.yjmcc.2022.02.003 PMC9035113

[CR209] Zhou PL, Li M, Han XW, Bi YH, Zhang WG, Wu ZY, Wu G (2019) Perilipin 5 deficiency promotes atherosclerosis progression through accelerating inflammation, apoptosis, and oxidative stress. J Cell Biochem 120:19107–19123 31297870 10.1002/jcb.29238

