# Supplementary material for: Left ventricular myocardial molecular profile of human diabetic ischaemic cardiomyopathy
Source: EMBO Mol Med. 2025 Aug 4;17(9):2483–524. doi: 10.1038/s44321-025-00281-9 (PMC12423312; doi:10.1038/s44321-025-00281-9)
Supplement: Supplementary file 1 — Appendix [file 44321_2025_281_MOESM1_ESM.pdf]

Left ventricular myocardial molecular profile of human diabetic ischaemic cardiomyopathy

Appendix

Page 2 Appendix Figure S1. Human left ventricular myocardial differential analysis in protein, metabolite, and lipid abundance between heart failure with (HF-DM) and without diabetes (HF-No DM) and age-matched donors (AMD).

Page 4 Appendix Figure S2. Influences of diabetes in heart failure (HF), and body mass index across healthy and HF phenotypes on myocardium molecule abundance.

Page 6 Appendix Figure S3. Brightfield microscopy example of avoided macroscopic myocardial scar tissue and immunofluorescent microscopy control images.

Appendix Figure S1

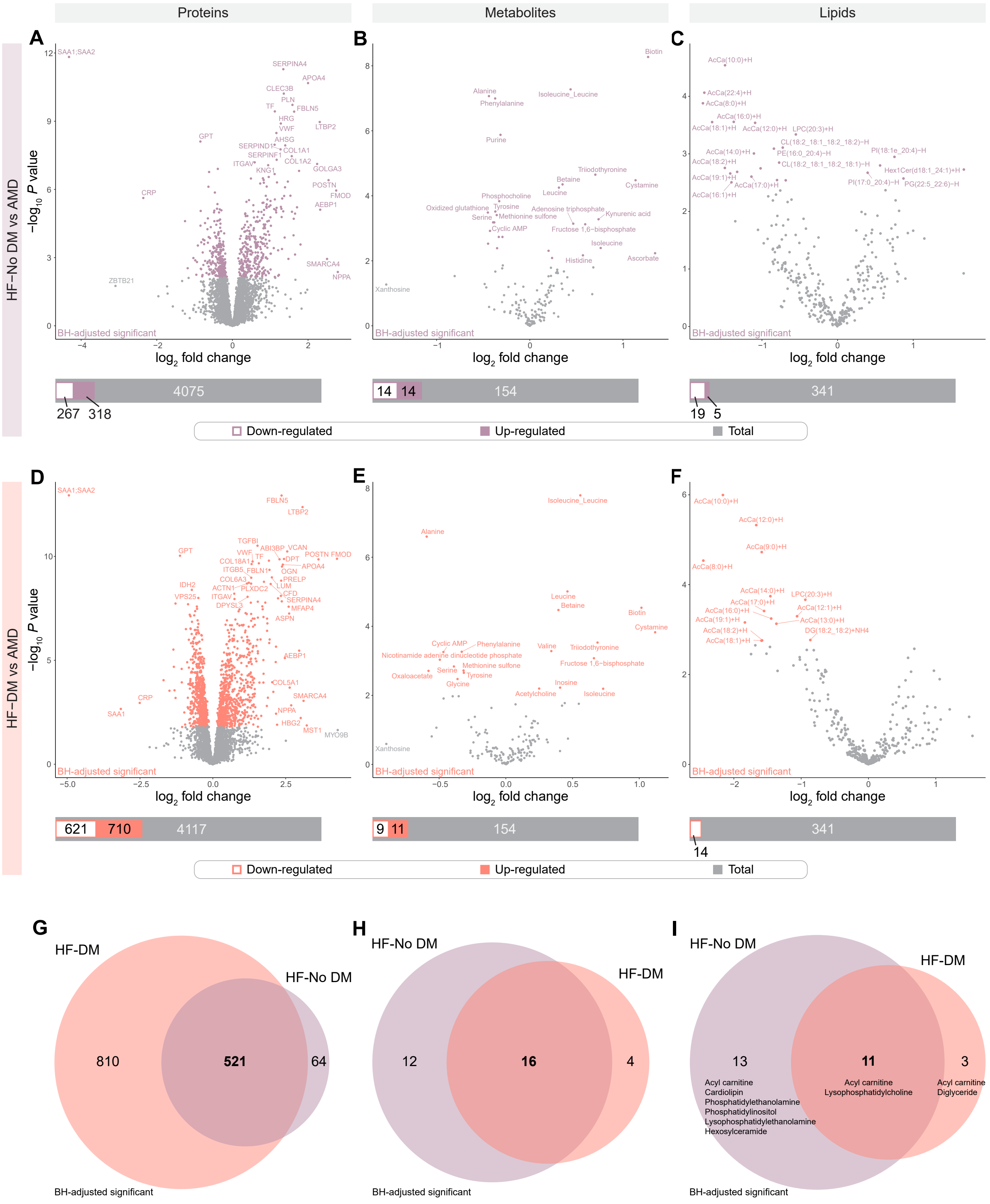

Appendix Figure S1. Human left ventricular myocardial differential analysis in protein, metabolite, and lipid abundance between heart failure with (HF-DM) and without diabetes (HF-No DM) and age-matched donors (AMD).

A-F, Differential abundance was determined following Benjamini-Hochberg false discovery rate adjustment (FDR) of *P* values (FDR < 0.05). Analyses were performed using a moderated t-test with the limma package (version 3.56.2) in R (version 4.3.1) following $\log_2$ transformation. HF-DM n = 23, HF-No DM n = 34 (proteomics and metabolomics) and 33 (lipidomics), AMD n = 20 (proteomics and metabolomics) and 19 (lipidomics). Superimposed bar plots summarise the number of significantly down-regulated (white-filled bar) and up-regulated (colour-filled bar) molecules relative to the total number of molecules analysed (grey bar). Down and up-regulation was defined as significance of molecule abundance in the heart failure condition compared to healthy age-matched donors (AMD). **A**-**C**, HF-No DM vs AMD. **D**-**F**, HF-DM vs AMD. **G**-**I**, HF-DM and HF-No DM vs AMD FDR significant differentially abundant proteins, metabolites, and lipids, respectively. **I**, Lipid classes annotated in order of the number of lipids which were FDR significant in that particular class.

Appendix Figure S2

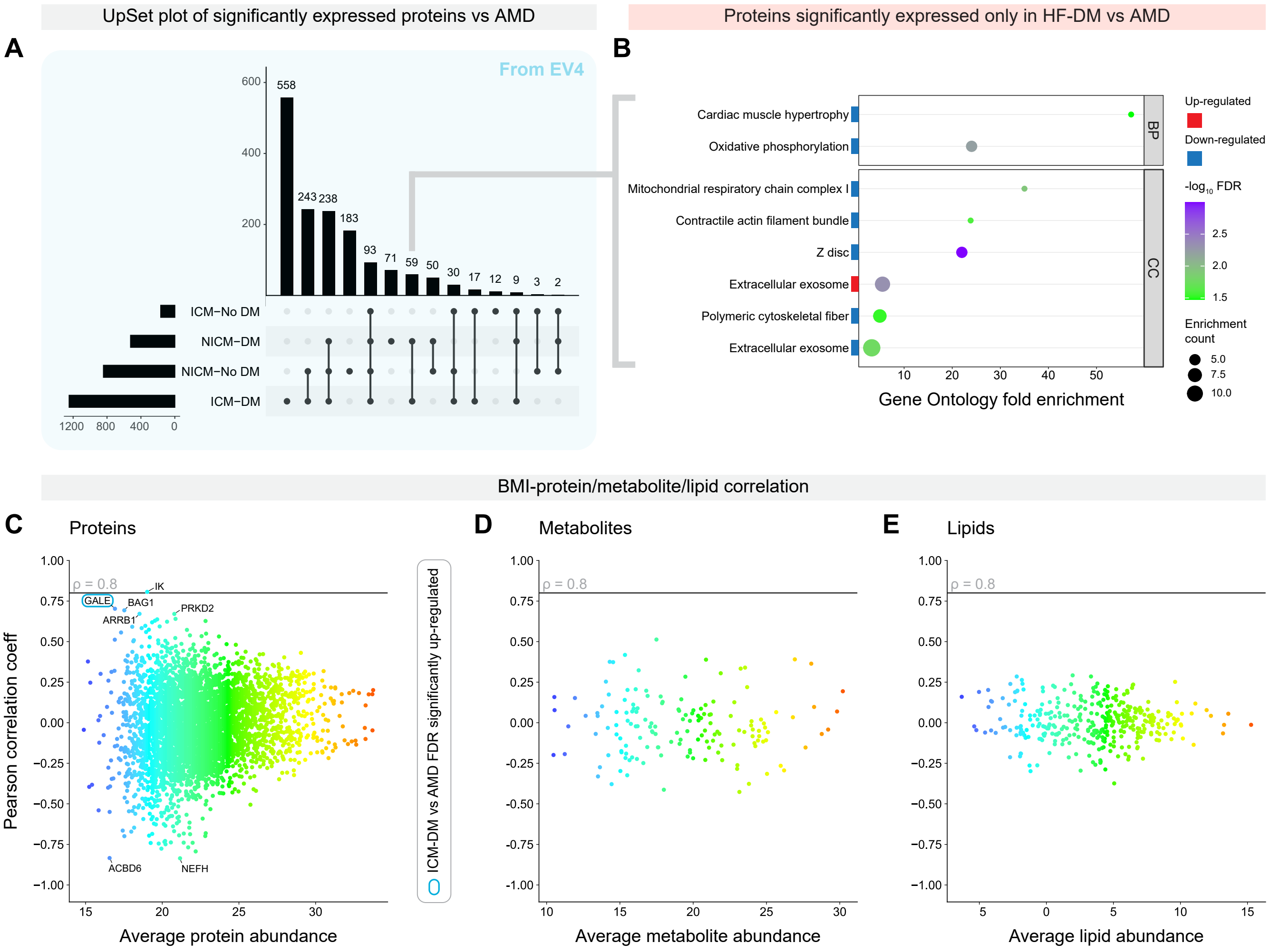

Appendix Figure S2. Influences of diabetes in heart failure (HF), and body mass index across healthy and HF phenotypes on myocardium molecule abundance.

A, UpSet plot from Fig. EV4A summarising significantly expressed proteins in all heart failure conditions vs age-matched donors (AMD). **B**, Gene Ontology (GO) analysis by PANTHER (http://geneontology.org/, PANTHER17.0) enrichment bubble plot showing selected significantly enriched Biological Process (BP), Cellular Component (CC), and Molecular Function (MF) nodes from proteins which were significantly differentially expressed in HF with diabetes (HF-DM; ICM-DM and NICM-DM) vs AMD. Nodes with an FDR < 0.05 were considered statistically significant. This plot was the combination of two separate GO analyses; one from down-regulated proteins compared to AMD (blue) and one from up-regulated proteins compared to AMD (red). Enrichment count, represented as the size of the bubble, is the number of significant proteins in that particular node. GO fold enrichment is calculated as observed enrichment count/expected enrichment count from a random set of gene symbols of equal an input set size. **C**-**E**, BMI-molecule (protein, metabolite, and lipid) Pearson correlation coefficient plot to determine the strength of the relationship between BMI and molecule abundance (potential additional confounder in ICM-DM HF group which has the highest BMI). Molecules with a correlation coefficient of $\rho > 0.8$ were accepted as being correlated. Average molecule abundance was calculated across all samples with a BMI value. Selected proteins with a high $\pm \rho$ were annotated wherein those with a blue border were also FDR significantly expressed in ICM-DM vs AMD.

Appendix Figure S3

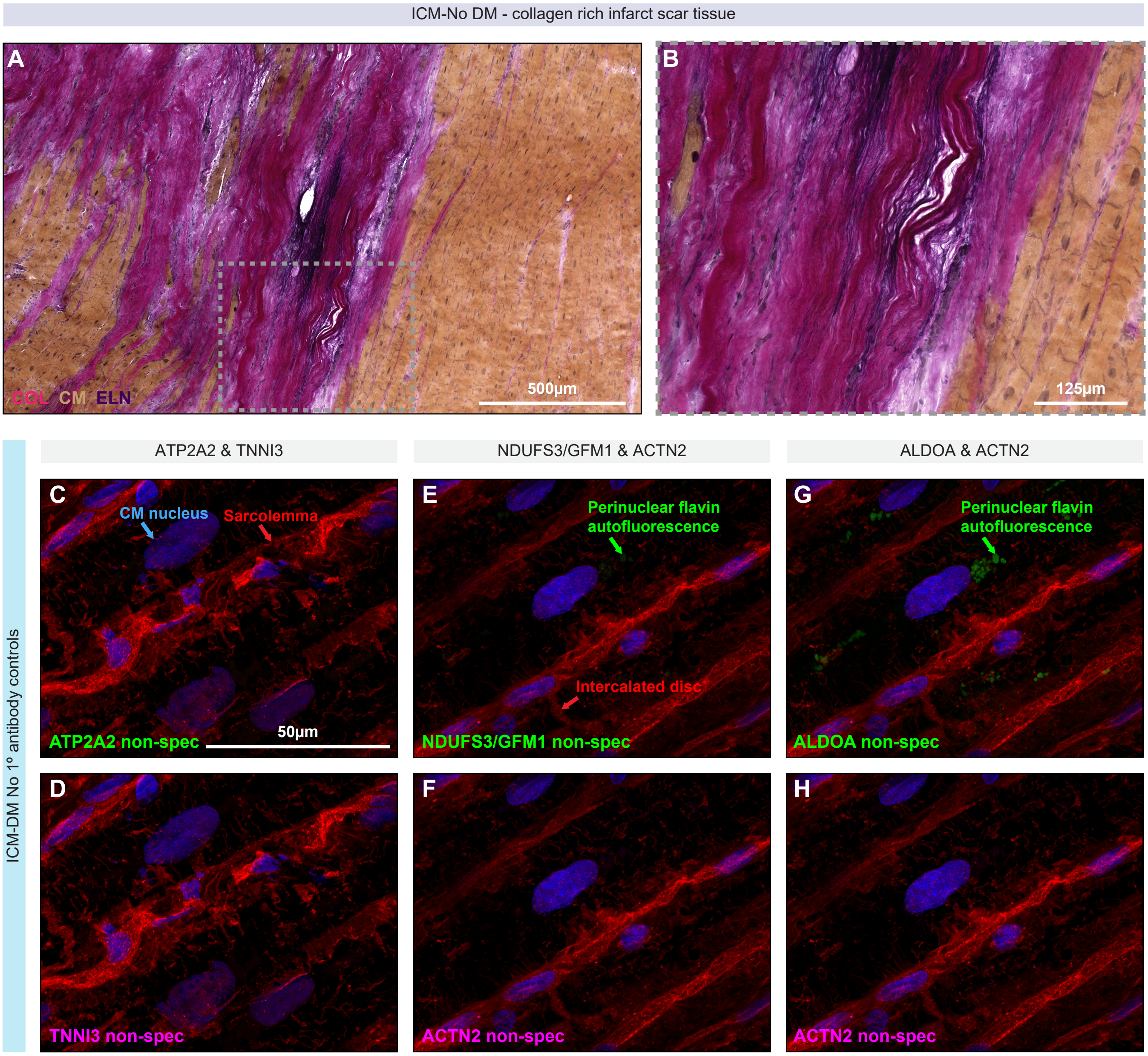

Appendix Figure S3. Brightfield microscopy example of avoided macroscopic myocardial scar tissue and immunofluorescent microscopy control images.

A-B, Bright-field microscopy images of Verhoeff-Van Gieson stain of ischaemic cardiomyopathy without diabetes (ICM-No DM) macroscopic infarct/replacement fibrosis scar tissue with an inset at a higher magnification. Collagens (COL), elastin (ELN, dark purple), cardiomyocytes (CM, brown), and nuclei (dark blue) identifiable. Macroscopic scar tissue in all heart failure tissues was avoided for all quantitative analyses (mass spectrometry and RNA sequencing) as well as in all microscopy. **C-H**, ICM-DM no primary antibody control stains to detect any autofluorescence/non-specific fluorophore-conjugated secondary antibody labelling within the myocardium. Columns are in reference to the sections co-stained with that combination of primary antibodies shown in Figures 6D-E and EV8E-J. All paired sections (AMD and ICM-DM) were cut (16µm at -16°C), co-stained, imaged, and post-processed under identical conditions at the same time; antibodies and fluorophore conjugated stains were from the same master mix, sections were imaged under identical settings, and brightness/contrast adjusted identically in Fiji/ImageJ post-processing. All fluorophore conjugated secondary antibodies were applied with a concentration of 5µg/mL to all sections. NDUFS3, GFM1 and ALDOA featured stains were stained in the same batch, so were referenced to the same no primary antibody control section. Experimental condition (ICM-DM) was chosen for no primary antibody control. Membranes, particularly the sarcolemma, were stained with fluorophore-conjugated wheat germ agglutinin (red) and nuclei were stained using DAPI (blue). Panels C and D, E and F, and G and H were acquired separately under different settings. However, panels E and F, and G and H were of the same section during the same session. All images are 4.5µm thick Z-stacks, deconvolved using Huygens Professional, and compressed into a two-dimensional image using Fiji/ImageJ Maximum Intensity Projections. All tissue pre-mortem.
