# Supplementary material for: Left ventricular myocardial molecular profile of human diabetic ischaemic cardiomyopathy
Source: EMBO Mol Med. 2025 Aug 4;17(9):2483–524. doi: 10.1038/s44321-025-00281-9 (PMC12423312; doi:10.1038/s44321-025-00281-9)
Supplement: Supplementary file 4 — Expanded View Figures [file 44321_2025_281_MOESM4_ESM.pdf]

# Expanded View Figures

**Figure EV1. Sample visualisation of normalised and $log_2$ transformed proteomic, metabolomic and lipidomic mass spectrometry data from human myocardium in all conditions.**

(**A–L**) Multidimensional scaling (MDS) plots, a tool to visualise high dimensional data in two dimensions, were used to iteratively separate samples (each point) in distance based on their dissimilarity of paired data (proteins, metabolites, and lipids; dimensions) calculated as the leading $log_2$ fold change (FC, average root-mean-square of the largest $log_2$ FCs). All protein/metabolite/lipid MDS plots are the same but vary in samples highlighted. (**A–C**) All conditions highlighted. (**D–F**) Heart failure (HF) conditions and healthy donors highlighted. (**G–I**) Ischaemic cardiomyopathy with diabetes (ICM-DM) and without diabetes (ICM-No DM) highlighted. (**J–L**) Non-ischaemic cardiomyopathy with diabetes (NICM-DM) and without diabetes (NICM-No DM) highlighted.

© The Author(s)

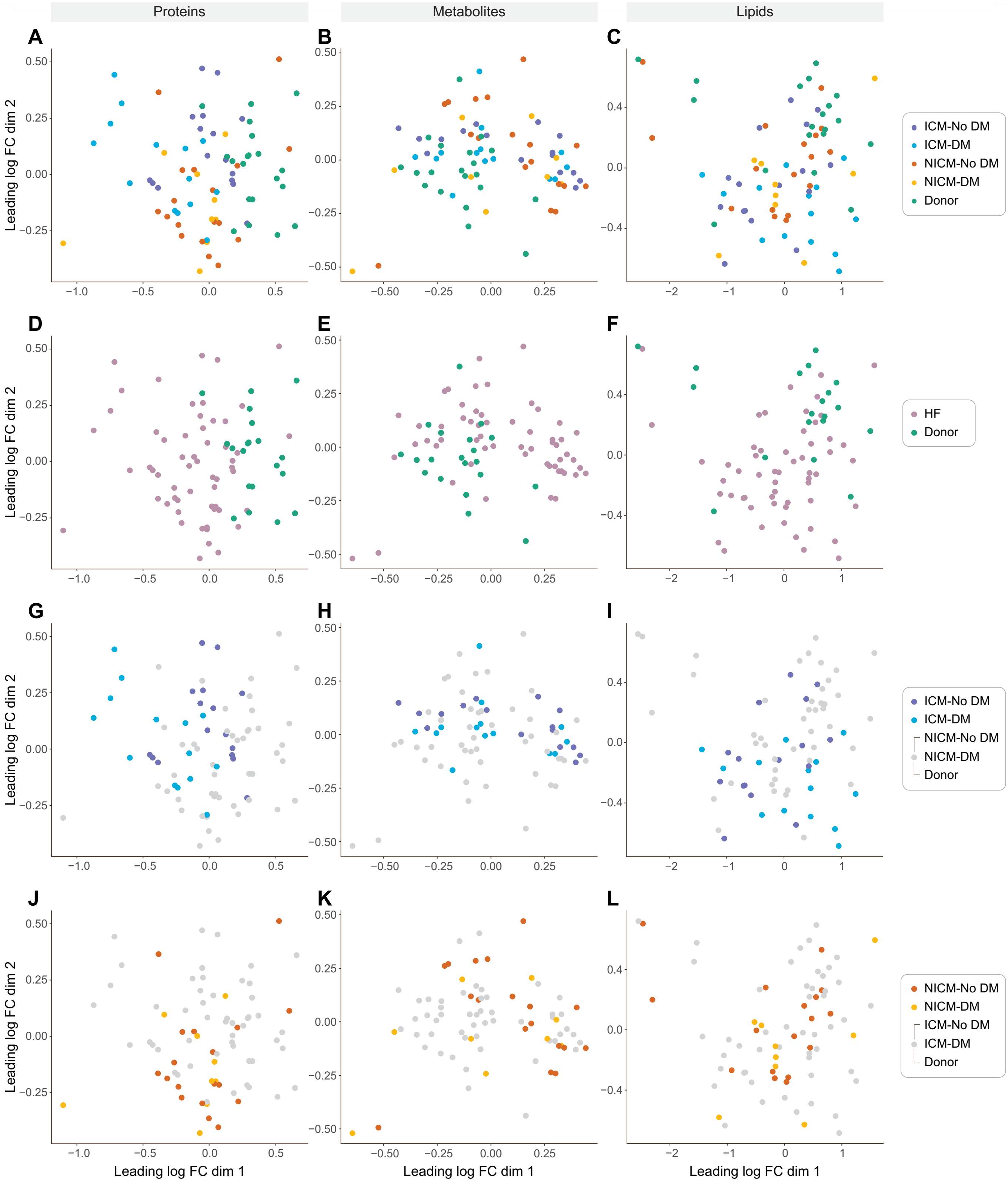
Proteins
Metabolites
Lipids
A
B
C
D
E
F
G
H
I
J
K
L
Leading log FC dim 2
Leading log FC dim 1
ICM-No DM
ICM-DM
NICM-No DM
NICM-DM
Donor
HF

 © The Author(s)

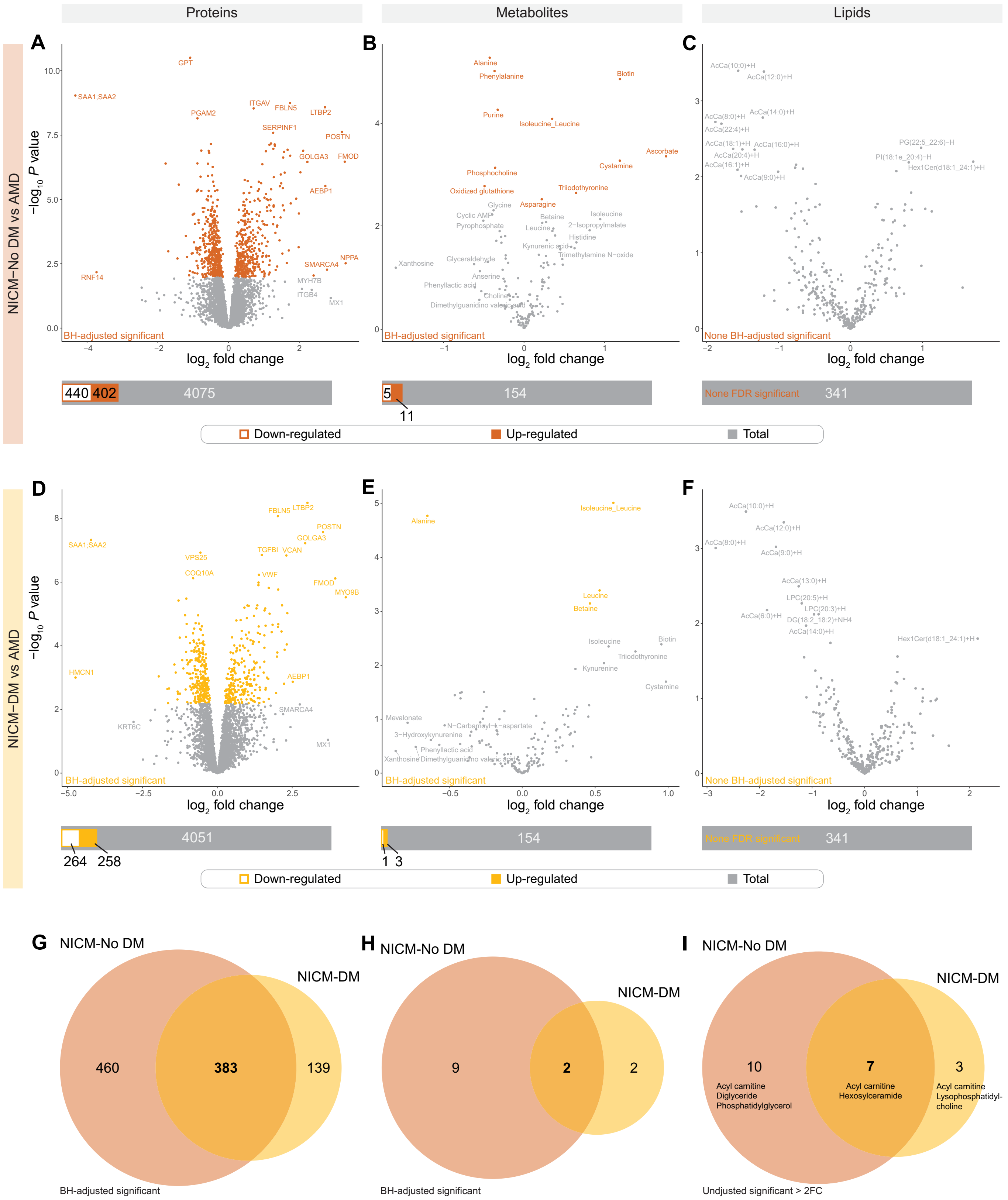

Proteins
Metabolites
Lipids
NICM-No DM vs AMD
A
B
C
−log10 P value
GPT
SAA1;SAA2
PGAM2
ITGAV
FBLN5
LTBP2
SERPINF1
POSTN
GOLGA3
FMOD
AEBP1
NPPA
SMARCA4
MYH7B
ITGB4
MX1
RNF14
BH-adjusted significant
log2 fold change
Alanine
Phenylalanine
Biotin
Purine
Isoleucine_Leucine
Ascorbate
Cystamine
Phosphocholine
Triiodothyronine
Oxidized glutathione
Glycine
Asparagine
Cyclic AMP
Pyrophosphate
Betaine
Leucine
Isoleucine
2−Isopropylmalate
Histidine
Kynurenic acid
Trimethylamine N−oxide
Xanthosine
Glyceraldehyde
Anserine
Phenyllactic acid
Choline
Dimethylguanidino valeric acid
AcCa(10:0)+H
AcCa(12:0)+H
AcCa(8:0)+H
AcCa(14:0)+H
AcCa(22:4)+H
AcCa(18:1)+H
AcCa(16:0)+H
AcCa(20:4)+H
AcCa(16:1)+H
AcCa(9:0)+H
PG(22:5_22:6)−H
PI(18:1e_20:4)−H
Hex1Cer(d18:1_24:1)+H
None BH-adjusted significant
440
402
4075
5
11
154
None FDR significant
341
Down-regulated
Up-regulated
Total
NICM−DM vs AMD
D
E
F
FBLN5
LTBP2
POSTN
GOLGA3
SAA1;SAA2
VPS25
TGFBI
VCAN
COQ10A
VWF
FMOD
MYO9E
HMCN1
AEBP1
KRT6C
SMARCA4
MX1
Isoleucine_Leucine
Alanine
Leucine
Betaine
Isoleucine
Biotin
Triiodothyronine
Kynurenine
Cystamine
Mevalonate
N−Carbamoyl−L−aspartate
3−Hydroxykynurenine
Phenyllactic acid
Xanthosine
Dimethylguanidino valeric acid
AcCa(10:0)+H
AcCa(12:0)+H
AcCa(8:0)+H
AcCa(9:0)+H
AcCa(13:0)+H
LPC(20:5)+H
LPC(20:3)+H
AcCa(6:0)+H
DG(18:2_18:2)+NH4
AcCa(14:0)+H
Hex1Cer(d18:1_24:1)+H
264
258
4051
1
3
154
341
G
NICM-No DM
NICM-DM
460
383
139
BH-adjusted significant
H
9
2
2
I
10
7
3
Acyl carnitine
Diglyceride
Phosphatidylglycerol
Acyl carnitine
Hexosylceramide
Acyl carnitine
Lysophosphatidyl-
choline
Undjusted significant > 2FC

◀ **Figure EV2. Human left ventricular myocardial differential analysis in protein, metabolite, and lipid abundance between non-ischaemic cardiomyopathy with (NICM-DM) and without diabetes (NICM-No DM) and age-matched donors (AMD).**

(**A–F**) Differential abundance was determined following Benjamini–Hochberg false discovery rate adjustment (FDR) of *P* values (FDR < 0.05). Analyses were performed using a moderated *t* test with the limma package (version 3.56.2) in R (version 4.3.1) following $\log_2$ transformation. NICM-DM $n = 9$, NICM-No DM $n = 18$ (proteomics and metabolomics) and 17 (lipidomics), AMD $n = 20$ (proteomics and metabolomics) and 19 (lipidomics). Superimposed bar plots summarise the number of significantly downregulated (white-filled bar) and upregulated (colour-filled bar) molecules relative to the total number of molecules analysed (grey bar). (**A–C**) NICM-No DM vs AMD. (**D–F**) NICM-DM vs AMD. (**G**, **H**) NICM-DM and NICM-No DM vs AMD FDR significant differentially abundant proteins and metabolites. (**I**) NICM-DM vs AMD and NICM-No DM vs AMD unadjusted significant ($P < 0.05$) lipids which were also > ±2-FC (in either direction) for comparative purposes (significance not accepted). Lipid classes annotated in order of the number of lipids which were unadjusted significant.

 © The Author(s)

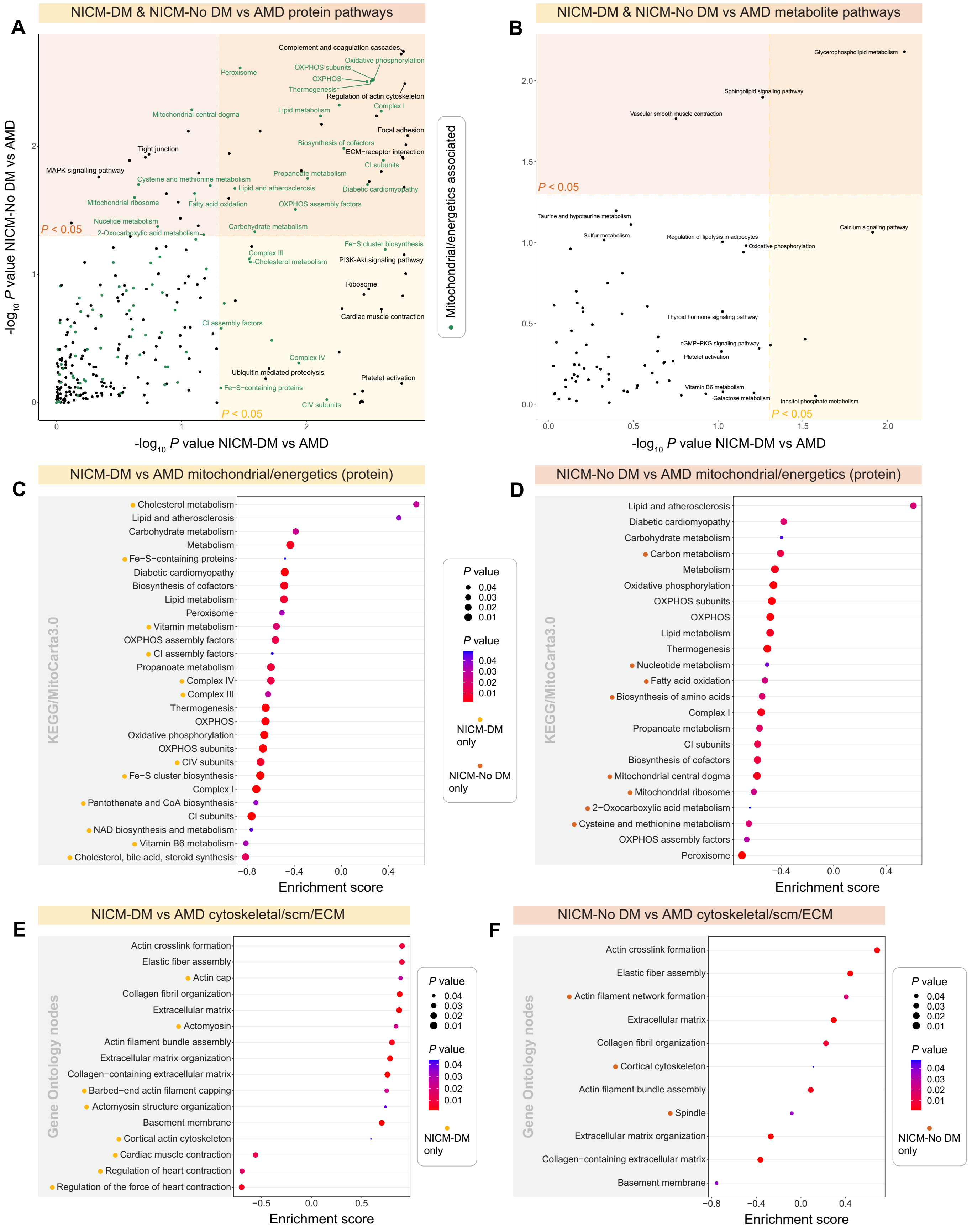
A
NICM-DM & NICM-No DM vs AMD protein pathways
Complement and coagulation cascades
Oxidative phosphorylation
Peroxisome
OXPHOS subunits
OXPHOS
Thermogenesis
Regulation of actin cytoskeleton
Complex I
Lipid metabolism
Mitochondrial central dogma
Focal adhesion
Biosynthesis of cofactors
ECM−receptor interaction
Tight junction
CI subunits
Propanoate metabolism
MAPK signalling pathway
Cysteine and methionine metabolism
Diabetic cardiomyopathy
Lipid and atherosclerosis
Mitochondrial ribosome
Fatty acid oxidation
OXPHOS assembly factors
Nucelide metabolism
Carbohydrate metabolism
2-Oxocarboxylic acid metabolism
Fe−S cluster biosynthesis
Complex III
Cholesterol metabolism
PI3K-Akt signaling pathway
Ribosome
Cardiac muscle contraction
CI assembly factors
Complex IV
Ubiquitin mediated proteolysis
Platelet activation
Fe−S−containing proteins
CIV subunits
P < 0.05
-log10 P value NICM-No DM vs AMD
-log10 P value NICM-DM vs AMD
Mitochondrial/energetics associated
B
NICM-DM & NICM-No DM vs AMD metabolite pathways
Glycerophospholipid metabolism
Sphingolipid signaling pathway
Vascular smooth muscle contraction
P < 0.05
Taurine and hypotaurine metabolism
Sulfur metabolism
Regulation of lipolysis in adipocytes
Calcium signaling pathway
Oxidative phosphorylation
Thyroid hormone signaling pathway
cGMP−PKG signaling pathway
Platelet activation
Vitamin B6 metabolism
Galactose metabolism
Inositol phosphate metabolism
-log10 P value NICM-DM vs AMD
C
NICM-DM vs AMD mitochondrial/energetics (protein)
KEGG/MitoCarta3.0
Cholesterol metabolism
Lipid and atherosclerosis
Carbohydrate metabolism
Metabolism
Fe−S−containing proteins
Diabetic cardiomyopathy
Biosynthesis of cofactors
Lipid metabolism
Peroxisome
Vitamin metabolism
OXPHOS assembly factors
CI assembly factors
Propanoate metabolism
Complex IV
Complex III
Thermogenesis
OXPHOS
Oxidative phosphorylation
OXPHOS subunits
CIV subunits
Fe−S cluster biosynthesis
Complex I
Pantothenate and CoA biosynthesis
CI subunits
NAD biosynthesis and metabolism
Vitamin B6 metabolism
Cholesterol, bile acid, steroid synthesis
Enrichment score
P value
NICM-DM only
NICM-No DM only
D
NICM-No DM vs AMD mitochondrial/energetics (protein)
KEGG/MitoCarta3.0
Lipid and atherosclerosis
Diabetic cardiomyopathy
Carbohydrate metabolism
Carbon metabolism
Metabolism
Oxidative phosphorylation
OXPHOS subunits
OXPHOS
Lipid metabolism
Thermogenesis
Nucleotide metabolism
Fatty acid oxidation
Biosynthesis of amino acids
Complex I
Propanoate metabolism
CI subunits
Biosynthesis of cofactors
Mitochondrial central dogma
Mitochondrial ribosome
2−Oxocarboxylic acid metabolism
Cysteine and methionine metabolism
OXPHOS assembly factors
Peroxisome
Enrichment score
E
NICM-DM vs AMD cytoskeletal/scm/ECM
Gene Ontology nodes
Actin crosslink formation
Elastic fiber assembly
Actin cap
Collagen fibril organization
Extracellular matrix
Actomyosin
Actin filament bundle assembly
Extracellular matrix organization
Collagen−containing extracellular matrix
Barbed−end actin filament capping
Actomyosin structure organization
Basement membrane
Cortical actin cytoskeleton
Cardiac muscle contraction
Regulation of heart contraction
Regulation of the force of heart contraction
Enrichment score
P value
NICM-DM only
F
NICM-No DM vs AMD cytoskeletal/scm/ECM
Gene Ontology nodes
Actin crosslink formation
Elastic fiber assembly
Actin filament network formation
Extracellular matrix
Collagen fibril organization
Cortical cytoskeleton
Actin filament bundle assembly
Spindle
Extracellular matrix organization
Collagen−containing extracellular matrix
Basement membrane
Enrichment score
P value
NICM-No DM only

**Figure EV3. Human non-ischaemic cardiomyopathy with (NICM-DM) and without diabetes (NICM-No DM) vs age-matched donor (AMD) myocardial protein and metabolite pathway analyses.**

(**A**, **B**) Scatter plots of -$\log_{10}$ *P* value enriched KEGG and MitoCarta3.0 protein pathways/gene sets of NICM-DM AMD and NICM-No DM where mitochondrial and energetics related pathways are coloured in green and significant ($P < 0.05$). Pathways in overlapping coloured regions were significant in both NICM-DM and NICM-No DM vs AMD. (**A**), Enriched pathways from the proteomic mass spectrometry (MS) analysis. (**B**) Enriched pathways from the metabolomic MS analysis. (**C**–**F**) Gene Set Enrichment Analysis (GSEA) enrichment bubble plots of top-ranked significantly enriched ($P < 0.05$) KEGG/MitoCarta3.0 pathways/gene sets and Gene Ontology nodes, respectively. (**C**, **D**) From (**A**). (**E**, **F**) Cytoskeletal, sarcomeric (scm), and extracellular matrix (ECM) enriched Gene Ontology Biological Process and Cellular Component nodes from selected parent nodes. Gene Set Enrichment Analysis (GSEA) analyses for (**A**–**F**) were performed on normalised and transformed proteomic and metabolomic datasets using clusterProfiler (version 4.8.1).

 © The Author(s)

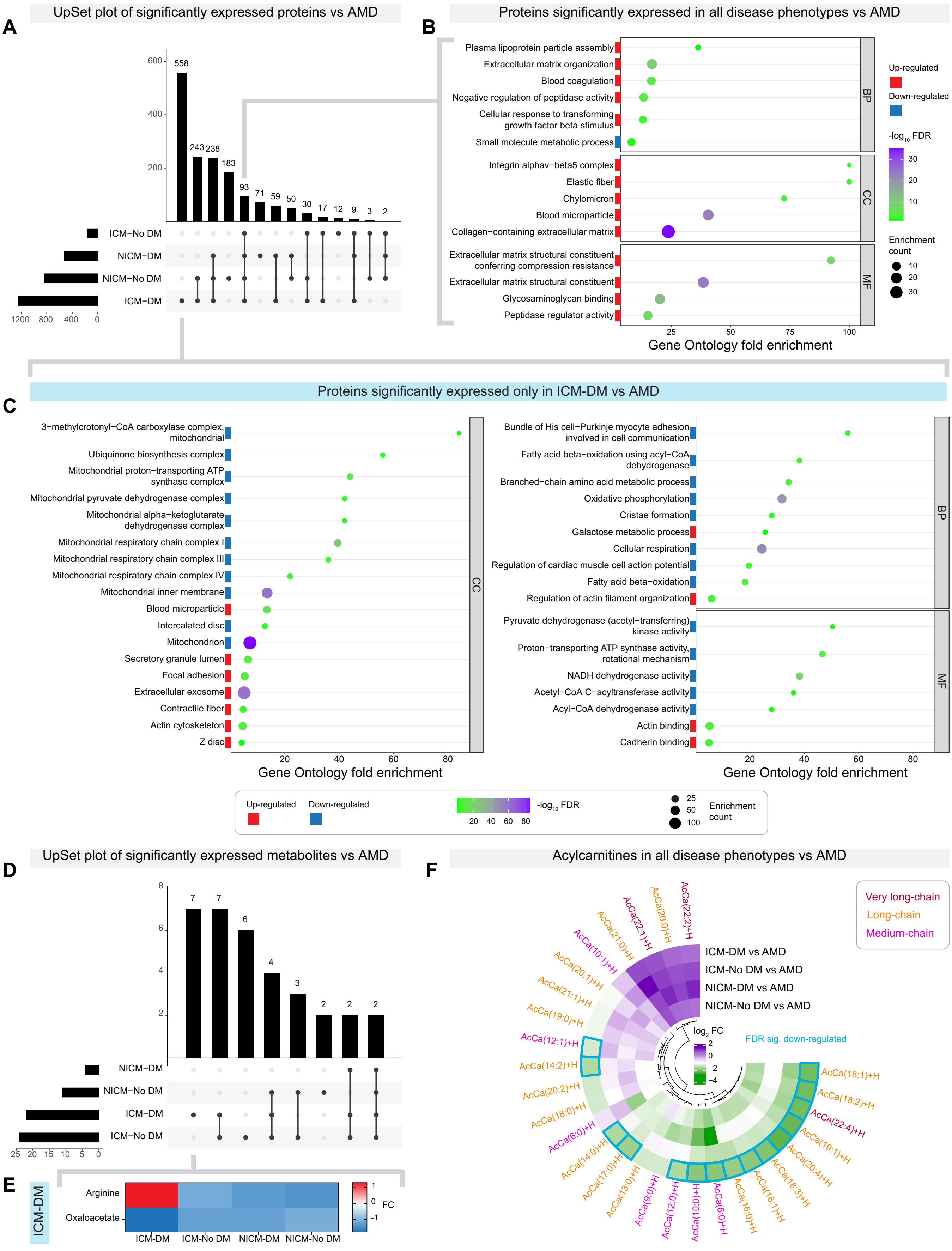
A
UpSet plot of significantly expressed proteins vs AMD
600
400
200
558
243
238
183
93
71
59
50
30
17
12
9
3
2
ICM–No DM
NICM–DM
NICM–No DM
ICM–DM
1200 800 400 0
B
Proteins significantly expressed in all disease phenotypes vs AMD
Plasma lipoprotein particle assembly
Extracellular matrix organization
Blood coagulation
Negative regulation of peptidase activity
Cellular response to transforming growth factor beta stimulus
Small molecule metabolic process
Integrin alphav−beta5 complex
Elastic fiber
Chylomicron
Blood microparticle
Collagen−containing extracellular matrix
Extracellular matrix structural constituent conferring compression resistance
Extracellular matrix structural constituent
Glycosaminoglycan binding
Peptidase regulator activity
BP
CC
MF
25 50 75 100
Gene Ontology fold enrichment
Up-regulated
Down-regulated
-log10 FDR
30
20
10
Enrichment count
10
20
30
C
Proteins significantly expressed only in ICM-DM vs AMD
3−methylcrotonyl−CoA carboxylase complex, mitochondrial
Ubiquinone biosynthesis complex
Mitochondrial proton−transporting ATP synthase complex
Mitochondrial pyruvate dehydrogenase complex
Mitochondrial alpha−ketoglutarate dehydrogenase complex
Mitochondrial respiratory chain complex I
Mitochondrial respiratory chain complex III
Mitochondrial respiratory chain complex IV
Mitochondrial inner membrane
Blood microparticle
Intercalated disc
Mitochondrion
Secretory granule lumen
Focal adhesion
Extracellular exosome
Contractile fiber
Actin cytoskeleton
Z disc
CC
20 40 60 80
Gene Ontology fold enrichment
Bundle of His cell−Purkinje myocyte adhesion involved in cell communication
Fatty acid beta−oxidation using acyl−CoA dehydrogenase
Branched−chain amino acid metabolic process
Oxidative phosphorylation
Cristae formation
Galactose metabolic process
Cellular respiration
Regulation of cardiac muscle cell action potential
Fatty acid beta−oxidation
Regulation of actin filament organization
Pyruvate dehydrogenase (acetyl−transferring) kinase activity
Proton−transporting ATP synthase activity, rotational mechanism
NADH dehydrogenase activity
Acetyl−CoA C−acyltransferase activity
Acyl−CoA dehydrogenase activity
Actin binding
Cadherin binding
BP
MF
20 40 60 80
Gene Ontology fold enrichment
Up-regulated
Down-regulated
-log10 FDR
20 40 60 80
25
50
100
Enrichment count
D
UpSet plot of significantly expressed metabolites vs AMD
8
6
4
2
7
7
6
4
3
2
2
2
NICM–DM
NICM–No DM
ICM–DM
ICM–No DM
25 20 15 10 5 0
E
ICM-DM
Arginine
Oxaloacetate
ICM-DM
ICM-No DM
NICM-DM
NICM-No DM
1
0
-1
FC
F
Acylcarnitines in all disease phenotypes vs AMD
Very long-chain
Long-chain
Medium-chain
ICM-DM vs AMD
ICM-No DM vs AMD
NICM-DM vs AMD
NICM-No DM vs AMD
log2 FC
2
0
−2
−4
FDR sig. down-regulated
AcCa(22:2)+H
AcCa(20:0)+H
AcCa(22:1)+H
AcCa(21:0)+H
AcCa(10:1)+H
AcCa(20:1)+H
AcCa(21:1)+H
AcCa(19:0)+H
AcCa(12:1)+H
AcCa(14:2)+H
AcCa(20:2)+H
AcCa(18:0)+H
AcCa(6:0)+H
AcCa(14:0)+H
AcCa(17:0)+H
AcCa(13:0)+H
AcCa(9:0)+H
AcCa(12:0)+H
AcCa(10:0)+H
AcCa(8:0)+H
AcCa(16:0)+H
AcCa(16:1)+H
AcCa(18:3)+H
AcCa(20:4)+H
AcCa(19:1)+H
AcCa(22:4)+H
AcCa(18:2)+H
AcCa(18:1)+H

**Figure EV4. Myocardial differential abundance co-analyses of all heart failure conditions vs age-matched donors (AMD), relative to each other, for proteomic, metabolomic, and lipidomic mass spectrometry analyses, with a focus on ischaemic cardiomyopathy with diabetes (ICM-DM).**

(**A**) UpSet plot summarising significant differentially expressed proteins in all the heart failure conditions; ICM-DM, ICM without diabetes (ICM-No DM), non-ischaemic cardiomyopathy with diabetes (NICM-DM), and NICM without diabetes (NICM-No DM), vs AMD from proteomic mass spectrometry (MS). Statistical significance of differential expression was determined following Benjamini–Hochberg false discovery rate adjustment (FDR) of *P* values (FDR < 0.05). (**B**) Gene Ontology (GO) analysis by PANTHER (http://geneontology.org/, PANTHER17.0) enrichment bubble plot showing selected significantly enriched Biological Process (BP), Cellular Component (CC), and Molecular Function (MF) nodes from proteins which were significantly differentially expressed in all heart failure conditions vs AMD. Nodes with an FDR < 0.05 were considered statistically significant. This plot was the combination of two separate GO analyses; one from downregulated proteins compared to AMD (blue) and one from upregulated proteins compared to AMD (red). Enrichment count, represented as the size of the bubble, is the number of significant proteins in that particular node. GO fold enrichment is calculated as observed enrichment count/expected enrichment count from a random set of gene symbols of equal an input set size. (**C**) GO analysis by PANTHER enrichment bubble plot of selected significantly enriched nodes from proteins which were significantly expressed only in ICM-DM vs AMD. (**D**) UpSet plot summarising FDR significant differentially abundant metabolites in all the heart failure conditions vs AMD from metabolomic MS. (**E**) Heat map of the two greatest fold change (FC) metabolites in, and specific to, ICM-DM vs AMD. (**F**) Circular heatmap of all heart failure conditions $\log_2$ FC vs AMD quantified acylcarnitines from lipidomic MS where FDR significant differentially abundant lipids are indicated. A Lipid's chain length was contrasted to others in colour; very-long-chain (22 or more carbon length, maroon), long-chain (13–21 carbon length, orange), and medium-chain (6–12 carbon length, pink). Short-chain acylcarnitines (2–5 carbon length) were quantified in metabolomic MS.

 © The Author(s)

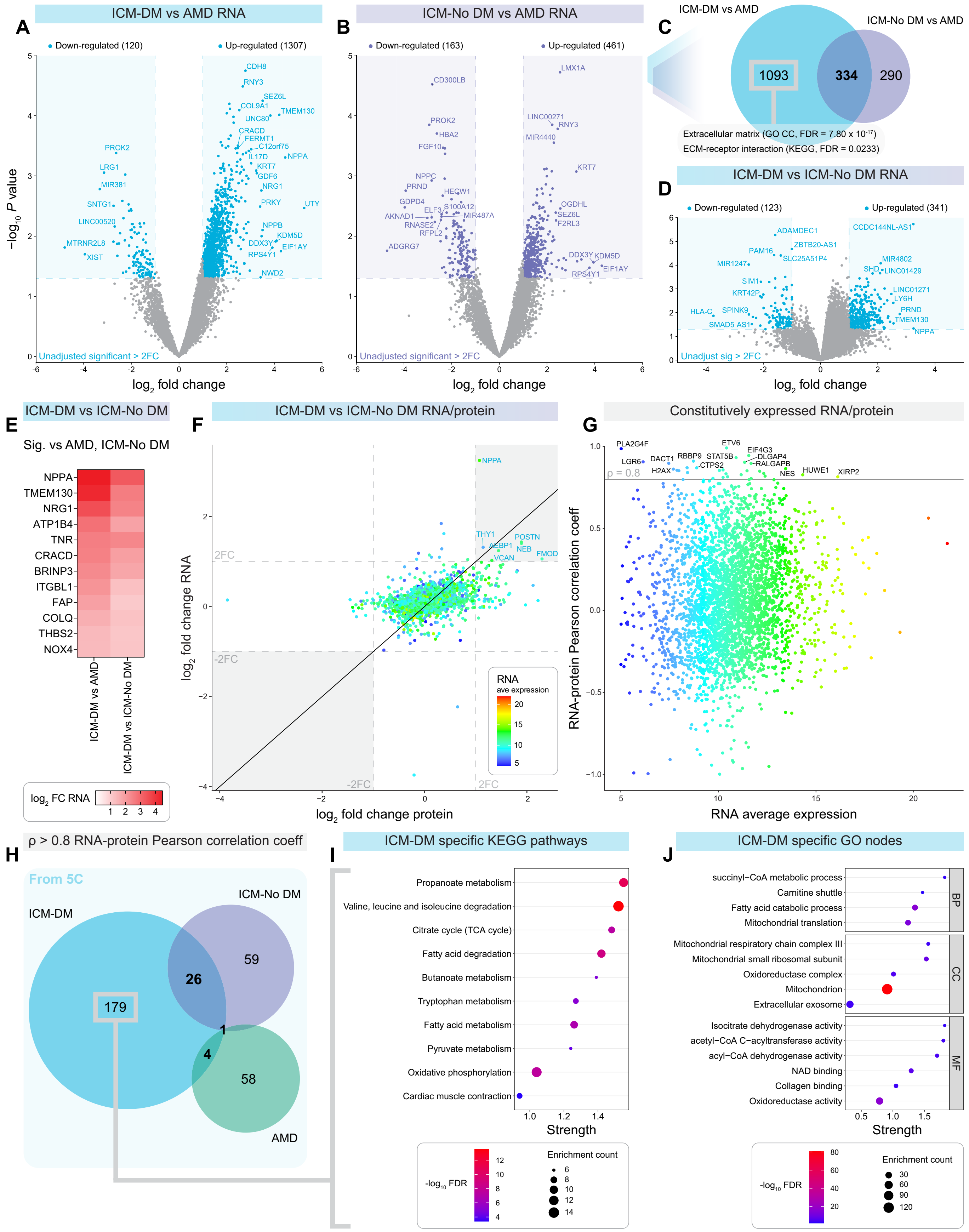
A
ICM-DM vs AMD RNA
Down-regulated (120)
Up-regulated (1307)
Unadjusted significant > 2FC
B
ICM-No DM vs AMD RNA
Down-regulated (163)
Up-regulated (461)
C
ICM-DM vs AMD
ICM-No DM vs AMD
1093
334
290
Extracellular matrix (GO CC, FDR = 7.80 x 10-17)
ECM-receptor interaction (KEGG, FDR = 0.0233)
D
ICM-DM vs ICM-No DM RNA
Down-regulated (123)
Up-regulated (341)
Unadjust sig > 2FC
E
ICM-DM vs ICM-No DM
Sig. vs AMD, ICM-No DM
NPPA
TMEM130
NRG1
ATP1B4
TNR
CRACD
BRINP3
ITGBL1
FAP
COLQ
THBS2
NOX4
ICM-DM vs AMD
ICM-DM vs ICM-No DM
log2 FC RNA
F
ICM-DM vs ICM-No DM RNA/protein
log2 fold change RNA
log2 fold change protein
RNA ave expression
G
Constitutively expressed RNA/protein
RNA-protein Pearson correlation coeff
RNA average expression
H
ρ > 0.8 RNA-protein Pearson correlation coeff
From 5C
ICM-DM
ICM-No DM
AMD
179
26
59
1
4
58
I
ICM-DM specific KEGG pathways
Propanoate metabolism
Valine, leucine and isoleucine degradation
Citrate cycle (TCA cycle)
Fatty acid degradation
Butanoate metabolism
Tryptophan metabolism
Fatty acid metabolism
Pyruvate metabolism
Oxidative phosphorylation
Cardiac muscle contraction
Strength
-log10 FDR
Enrichment count
J
ICM-DM specific GO nodes
succinyl−CoA metabolic process
Carnitine shuttle
Fatty acid catabolic process
Mitochondrial translation
Mitochondrial respiratory chain complex III
Mitochondrial small ribosomal subunit
Oxidoreductase complex
Mitochondrion
Extracellular exosome
Isocitrate dehydrogenase activity
acetyl−CoA C−acyltransferase activity
acyl−CoA dehydrogenase activity
NAD binding
Collagen binding
Oxidoreductase activity
BP
CC
MF
Strength
-log10 FDR
Enrichment count

**Figure EV5. Human left ventricular myocardium RNA sequencing analyses with protein co-expression and correlation in ischaemic cardiomyopathy (ICM) and age-matched donors (AMD).**

(**A**, **B**) Differential statistical significance was determined as unadjusted significant ($P = 0.05$) and > ±2-fold change (FC). Higher FC significant sequences, written as gene symbols, were annotated. (**A**), ICM with diabetes (ICM-DM) vs AMD. (**B**) ICM without diabetes (ICM-No DM) vs AMD. (**C**) Summary Venn diagram of (**A**, **B**). Sequences which were only significant in ICM-DM were analysed in STRING (https://string-db.org/, version 12.0) which revealed that the Extracellular matrix Gene Ontology (GO) Cellular Component (CC) node and the ECM-receptor interaction KEGG pathway were among the highest significantly enriched nodes/pathways. Nodes/pathways with a Benjamini–Hochberg false discovery rate adjusted *P* value (FDR) < 0.05 were determined as significant. (**D**) ICM-DM vs ICM-No DM RNA. Down and upregulation was defined as significance in ICM-DM compared to ICM-No DM. (**E**) Summary heat map of RNA sequences of biological interest from (**D**) which were both significant in ICM-DM vs AMD and ICM-No DM. (**F**) RNA and protein $\log_2$ FC plot of ICM-DM vs ICM-No DM where gene symbols which are >2-FC in both protein and RNA are annotated. ICM-DM average RNA expression represented on a colour scale. (**G**) RNA–protein Pearson correlation plot of ICM-DM, ICM-No DM, and AMD combined to identify common constitutively expressed RNA/protein whereby a correlation coefficient of $\rho > 0.8$ was accepted as being correlated. Highest correlated gene symbols were annotated. Gene symbols were spread out across the x-axis according to average RNA expression of all the groups combined. (**H**) From (**C**), showing gene symbols which had an RNA–protein Pearson correlation coefficient of $\rho > 0.8$ in ICM-DM, ICM-No DM and AMD. (**I**, **J**) STRING (https://version-11-5.string-db.org/, version 11.5) analysis enrichment bubble plots of selected FDR significant (FDR < 0.05) KEGG pathways and Gene Ontology (GO) nodes from gene symbols which were RNA–protein correlated only in ICM-DM. Strength of the enriched pathways/nodes were calculated as $\log_{10}$(observed enrichment count/expected enrichment count from a random set of gene symbols of equal an input set size) wherein the enrichment count (represented as the size of the bubble) was the number of significant differentially expressed gene symbols in that particular node. (**I**) Enriched KEGG pathways. (**J**) Enriched GO Biological Process (BP), CC, and Molecular Function (MF) nodes. Differential analyses for (**A**, **B**, **D**) were performed using a moderated *t* test with the limma package (version 3.56.2) in R (version 4.3.1) following $\log_2$ transformation. ICM-DM $n = 7$, ICM-No DM $n = 7$, AMD $n = 7$.


© The Author(s)

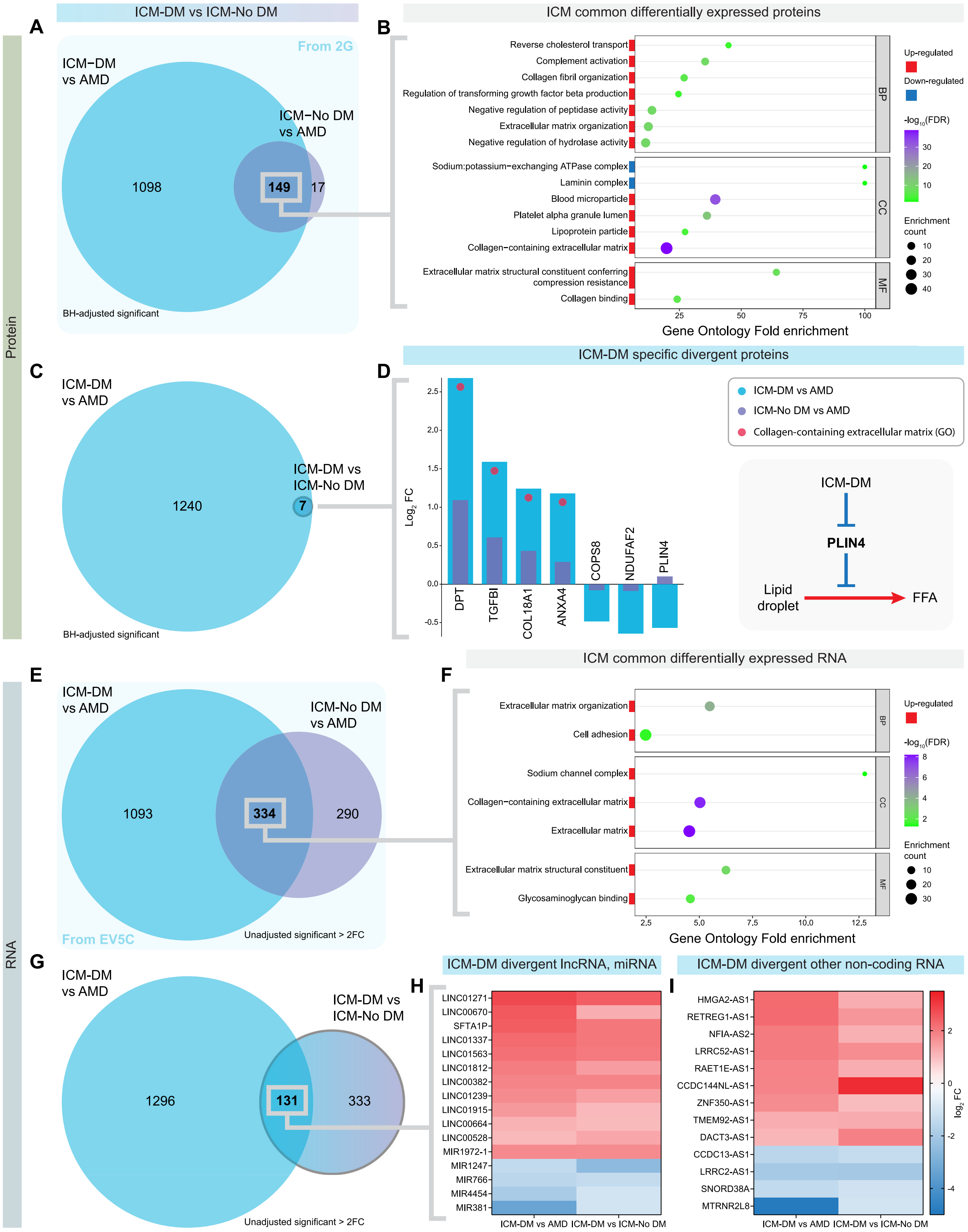

ICM-DM vs ICM-No DM
ICM common differentially expressed proteins
A
From 2G
ICM−DM vs AMD
ICM−No DM vs AMD
1098
149
17
BH-adjusted significant
B
Reverse cholesterol transport
Complement activation
Collagen fibril organization
Regulation of transforming growth factor beta production
Negative regulation of peptidase activity
Extracellular matrix organization
Negative regulation of hydrolase activity
Sodium:potassium−exchanging ATPase complex
Laminin complex
Blood microparticle
Platelet alpha granule lumen
Lipoprotein particle
Collagen−containing extracellular matrix
Extracellular matrix structural constituent conferring compression resistance
Collagen binding
BP
CC
MF
Gene Ontology Fold enrichment
Up-regulated
Down-regulated
-log10(FDR)
Enrichment count
Protein
ICM-DM specific divergent proteins
C
ICM-DM vs AMD
ICM-DM vs ICM-No DM
1240
7
BH-adjusted significant
D
Log2 FC
DPT
TGFBI
COL18A1
ANXA4
COPS8
NDUFAF2
PLIN4
ICM-DM vs AMD
ICM-No DM vs AMD
Collagen-containing extracellular matrix (GO)
ICM-DM
PLIN4
Lipid droplet
FFA
ICM common differentially expressed RNA
E
ICM-DM vs AMD
ICM-No DM vs AMD
1093
334
290
From EV5C
Unadjusted significant > 2FC
F
Extracellular matrix organization
Cell adhesion
Sodium channel complex
Collagen−containing extracellular matrix
Extracellular matrix
Extracellular matrix structural constituent
Glycosaminoglycan binding
Gene Ontology Fold enrichment
RNA
G
ICM-DM vs AMD
ICM-DM vs ICM-No DM
1296
131
333
Unadjusted significant > 2FC
ICM-DM divergent lncRNA, miRNA
H
LINC01271
LINC00670
SFTA1P
LINC01337
LINC01563
LINC01812
LINC00382
LINC01239
LINC01915
LINC00664
LINC00528
MIR1972-1
MIR1247
MIR766
MIR4454
MIR381
ICM-DM vs AMD
ICM-DM vs ICM-No DM
ICM-DM divergent other non-coding RNA
I
HMGA2-AS1
RETREG1-AS1
NFIA-AS2
LRRC52-AS1
RAET1E-AS1
CCDC144NL-AS1
ZNF350-AS1
TMEM92-AS1
DACT3-AS1
CCDC13-AS1
LRRC2-AS1
SNORD38A
MTRNR2L8
ICM-DM vs AMD
ICM-DM vs ICM-No DM
log2 FC

**Figure EV6. Human ischaemic cardiomyopathy (ICM) common differentially expressed myocardial proteins and RNA, and ICM with diabetes (ICM-DM) specific divergent proteins and RNA.**

(**A**) From Fig. 2G of ICM-DM and ICM without diabetes (ICM-No DM) vs AMD significant differentially expressed proteins. Statistical significance was determined following Benjamini–Hochberg false discovery rate adjustment (FDR) of *P* values (FDR < 0.05). (**B**) Gene Ontology (GO) analysis by PANTHER (http://geneontology.org/, PANTHER17.0) enrichment bubble plot showing selected significantly enriched Biological Process (BP), Cellular Component (CC), and Molecular Function (MF) nodes from proteins which were significantly differentially expressed in both ICM-DM and ICM-No DM vs AMD. Nodes with an FDR < 0.05 were considered statistically significant. This plot was the combination of two separate GO analyses; one from downregulated proteins compared to AMD (blue) and one from upregulated proteins compared to AMD (red). Enrichment count, represented as the size of the bubble, is the number of significant proteins in that particular node. GO fold enrichment is calculated as observed enrichment count/expected enrichment count from a random set of gene symbols of equal an input set size. (**C**) ICM-DM vs AMD and ICM-DM vs ICM-No DM. (**D**) A $\log_2$ fold change (FC) bar plot of ICM-DM and ICM-No DM vs AMD of proteins found to be significant in ICM-DM vs ICM-No DM (ICM-DM-specific and divergent from ICM-No DM). PLIN4, which was reduced in ICM-DM vs AMD while increased in ICM-No DM vs AMD, negatively regulates free fatty acid (FFA) availability. (**E**) Summary Venn diagram from Fig. EV5C of ICM-DM and ICM-No DM vs AMD significant differentially expressed RNA. Significance of RNA expression was determined as unadjusted significant ($P = 0.05$) and ±2-FC. (**F**) GO analysis by PANTHER of significant differentially expressed RNA in both ICM-DM and ICM-No DM vs AMD. (**G**) Venn diagram of significantly expressed RNA in ICM-DM vs AMD and ICM-DM vs ICM-No DM. (**H**) Long noncoding and microRNA sequences, and (**I**) other noncoding RNA significant in ICM-DM vs AMD and ICM-DM vs ICM-No DM in the same direction. Scale bar is shared between (**H**) and (**I**).

 © The Author(s)

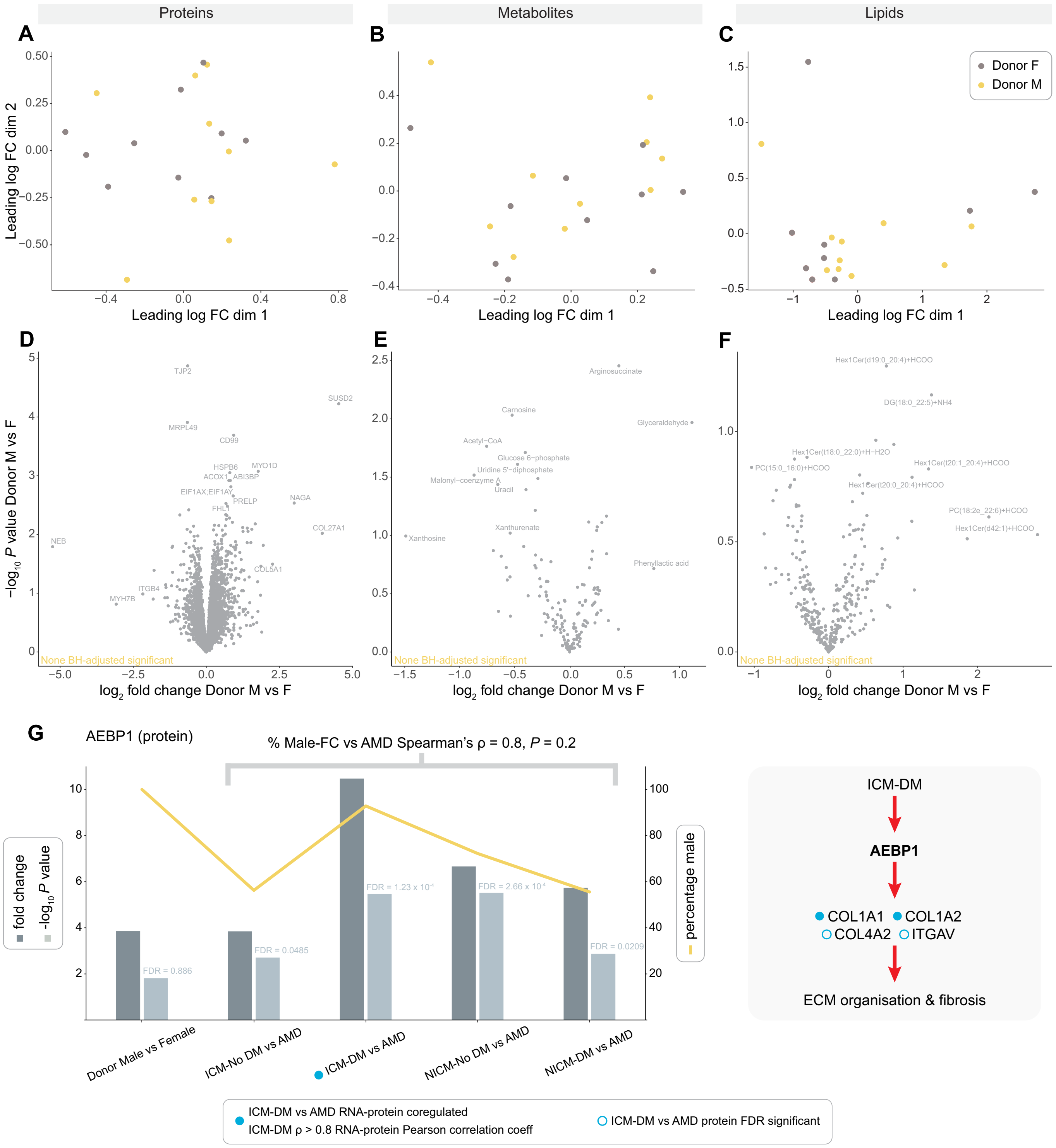
Proteins
Metabolites
Lipids
A
B
C
Donor F
Donor M
Leading log FC dim 1
Leading log FC dim 2
D
E
F
None BH-adjusted significant
log2 fold change Donor M vs F
−log10 P value Donor M vs F
G
AEBP1 (protein)
% Male-FC vs AMD Spearman's ρ = 0.8, P = 0.2
fold change
-log10 P value
percentage male
FDR = 0.886
FDR = 0.0485
FDR = 1.23 x 10-4
FDR = 2.66 x 10-4
FDR = 0.0209
Donor Male vs Female
ICM-No DM vs AMD
ICM-DM vs AMD
NICM-No DM vs AMD
NICM-DM vs AMD
ICM-DM
AEBP1
COL1A1
COL1A2
COL4A2
ITGAV
ECM organisation & fibrosis
ICM-DM vs AMD RNA-protein coregulated
ICM-DM ρ > 0.8 RNA-protein Pearson correlation coeff
ICM-DM vs AMD protein FDR significant

**Figure EV7. Healthy donor male myocardium vs female and potential male-dominant influence in heart failure.**

(**A–C**) Multidimensional scaling (MDS) plots, a tool to visualise high dimensional data in two dimensions, were used to iteratively separate samples (each point) in distance based on their dissimilarity of paired data (proteins, metabolites, and lipids; dimensions) calculated as the leading $\log_2$ fold change (FC, average root-mean-square of the largest $\log_2$ FCs). Healthy donor males represented as yellow, females as grey. (**D–F**) Volcano plots of male vs female molecule (proteins, metabolites, and lipids, respectively) abundance whereby significance was determined after Benjamini–Hochberg false discovery rate adjustment (FDR) of $P$ values (FDR < 0.05). The was no significance in abundance between donor males and females. Analyses were performed using a moderated $t$ test with the limma package (version 3.56.2) in R (version 4.3.1) following $\log_2$ transformation. AMD males $n = 10$, AMD females $n = 10$ (proteomics and metabolomics) and 9 (lipidomics). (**G**) Bar plot of protein AEBP1 fold change (dark grey) and $\log_{10}$ $P$ value (light grey, with FDR-adjusted value annotated) in donor males vs females, and heart failure conditions vs age-matched donors (AMD). Superimposed line plot shows the associated percentage of males in the subject group; the donor male group are 100% male. AEBP1 was FDR significant differentially expressed in all HF conditions vs AMD but the most in ICM-DM, which also had the highest FC. The ICM-DM group had the highest percentage of males. A male-protein FC vs AMD nonparametric Spearman's correlation test was performed on the HF conditions to determine a possible significant male relationship to AEBP1 FC. Significance was determined as $\rho > 0.8$ and $P < 0.05$. AEBP1 has been identified to positively regulate extracellular matrix (ECM) organisation and fibrosis, and was co-regulated in ICM-DM vs AMD in both RNA and protein, and RNA–protein correlated in ICM-DM.

 © The Author(s)

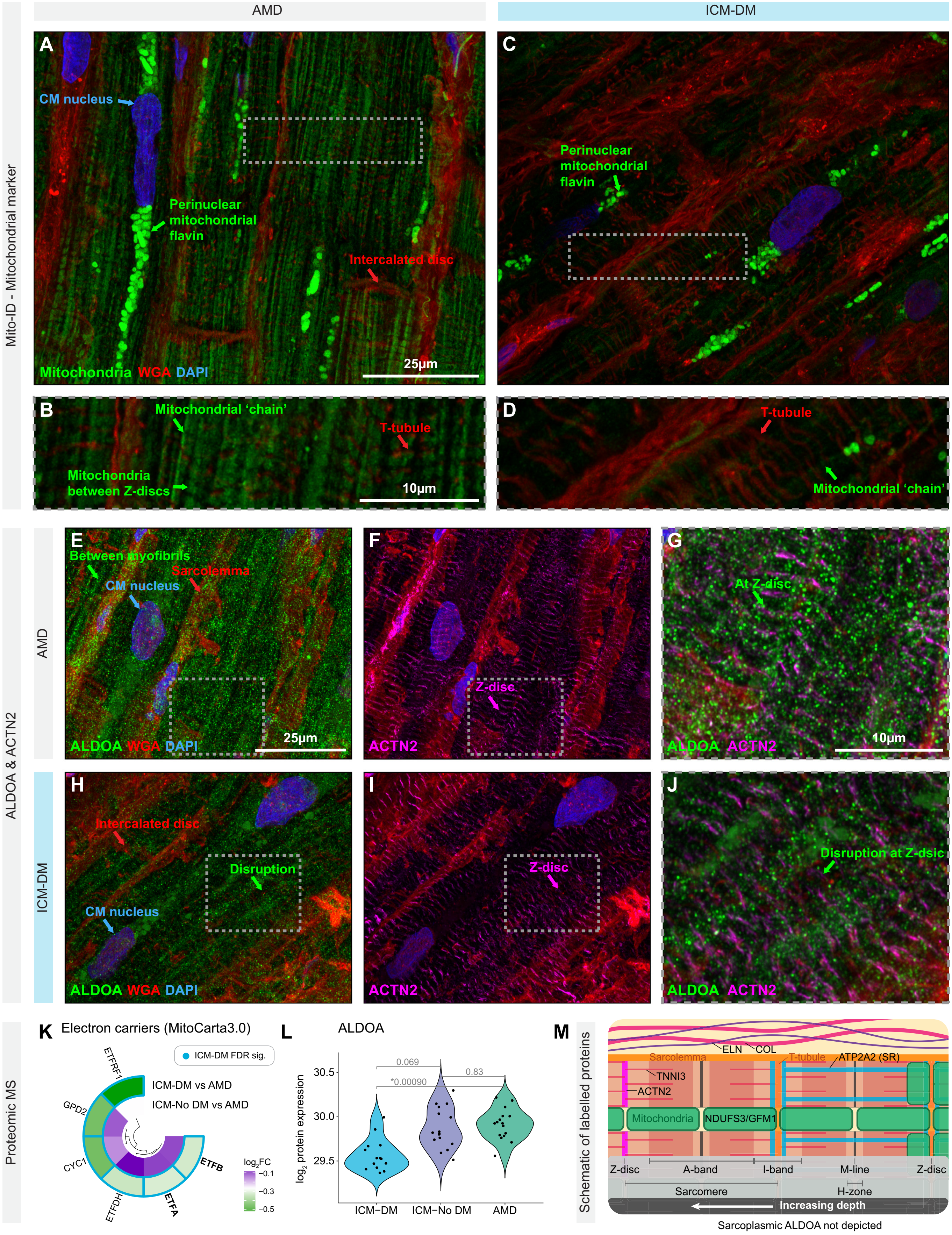
AMD
ICM-DM
Mito-ID - Mitochondrial marker
A
CM nucleus
Perinuclear mitochondrial flavin
Intercalated disc
Mitochondria WGA DAPI
25µm
C
Perinuclear mitochondrial flavin
B
Mitochondrial 'chain'
T-tubule
Mitochondria between Z-discs
10µm
D
T-tubule
Mitochondrial 'chain'
ALDOA & ACTN2
E
Between myofibrils
Sarcolemma
CM nucleus
ALDOA WGA DAPI
25µm
F
Z-disc
ACTN2
G
At Z-disc
ALDOA ACTN2
10µm
H
Intercalated disc
Disruption
CM nucleus
ALDOA WGA DAPI
I
Z-disc
ACTN2
J
Disruption at Z-dsic
ALDOA ACTN2
Proteomic MS
K Electron carriers (MitoCarta3.0)
ICM-DM FDR sig.
ICM-DM vs AMD
ICM-No DM vs AMD
ETFRF1
GPD2
CYC1
ETFDH
ETFA
ETFB
log2FC
−0.1
−0.3
−0.5
L ALDOA
log2 protein expression
30.5
30.0
29.5
0.069
0.83
*0.00090
ICM−DM
ICM−No DM
AMD
M
Schematic of labelled proteins
ELN
COL
Sarcolemma
T-tubule
ATP2A2 (SR)
TNNI3
ACTN2
Mitochondria
NDUFS3/GFM1
Z-disc
A-band
I-band
M-line
Z-disc
Sarcomere
H-zone
Increasing depth
Sarcoplasmic ALDOA not depicted

**Figure EV8. Immunofluorescent labelling of mitochondria and sarcoplasmic ALDOA in cryopreserved ischaemic cardiomyopathy with diabetes (ICM-DM) and healthy age-matched donor (AMD) myocardium.**

(**A–D**) Immunofluorescent confocal microscopy images of cryopreserved human myocardium representing qualitative differences between AMD and ICM-DM in mitochondria labelled with Mito-ID. Bright (autofluorescent) perinuclear mitochondrial flavin identified. (**E–J**) Labelled muscle aldolase (ALDOA, green), localised in the sarcoplasm, with a labelled Z-disc protein, ACTN2 (magenta). Membranes, particularly the sarcolemma, were stained with fluorophore-conjugated wheat germ agglutinin (red) and nuclei were stained using DAPI (blue). Cardiomyocytes (CM) are depicted in a longitudinal orientation. All images are 4.5 μm-thick Z-stacks, deconvolved using Huygens Professional, and compressed into a two-dimensional image using Fiji/ImageJ Maximum Intensity Projections. (**K**) Proteomic mass spectrometry (MS) $log_2FC$ circular heatmap of quantified proteins from the MitoCarta3.0 electron carriers gene set. (**L**) Violin plot of proteomic MS $log_2$ transformed quantification of ALDOA in ICM-DM, ICM without diabetes (ICM-No DM), and AMD groups. FDR-adjusted *P* values were annotated to reveal significant differences (*, FDR < 0.05) between groups. Analyses were performed using a moderated *t* test with the limma package (version 3.56.2) in R (version 4.3.1) following $log_2$ transformation. ICM-DM $n = 14$, ICM-No DM $n = 16$, AMD $n = 20$. (**M**) Cellular schematic showing localisation of all histologically labelled proteins in this study. Sarcoplasmic ALDOA not depicted. SR sarcoplasmic reticulum. All tissue was pre-mortem. Macroscopic scar tissue, particularly in heart failure conditions, was avoided in all quantitative analyses (mass spectrometry and RNA sequencing) and imaging. Only the most normal/healthy appearing and longitudinally oriented AMD and ICM-DM cardiomyocytes were imaged in confocal microscopy.

 © The Author(s)
